# The microbiota of healthy dogs demonstrates individualized responses to synbiotic supplementation in a randomized controlled trial

**DOI:** 10.1186/s42523-021-00098-0

**Published:** 2021-05-10

**Authors:** Jirayu Tanprasertsuk, Aashish R. Jha, Justin Shmalberg, Roshonda B. Jones, LeeAnn M. Perry, Heather Maughan, Ryan W. Honaker

**Affiliations:** 1 NomNomNow Inc, Nashville, TN 37207 USA; 2 Genetic Heritage Group, Department of Biology, New York University Abu Dhabi, Abu Dhabi, UAE; 3 Department of Comparative, Diagnostic, and Population Medicine, College of Veterinary Medicine, University of Florida, Gainesville, FL 32611 USA; 4 Ronin Institute, Montclair, NJ 07043 USA

**Keywords:** Diarrhea, Gastrointestinal health, Probiotics, Inulin, Synbiotics, Dogs, Microbiome

## Abstract

**Background:**

Probiotics have been demonstrated to ameliorate clinical signs of gastrointestinal diseases in dogs in various studies. However, the effect of probiotics in a healthy population, as well as factors contributing individualized responses, remain largely unknown. This trial examined gut microbiota (GM) and health outcomes in household dogs after synbiotic (SN) supplementation containing probiotics and inulin (a prebiotic). Healthy dogs were randomized to receive SN (50 mg/d inulin and 20 billion total CFU/d of *L. reuteri*, *P. acidilactici*, *E. faecium*, *L. acidophilus*, *B. animalis*, *L. fermentum*, *L. rhamnosus*) or placebo (PL) for 4 weeks. Owners completed a health survey and collected stool samples for GM profiling (shotgun metagenomic sequencing) at baseline and week 4 in both groups, and at week 6 in the SN group.

**Results:**

A significant shift (*p* < 0.001) in β-diversity was observed in the SN (*n* = 24), but not PL group (*n* = 19), at week 4 relative to baseline. Forty-five bacterial species, 43 (96%) of which were *Lactobacillales*, showed an increase in the relative abundances (≥2 fold change, adjusted *p* < 0.05) in the SN group at week 4. *E. coli* also decreased at week 4 in the SN group (2.8-fold, adjusted *p* < 0.01). The altered taxa largely returned to baseline at week 6. The degree of changes in β-diversity was associated with GM at baseline. Specifically, dogs with higher *Proteobacteria* and lower *Lactobacillales* responded more robustly to supplementation in terms of the change in β-diversity. Dogs fed SN tended to have lower diarrhea incidence (0% vs 16%, *p* = 0.08).

**Conclusions:**

SN supplement had a short-term impact on the gut microbiota in healthy household dogs as characterized by shotgun metagenomic sequencing. Findings warrant further investigation with longer duration and populations at risk of gastrointestinal diseases. The magnitude of response to the supplement was associated with microbial profile at baseline. To our knowledge, this is the first study documenting such association and may provide a basis for personalized nutrition in companion dogs.

**Supplementary Information:**

The online version contains supplementary material available at 10.1186/s42523-021-00098-0.

## Background

Probiotics are live bacterial cells that are ingested, frequently with the goal of maintaining or improving gastrointestinal (GI) health. Probiotics can reduce diarrhea (including idiopathic, antibiotic-associated, *C. difficile*-associated), improve symptoms of irritable bowel disease, modulate the immune system, and inhibit pathogen colonization, among other benefits [1]. The underlying mechanisms of probiotic function rely on interactions between members of the gut microbiota (GM) and host cells. For example, incoming probiotic bacterial cells have been demonstrated to prevent colonization of pathogens by producing toxins, stimulating the immune system, or competitively excluding pathogens from the niche [1, 2].

Probiotics and their health benefits have also been demonstrated in dogs [3]. Dogs and humans suffer from some of the same GI disorders, and compositions of their GMs are similar [4–6]. Probiotics have improved symptoms in dogs suffering from diarrhea (chronic and acute), gastroenteritis, allergy, or other ailments [7–13], and reduced abundances of some pathogens, such as *C. difficile* [9]. However, some studies have reported conflicting data, demonstrating no effect on food-responsive chronic enteropathy [14], shelter-associated diarrhea [15], or acute idiopathic diarrhea [16]. Additionally, an improvement toward ideal body weight with probiotic supplementation has also been observed in underweight dogs [13, 17]. While clinical or biological outcomes were improved in these trials, changes in microbial composition were measured in only a limited number of studies [7, 13, 18–20]. Most of these included dogs with varied severity and types of diarrhea with a primary aim to improve diarrhea symptoms [7, 13, 20], and gut dysbiosis was shown to be improved with probiotic supplementation [7, 13].

The impacts of probiotics on GM measures and health maintenance in a generally healthy canine population remain largely unknown. The GI tract of healthy and diseased dogs likely provides different environments for bacterial growth and colonization [21]. Therefore, the investigation of probiotic supplementation in promoting and maintaining health throughout the life span is warranted. Of particular importance is whether probiotics can be used for prophylaxis in healthy dogs to prevent diarrhea incidences and other GI problems [22], and whether some healthy dogs may benefit more from supplementation than others based on baseline characteristics and microbiome composition. Moreover, the successful intestinal colonization of probiotic mixture has been reported in only certain breeds of healthy dogs [17–19]. Therefore, there is a need to expand the investigation to include a representative population of household dogs consisting of a heterogeneity of breeds and a range of ages.

Additionally, inulin is a common prebiotic found in dog food and supplements. Prebiotics are oligo- or poly-saccharides that stimulate the growth of beneficial bacteria in the gut. Inulin supplementation led to a significant decrease in *Coprobacillus* and a trend towards higher *Eubacterium* and *Turicibacter* in the gut of overweight dogs [23]. Likewise, when dogs fed a raw meat-based diet were given inulin, an increase of *Megamonas* and a decrease of *Fusobacterium* were observed [24].

In order to investigate the combined effect of probiotics and prebiotics on the GM profile in healthy dogs, a synbiotic (SN) supplement (a combination of probiotics and prebiotics) was formulated based on reviewing the existing probiotic literature to include bacterial species with demonstrated effects on various health outcomes, including GI health [8, 9, 25]. This high-dose formula contained some canine-derived probiotic strains as well as inulin as a prebiotic. Herein we describe the results from a randomized controlled trial examining the effects of this formula in healthy household dogs. GM was characterized by shotgun metagenomic sequencing, which provides superior taxonomic resolution as well as inferred function compared to the more commonly used 16S amplicon sequencing frequently used in microbiome research [26]. Differential responses to the supplement are also examined.

## Methods

### Animals and intervention

Fifty-one dogs of varied breeds were randomized to receive a daily dose of SN (*n* = 25) or placebo (PL, *n* = 26) for 4 weeks. Dog owners and technicians who processed the stool samples were blinded to the group assignment. Inclusion criteria included: aged 1–12 years, fed 2 meals per day of cooked diet (with treats < 10% of overall intake, guaranteed analysis of the diet available in Supplemental Table 1), absent of GI issues (including chronic diarrhea/vomiting or diarrhea/vomiting within 30 days prior to enrollment), absent of any infections or major chronic diseases (severe allergies, pancreatitis, diabetes, kidney disease or failure, liver disease, heart disease, cancer, severe GI issues when young [< 6 months old], surgery within the last 3 months prior to the enrollment), not pregnant or lactating at the time of enrollment, and limited to one dog per household. The absence of GI issues, infections, or major chronic diseases was evaluated and self-reported by owners using an online survey instrument prior to enrollment in the study. Dogs were not eligible to participate if they were fed any prebiotics, probiotics, cultured foods, or antibiotics within the last 3 months, or had a significant change in their diet within the last month prior to enrollment.

All owners were asked to collect stool samples using Nom Nom Plus Microbiome Testing Kits (NomNomNow, Inc., USA) and complete a comprehensive health survey at baseline and at 4 weeks (the end of the intervention period). Additional stool samples were collected at week 6 in the SN group (2 weeks after stopping the supplement). Each sample collection kit contained flocked fecal swabs and two collection vials. Dog owners were instructed to use a swab to collect two pea-sized samples, one for each vial. Each vial contained a preservation solution. The sample collection kits were used previously in a research study [6].

The PL consisted of maltodextrin. The SN used in the study was Nom Nom Plus Full Spectrum Probiotics for Dogs, using a daily dose of 2 g, consisting of 50 mg of inulin (derived from chicory root) and 20 billion live total CFU of a combination of the following species: *Lactobacillus reuteri* (canine-derived), *Pediococcus acidilactici* (canine-derived),* Enterococcus faecium* (canine-derived), *Lactobacillus acidophilus*, *Bifidobacterium animalis*, *Lactobacillus fermentum*, and *Lactobacillus rhamnosus*. These probiotic strains were chosen based on their individual benefits as previously reviewed [8, 9, 25]. All supplements used in this study were manufactured in the same batch and stored under the same conditions prior to delivering to recipients. Owners were instructed to mix one dose (2 g scoop) of the supplement into the dog’s meal once daily in the morning. Feeding instructions provided to dog owners were identical between the PL and SN groups. An email reminder was sent out to all owners once a week to ensure adherence to the baseline diet, supplement and medication usage, and exercise habits, as well as providing the opportunity to report any adverse effects that were observed during the study period.

### Health survey

Information on age, sex, body condition score (BCS), ideal body weight, physical activity level, neutered status, stool quality scale [27], and defecation and flatulence frequency was obtained through an online survey at baseline. Written descriptions and images were provided for the stool quality scale in the survey. At the end of week 4, dog owners completed a second set of questionnaires on overall health, physical activity, body weight, appetite, coat condition, stool quality scale, defecation and flatulence frequency, and incidence of diarrhea and/or vomiting.

### GM DNA extraction and library construction

All stool samples were processed and sequenced in a single batch at Diversigen, Inc., USA. DNA extraction and sequencing library construction protocols were performed as previously described with minor modifications [28]. Briefly, samples were extracted with Zymogen Quick-DNA Fecal/Soil Microbe 96 Mag Bead kit (Zymo Research, USA) using Powerbead Pro (Qiagen, USA) plates with 0.5 mm and 0.1 mm ceramic beads. Extraction controls included were a no template control (water) and a characterized homogenized stool. All samples were quantified with Quant-iT Picogreen dsDNA Assay (Invitrogen, USA). Libraries were prepared with a procedure adapted from the Nextera DNA Library Prep (Illumina, USA).

### GM shotgun metagenomic sequencing and annotation

Shotgun metagenomic sequencing was performed with BoosterShot™ (Shallow Sequencing, 2 M reads/sample) at Diversigen Inc., USA as previously described [28]. Library controls included a no template control (water) and DNA from a characterized homogenized stool. Libraries were sequenced on an Illumina NovaSeq using single-end 1 × 100 reads (Illumina, USA). For quality control, single end shotgun reads were trimmed and processed using Shi7 [29]. The sequences were then aligned to the NCBI RefSeq representative prokaryotic genome collection at 97% identity with BURST using default settings [30]. Taxa present in < 5% of the samples were removed. The resulting taxonomy table was also aggregated at higher taxonomic levels.

### GM functional annotation

Kyoto Encyclopedia of Genes and Genomes Orthology groups (KEGG KOs) were observed directly using alignment at 97% identity against a gene database derived from the genomic database used above. The KO table contained the directly observed KO counts within each sample. KO terms present in < 5% of the samples were removed as part of the quality filtering process.

### Mapping GM data to SN strains

Complete genome sequences were obtained for three of the SN bacterial strains (*L. reuteri*, *E. faecium* and *P. acidilactici*)*.* Reads from the stool samples were aligned against a database of contigs from these three strains at 97% identity with an e-value of 1e-10 using BLAST [31, 32].

To account for the four remaining genomes, shotgun metagenomic sequencing was performed on two samples of the SN using the same quality control and trimming steps as mentioned above (GM shotgun metagenomic sequencing and annotation section). These sequence reads from the SN were aligned against the database of contigs from completely sequenced genomes for three of the SN strains (*B. animalis*, *L. fermentum*, *L. acidophilus*). Reads that did not align against the database were used to create metagenome-assembled genomes (MAGs) contigs using MEGAHIT [33] with a minimum contig length of 1000. Gene calls were predicted from the MAG-generated contigs using Prodigal [34] and the MAG-generated contigs were grouped into bins using anvi’o [35]. Genome-level taxonomy of these bins was determined by aligning predicted single-copy core gene sequences assigned to the bins against the Genomic Taxonomy Database [36] using DIAMOND [37]. Stool sample reads were then aligned against a database of the MAG-generated contigs at 97% identity with an e-value of 1e-10 using BLAST.

Read counts (RC) of the mapped reads in samples collected at week 4 were compared between PL and SN subjects using generalized linear regression models.

### Statistical analysis

Continuous variables are expressed as mean ± standard deviation (SD), except for the fold change (FC) data which are expressed as mean ± standard error of the mean (SEM). Relative abundances of the bacteria are expressed as median (interquartile range). Categorical variables are presented as count (%). All analyses were performed using R Studio version 1.2.5033. Statistical significance was set at α = 0.05. Subject characteristics at baseline and health survey data at week 4 were compared between the SN and PL groups using two-sample t-test and Fisher’s exact test for continuous and categorical variables, respectively.

Species richness and Shannon’s diversity indices were computed by rarefying samples to various depths starting from 20,000 sequences per sample to a maximum depth of 380,000 sequences per sample and increasing depth by 20,000 reads. One hundred iterations were performed at each depth and the mean values were used as the estimate of these measures in each sample (Supplemental Figure 1). To investigate the effect of SN on α-diversity, the species evenness, richness, and Shannon’s as well as Simpson’s diversity indices were calculated at a sequencing coverage of 380,000 reads, listed in Supplemental Table 2. The Wilcoxon signed rank test was used to compare changes of alpha diversity metrics (evenness, richness, diversity indices) from week 0 to week 4 in each group, and Wilcoxon rank sum test was used to compare these metrics at each time point between SN and PL, as well as changes from week 0 to week 4 between SN and PL.

The non-rarefied count data were log-transformed and principal coordinate analysis (PCoA) was performed in R using the Bray-Curtis distance calculated with the vegan package at the species level [38]. Permutational multivariate analysis of variance (PERMANOVA) was performed using Bray-Curtis distance with 10,000 randomizations by including groups and timepoints to assess differences in community composition using the vegan package [38]. Differential abundance of bacterial taxa and KO terms between groups or timepoints was assessed at the species level using a negative binomial generalized linear model (GLM) using the differential expression analysis for sequence count data version 2 (DESeq2) package [39]. Taxa with absolute log_2_(FC) > 1 and adjusted *p*-values< 0.05 were considered significant. The Benjamini Hochberg method was performed to control the false discovery rate due to multiple comparisons.

## Results

### Subject characteristics

**Fig. 1:**
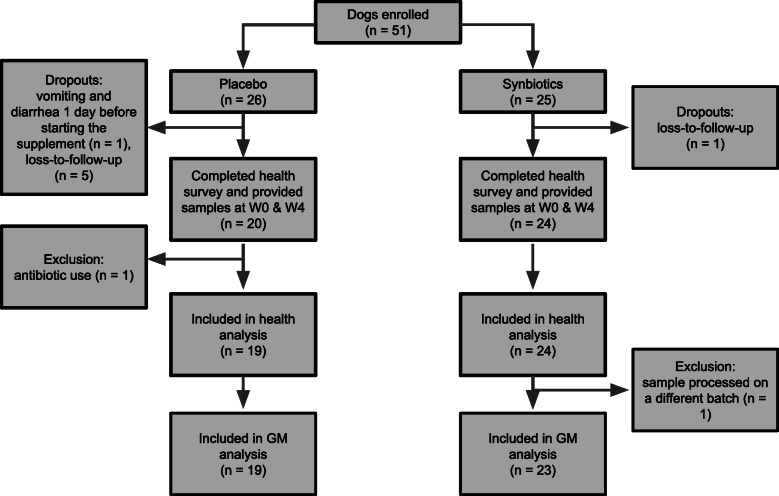
Trial flowchart

The owners of 24 dogs in the SN group and 20 dogs in the PL group provided the health assessment surveys and stool samples at weeks 0 and 4 (Fig. 1). Stool samples at week 6 from 21 dogs in the SN group were also available. Seven dogs did not complete the study for the following reasons: vomiting and diarrhea 1 day prior to starting the supplement (*n* = 1); lost-to-follow-up (*n* = 6). The dropout rates between the SN and PL were not statistically different (*p* = 0.10, Fisher exact test). One dog in the PL group was excluded from the analysis due to antibiotic use for bacterial dermatitis before completing the study. Characteristics of those included in the analysis are summarized in Table 1. Dogs were 5.6 ± 3.0 years old and 67% were male. Seventy-four percent had a BCS of 4–5 (ideal body condition) at the time of enrollment, while their ideal body weight was 11.5 ± 10.2 kg (reflecting the diversity in breeds). Data on breeds and diet are available in Supplemental Table 3. The two groups did not significantly differ in age, sex, BCS, ideal body weight, physical activity level, and neutered status. Fecal score was significantly lower (firmer stool) in the SN group at baseline (*p* = 0.010). Subjects missed taking the supplement 0.5 ± 0.9 and 0.9 ± 1.1 days in SN and PL, respectively, but none missed > 3 days during the 4-week study period.

**Table 1:** Subject characteristics at baseline

Feature	Synbiotics (***n*** = 24)	Placebo (***n*** = 19)	***p*** value*
Age, in years	5.0 ± 3.0	6.4 ± 3.0	0.134
Male	15 (63%)	14 (74%)	0.523
Spayed or neutered	21 (92%)	17 (89%)	1
Body condition score^a^			0.101
3	2 (8%)	0 (0%)	
4–5	20 (83%)	13 (68%)	
6	2 (8%)	5 (26%)	
7	0 (0%)	1 (5%)	
Ideal body weight (kg)	10.2 ± 7.8	11.6 ± 12.6	0.679
Physical activity level			0.273
Normal	16 (67%)	17 (89%)	
Active	5 (21%)	1 (5%)	
Very active	3 (13%)	1 (5%)	
Eating enthusiasm			0.854
High	19 (79%)	16 (84%)	
Normal	3 (13%)	1 (5%)	
Low	2 (8%)	2 (11%)	
Fecal score	2.6 ± 0.9	3.3 ± 0.9	0.010
Defecation frequency			0.419
≥ 3 times/day	5 (21%)	2 (11%)	
1–2 times/day	19 (79%)	16 (84%)	
< 1 time/day	0 (0%)	1 (5%)	

* Two-sample t-test for continuous variables and Fisher’s exact test for categorical variables

^a^ 9-point body condition score: 3 (mildly underweight), 4–5 (normal weight), 6 (mildly overweight), 7 (overweight)

### Comparisons of GM between SN and PL

#### GM diversity

A total of 107 samples were collected from 24 subjects in the SN group and 19 subjects in the PL group (3 samples/subject [week 0, 4, 6] for 21/24 SN dogs, 2 samples/subject [week 0, 4] for the remaining 3/24 SN dogs, and 2 samples/subject [week 0, 4] for 19 PL dogs). Two samples from one subject were processed on a different sequencing batch and excluded from subsequent GM analyses to eliminate any batch effect (Fig. 1).

A total of 8486 taxa were identified among all 105 samples, with an average sequencing depth of 1,538,444 ± 505,664 reads per sample, ranging from 389,644 to 2,903,240 reads (Supplemental Figure 2A). Nineteen taxa (accounting for 0.22% of all sequences) were removed from subsequent analyses because the taxon was present in < 5% of the samples (Supplementary Figure 2B). The removal of these taxa did not significantly change the average number of reads per sample or the range of sequencing coverage.

At a sequencing depth of 380,000 reads, α-diversity (measured using species evenness, richness, and Shannon’s and Simpson’s diversity indices) was not significantly different among the different time points in both the SN and the PL groups (Supplemental Table 2). Changes at week 4 from baseline for these measures were also not significantly different between the two groups.

**Fig. 2:**
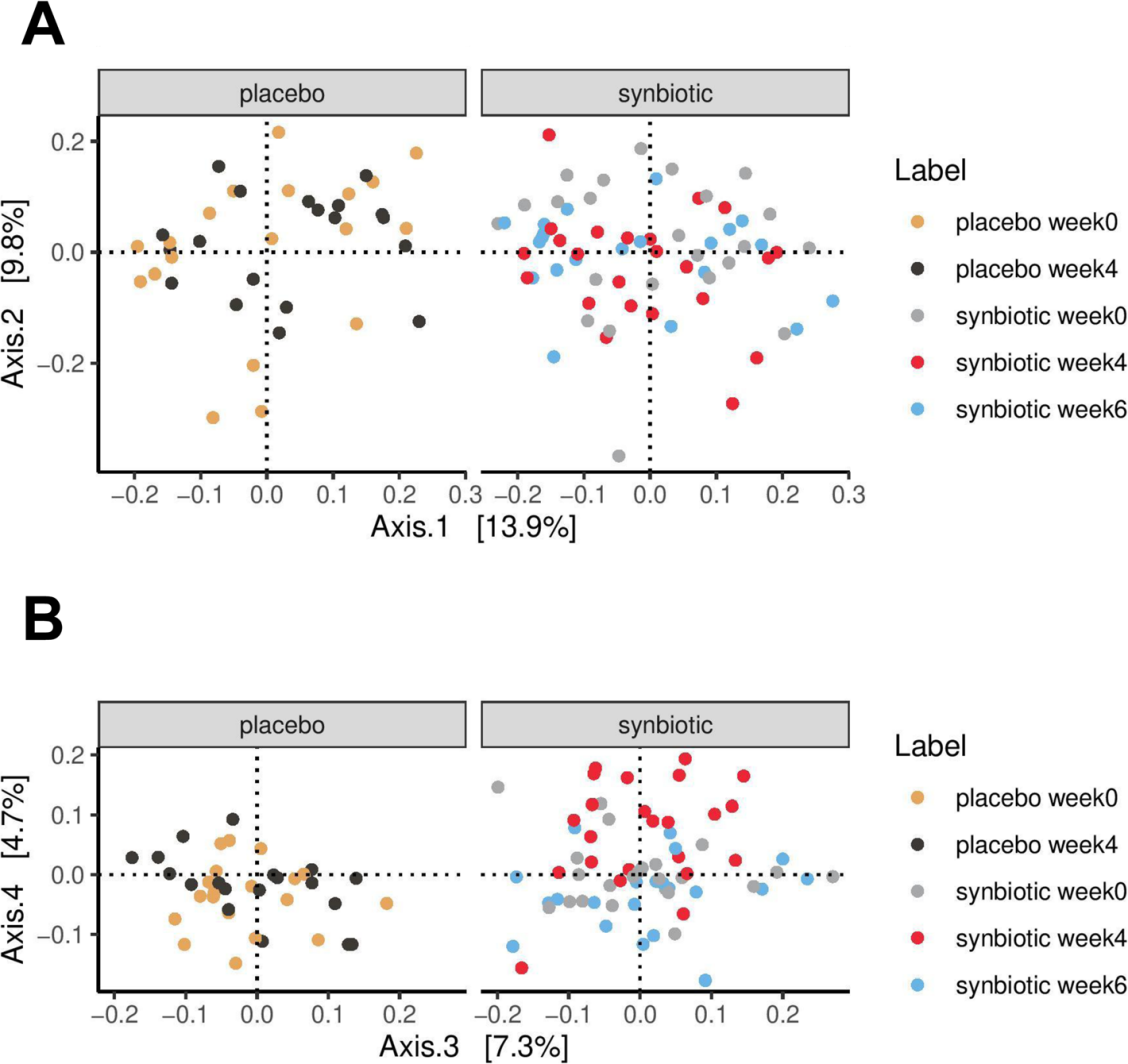
Principal coordinate analysis (PCoA) plot. **a** PCoA1 (Axis 1) and PCoA2 (Axis 2) respectively explained 13.9 and 9.8% of the variance of the abundance of gut microbiota at the species level (105 samples from 42 dogs). PERMANOVA using Bray-Curtis distance showed no spatial separation among groups (placebo and probiotics) or timepoints (weeks 0, 4, 6) based on PCoA1 and PCoA2 scores. **b** PCoA3 (Axis 3) and PCoA4 (Axis 4) respectively explained 7.3 and 4.7% of the variance of the abundance of gut microbiota at the species level

**Fig. 3:**
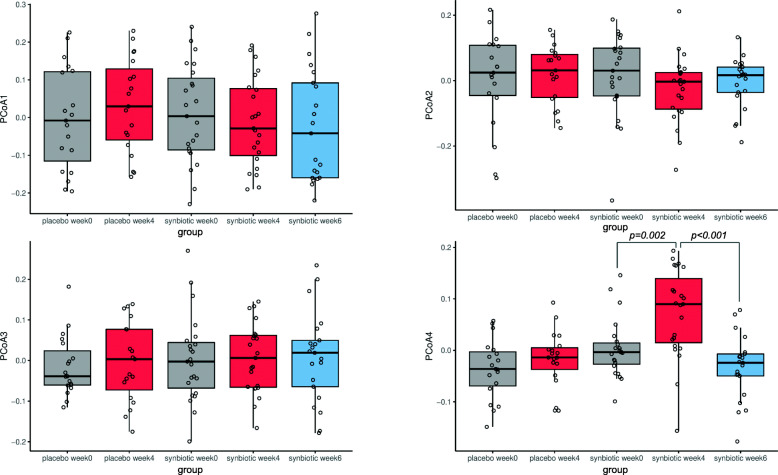
Scores of the first 4 PCoA axes in subjects receiving synbiotic (SN, *n* = 23) or placebo (PL, *n* = 19) at weeks 0, 4, and 6. PCoA4 score in the synbiotic group at week 4 was significantly different from that at week 0 (adjusted *p* = 0.002) and week 6 (adjusted *p* < 0.001). *P* value adjustment for pairwise comparisons was performed with the Benjamini Hochberg method

PCoA was used to investigate changes in β-diversity. The PCoA plot in Fig. 2a shows the first two principal coordinate axes (PCoA1 and PCoA2), which respectively explain 13.9 and 9.8% of the variation at the species level. Figure 2b shows the next two axes, with PCoA3 (7.3%), and PCoA4 (4.74%). The eigenvalues for the first 20 PCoA axes are displayed in Supplemental Figure 3. There were no differences in spatial separation among the supplementation groups or timepoints along the first two PCoA axes (*p* = 0.093, PERMANOVA using the Bray-Curtis distance matrices). Scores from the first three principal coordinates (PCoA 1–3) were not significantly different among timepoints within each group, or at each timepoint between groups (Fig. 3). PCoA 1–3 scores at baseline were also not associated with age, sex, neutered status, BCS, ideal weight, physical activity level, stool quality score, and defecation frequency across SN and PL groups (data not shown).

However, as shown in Fig. 3, scores of the fourth principal coordinate axis (PCoA4) in the SN group at week 4 (0.072 ± 0.087) were significantly different from PCoA4 scores at week 0 (0.003 ± 0.056, FDR-adjusted *p* = 0.002) and week 6 (− 0.033 ± 0.063, FDR-adjusted *p* < 0.001). This difference was not observed in the PL group, and not between week 0 and week 6 in the SN group. In other words, a shift in PCoA4 score, which explains 4.74% of the overall variation at the species level, was observed after 4 weeks of SN administration, and it returned to baseline 2 weeks after stopping the intervention. Further, the majority of the subjects (*n* = 20, 87%) in the SN group demonstrated a consistent shift in the same direction on the PCoA4 axis - increasing from week 0 to week 4 (also see high-, mid-, and low-responders section below). This proportion was significantly different than that in the PL group whose PCoA4 score increased in 11 dogs (58%) and decreased in 8 dogs (42%) between weeks 0 and 4 (*p* = 0.042, Fisher exact test), showing inconsistent shifts between the two supplementation groups. At week 4, PCoA4 score was also significantly higher in the SN group as compared to the PL group (SN: 0.072 ± 0.087 vs PL: − 0.018 ± 0.056, *p* < 0.001). Based on these results, it can be concluded that SN had a small but significant effect on the overall GM β-diversity.

#### GM relative abundances and composition

The phyla *Firmicutes* (SN: 69.2% [32.4–85.5%], PL: 57.9% [39.3–83.4%]), *Proteobacteria* (SN: 9.9% [1.6–41.8%], PL: 7.7% [0.5–48.8%]), *Bacteroidetes* (SN: 2.2% [0.2–11.9%], PL: 1.0% [0.09–7.3%]), and *Actinobacteria* (SN: 0.6% [0.09–10.9%], PL: 4.5% [0.3–19.1%]) constituted the majority of the gut bacteria in samples collected at baseline (Supplemental Figure 4).

**Fig. 4:**
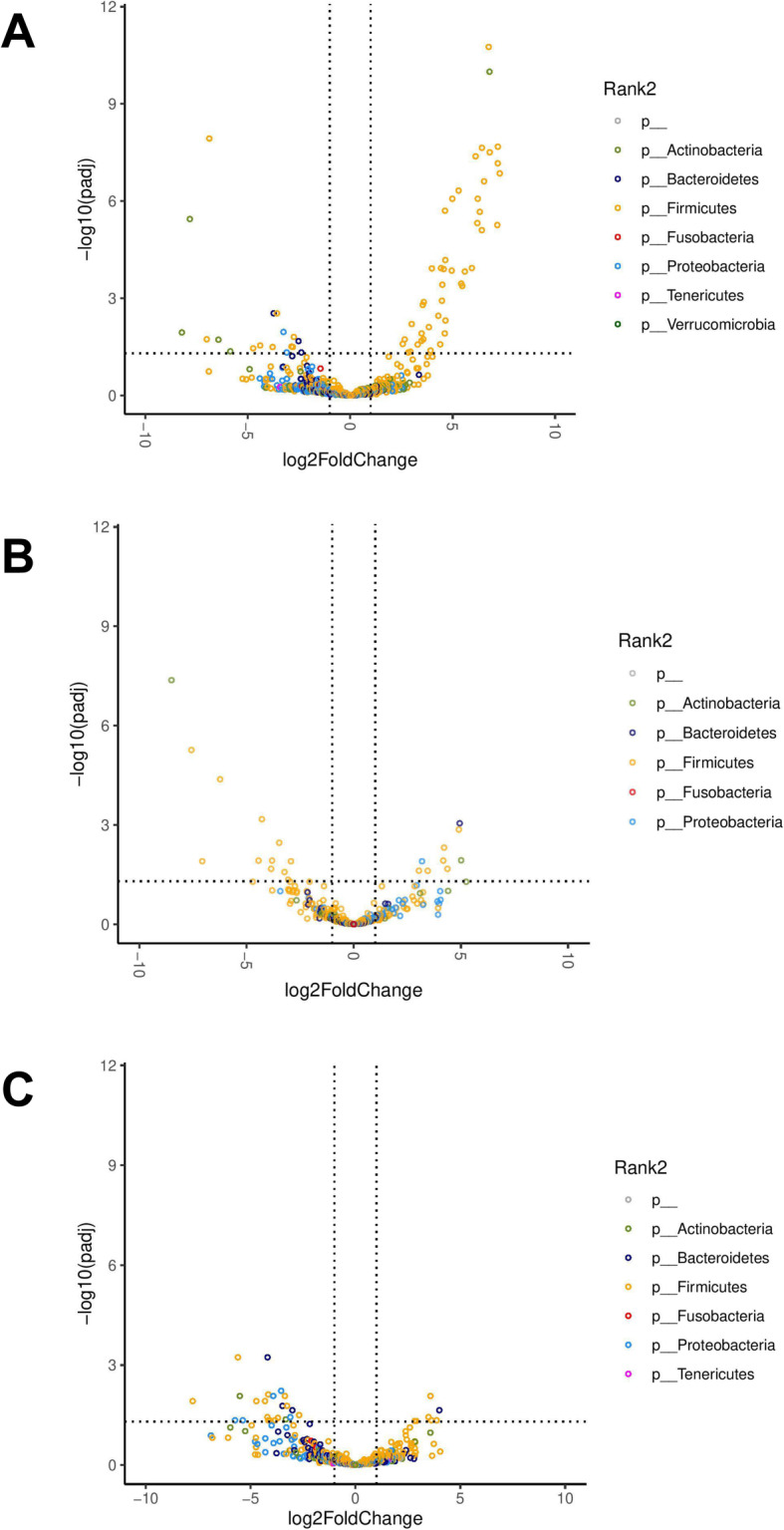
Volcano plots demonstrating the fold-change (FC) in the differential abundance analysis of gut bacteria at the species level (**a**) at week 4 compared to week 0 in the synbiotic group (*n* = 23); **b** at week 4 compared to week 0 in the placebo group (*n* = 19); and **c** at week 6 compared to week 0 in the synbiotic group (*n* = 21). Vertical dashed lines show log_2_FC at 1 and − 1 (i.e. FC at 2 and − 2). Horizontal dashed line shows -log_10_(adjusted p) = 2 (i.e. adjusted *p* = 0.01). Each point represents a different species and points are colored by phylum

GM relative abundances from samples collected at weeks 4 and 6 were compared with those collected at week 0 at the species level (Fig. 4 and Supplemental Figure 5). As listed in Supplemental Table 4A, the abundances of 45 species were shown to be significantly higher at week 4 as compared to week 0 in the SN group. All of the seven bacterial species present in the SN supplement were among these identified species: *L. reuteri* (log_2_FC = 6.43 ± 1.00, adjusted *p* < 0.001), *P. acidilactici* (log_2_FC = 6.53 ± 1.10, adjusted *p* < 0.001), *E. faecium* (log_2_FC = 3.00 ± 0.82, adjusted *p* < 0.001), *L. acidophilus* (log_2_FC = 6.76 ± 0.88, adjusted *p* < 0.001), *B. animalis* (log_2_FC = 6.80 ± 0.93, adjusted *p* < 0.001), *L. fermentum* (log_2_FC = 3.54 ± 0.88, adjusted *p* < 0.001), and *L. rhamnosus* (log_2_FC = 7.18 ± 1.35, adjusted *p* < 0.001). The abundances of these species returned to baseline at week 6, 2 weeks after stopping the SN, except for *L. acidophilus* whose abundance remained significantly higher than at baseline (log_2_FC = 3.48 ± 1.07, adjusted *p* = 0.037) (Supplemental Table 4C). No significant changes in the abundances of these species presented in SN were observed in the PL group between weeks 0 and 4 (Supplemental Table 4B).

Shotgun metagenomic sequencing of the SN supplement confirmed the presence of all seven SN species in the supplement. In order to confirm the observed increases in the SN strains in the fecal samples were from intake of the SN supplement, reads from stool samples were mapped to the strains in the supplement. Sequence contigs for complete genomes were obtained for *P. acidilactici, L. reuteri*, and *E. faecium.* MAG binning recovered three of the four the remaining SN strains (*L. acidophilus*, *B. animalis*, and *L. fermentum)*. As shown in Supplemental Figure 6, the RC in fecal samples mapped to the genomes of all six SN strains in the SN group was significantly higher than the PL group at week 4.

Increases in the abundances of additional species were also observed at week 4 in the SN group (Fig. 4a and Supplemental Figure 5A). They included 13 additional species of *Lactobacillus* (including *L. frumenti*, *L. vaginalis*, *L. plantarum*, *L. intestinalis*, *L. murinus*, *L. ingluviei*, *L. salivarius*, *L. taiwanensis*, *L. hominis*), and 22 species of *Enterococcus* (including *E. durans*, *E. villorum, E. pseudoavium*, *E. malodoratus*). The abundances of these bacteria did not increase at week 4 in the PL group and all returned to baseline at week 6 in the SN group. *Lactobacillus* and *Enterococcus* belong to the *Lactobacillales* order, which accounted for 96% of the species that showed a significant increase in the abundances at week 4. As shown in Supplemental Table 5, shotgun metagenomic sequencing of the SN supplement showed that most of these species were not detected or detected at very low abundances in the SN supplement, with exceptions of *L. plantarum* (3.15%) and unknown *Lactobacillus* (9.24%).

Moreover, the abundances of 15 known and 2 unknown species significantly decreased after 4 weeks of SN but not PL supplementation. They belonged to the genera *Clostridium*, *Arthrobacter*, *Kurthia*, *Lactobacillus*, *Timonella*, *Bacteroides*, *Lactococcus*, and *Streptococcus*. The abundances of these species returned to baseline at week 6 (Fig. 4c and Supplemental Figure 5C), except for *Kurthia sp. Dielmo* which remained statistically lower at week 6 (log_2_FC = − 7.76 ± 2.07, adjusted *p* = 0.012). The abundance of an unknown species of *Arthrobacter* also decreased at week 4 (log_2_FC = − 7.83 ± 1.45, adjusted *p* < 0.001) and remained lower than baseline at week 6 (log_2_FC = − 5.52 ± 1.42, adjusted *p* < 0.001).

Changes in the abundances of 21 species of GM were also observed at week 4 in dogs receiving PL (Fig. 4b and Supplemental Figure 5B). These 21 identified species did not overlap with the identified species in the SN group with the single exception of *L. pisicum* which decreased at week 4 in both the SN and PL groups. Most of these 21 species were in the *Latobacillales* order, except for *P. freudenreichii*, *P. copri*, *C. freundii*, *C. perfringens*, and *B. animalis*.

#### GM functional data

A total of 4651 KO terms in 105 samples were identified, and after filtering, 3615 remained for subsequent analyses. In the PL group, one KO term showed a significant increase while one KO term showed a significant decrease in abundance at week 4 compared to baseline. On the other hand, a significant increase in the abundance of 15 KO terms was observed in the SN group after 4 weeks as listed in Table 2. These KOs are associated with multiple metabolic pathways including: aromatic compound degradation (K18364), biosynthesis of macrolides (K16001), starch and glucose metabolism (K16147, K00689), pentose phosphate pathway (K01621), and lipoic and propionic acid metabolism (K16869, K01699). Additionally, a significant decrease in 2 KO terms (K11521, K11384) was observed. Significant changes were no longer observed at week 6.

**Table 2:** Kyoto Encyclopedia of Genes and Genomes Orthology (KO) terms with significant increase or decrease in the differential abundance analysis (|fold change| ≥ 2 and adjusted *p* < 0.05) at week 4 or 6 relative to week 0

KO terms	Description	Synbiotics Week 4 vs Week 0 (***n*** = 23)		Placebo Week 4 vs Week 0 (***n*** = 19)		Synbiotics Week 6 vs Week 0 (***n*** = 21)	
		Log 2 FC* Mean ± SE	Adjusted p**	Log 2 FC* Mean ± SE	Adjusted p**	Log 2 FC* Mean ± SE	Adjusted p**
K18364	2-oxopent-4-enoate/cis-2-oxohex-4-enoate hydratase	6.69 ± 0.91	8.30E-10	–	NS	–	NS
K16001	narbonolide/10-deoxymethynolide synthase	5.03 ± 0.87	1.23E-05	–	NS	–	NS
K16147	starch synthase (maltosyl-transferring)	4.11 ± 0.98	1.14E-02	–	NS	–	NS
K00689	dextransucrase	4.06 ± 0.89	3.91E-03	–	NS	–	NS
K01621	xylulose-5-phosphate/fructose-6-phosphate phosphoketolase	3.94 ± 0.89	5.22E-03	–	NS	–	NS
K16323	purine nucleoside transport protein	3.76 ± 0.85	5.22E-03	–	NS	–	NS
K16869	octanoyl-[GcvH]:protein N-octanoyltransferase	3.66 ± 0.75	1.20E-03	–	NS	–	NS
K20510	malonyl-S-ACP:biotin-protein carboxyltransferase subunit MadC	3.62 ± 0.76	1.70E-03	–	NS	–	NS
K20708	isoleucine 2-epimerase	3.61 ± 0.87	1.27E-02	–	NS	–	NS
K03378	morphine 6-dehydrogenase	3.49 ± 0.79	5.22E-03	–	NS	–	NS
K13929	malonate decarboxylase alpha subunit	3.07 ± 0.75	1.46E-02	–	NS	–	NS
K01699	propanediol dehydratase large subunit	2.66 ± 0.67	2.09E-02	–	NS	–	NS
K10556	AI-2 transport system permease protein	1.80 ± 0.48	3.85E-02	–	NS	–	NS
K06042	precorrin-8X/cobalt-precorrin-8 methylmutase	1.69 ± 0.46	4.83E-02	–	NS	–	NS
K00841	aminotransferase	1.23 ± 0.31	2.08E-02	–	NS	–	NS
K15521	D-inositol-3-phosphate glycosyltransferase	−2.13 ± 0.57	4.31E-02	–	NS	–	NS
K11384	two-component system, NtrC family, response regulator AlgB	−1.87 ± 0.51	4.67E-02	–	NS	–	NS
K15629	CYP152A; fatty-acid peroxygenase [EC:1.11.2.4]	–	NS	–	NS	5.10 ± 1.14	7.41E-06
K16139	glucuronide carrier protein	–	NS	–	NS	−4.62 ± 1.07	2.76E-02

*FC* fold change, *NS* not significant

* Log_2_FC > 1 represents 2 fold-change at week 4 or 6 compared to week 0

** *P* values were adjusted using the false discovery rate

### Heterogeneous response to SN

#### High-, mid-, and low-responders

**Fig. 5:**
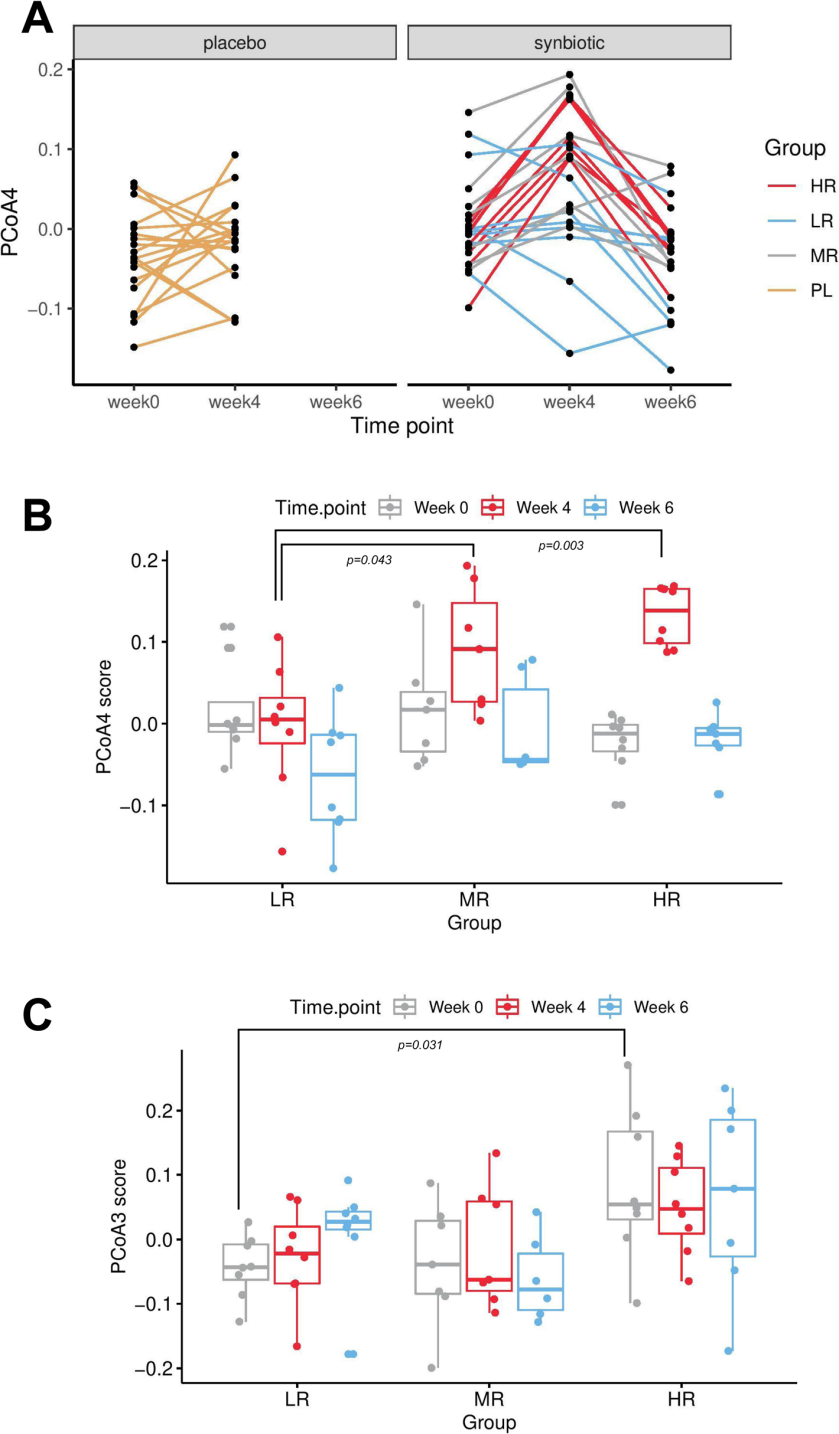
Varying degrees of PCoA4 changes from week 0 to week 4 were observed among subjects receiving the synbiotic supplement. **a** PCoA4 score at week 4 increased in 20 dogs (87%) as compared to baseline in the synbiotic group, while the direction of change was less consistent in the placebo (PL) group - increased in 11 dogs (58%) and decreased in 8 dogs (42%). Subjects in the synbiotic group were divided into tertiles based on the degree of PCoA4 changes between week 0 and week 4, with subjects in the first tertile labeled high-responders (HR, maximal PCoA4 score increase, *n* = 8), those in the second tertile mid-responders (MR, *n* = 7), and those in the third tertile low-responders (LR, PCoA4 score decrease or minimal PCoA4 score increase, *n* = 8). **b** PCoA4 scores at week 4 were significantly higher in HR (0.132 ± 0.037, FDR-adjusted *p* = 0.003, pairwise Wilcoxon rank sum test) and MR (0.091 ± 0.076, FDR-adjusted *p* = 0.043) as compared to LR (−0.004 ± 0.080). **c** PCoA3 scores were significantly higher in HR (0.084 ± 0.117) than LR (−0.043 ± 0.049) but not MR (− 0.038 ± 0.096) at baseline (FDR-adjusted *p* = 0.031, Kruskal-Wallis test). PCoA3 scores were not significantly different among groups at week 4 or week 6

As demonstrated in Fig. 5a, while an average effect of SN on GM composition was observed at week 4, the magnitude of the response varied among individual subjects within the SN group. In order to investigate this heterogeneity of response to SN, the subjects were divided into tertiles based on the degree of PCoA4 change from week 0 to week 4, with subjects in the first tertile labeled high-responders (HR, maximal PCoA4 score increase, *n* = 8), those in the second tertile labeled mid-responders (MR, *n* = 7), and those in the final tertile labeled low-responders (LR, PCoA4 score decrease or minimal PCoA4 score increase, *n* = 8).

As seen in Fig. 5b, PCoA4 scores were not significantly different among HR, MR, and LR at baseline (*p* = 0.397, Kruskal-Wallis test) or week 6 (*p* = 0.367). However, they were significantly higher at week 4 in HR (0.132 ± 0.037, FDR-adjusted *p* = 0.003, Kruskal-Wallis test) compared to LR (− 0.004 ± 0.080), and MR (0.091 ± 0.076, FDR-adjusted *p* = 0.043) compared to LR, but not between MR and HR (FDR-adjusted *p* = 0.536). While PCoA1, 2, and 4 scores did not significantly differ among HR, MR, and LR at baseline, PCoA3 scores were significantly higher in HR (0.084 ± 0.117) than LR (− 0.043 ± 0.049) but not MR (− 0.038 ± 0.096) (FDR-adjusted *p* = 0.031, Kruskal-Wallis test, Fig. 5c). Additional differences in GM abundances are reported in the following section.

Subject characteristics at baseline were further compared between HR, MR, and LR, and were not statistically different (Supplemental Table 6). GM α-diversity metrics including evenness, Shannon’s and Simpson’s diversity indices were significantly lower in HR and LR compared to MR at baseline (*p* = 0.022 for all metrics, Kruskal-Wallis test), but not at weeks 4 and 6 (Supplemental Table 7 and Supplemental Figure 7). Changes in these metrics from week 0 to week 4 or 6 were not different across the among groups.

#### HR and LR exhibited different GM at baseline and 4 weeks after SN supplementation

**Fig. 6:**
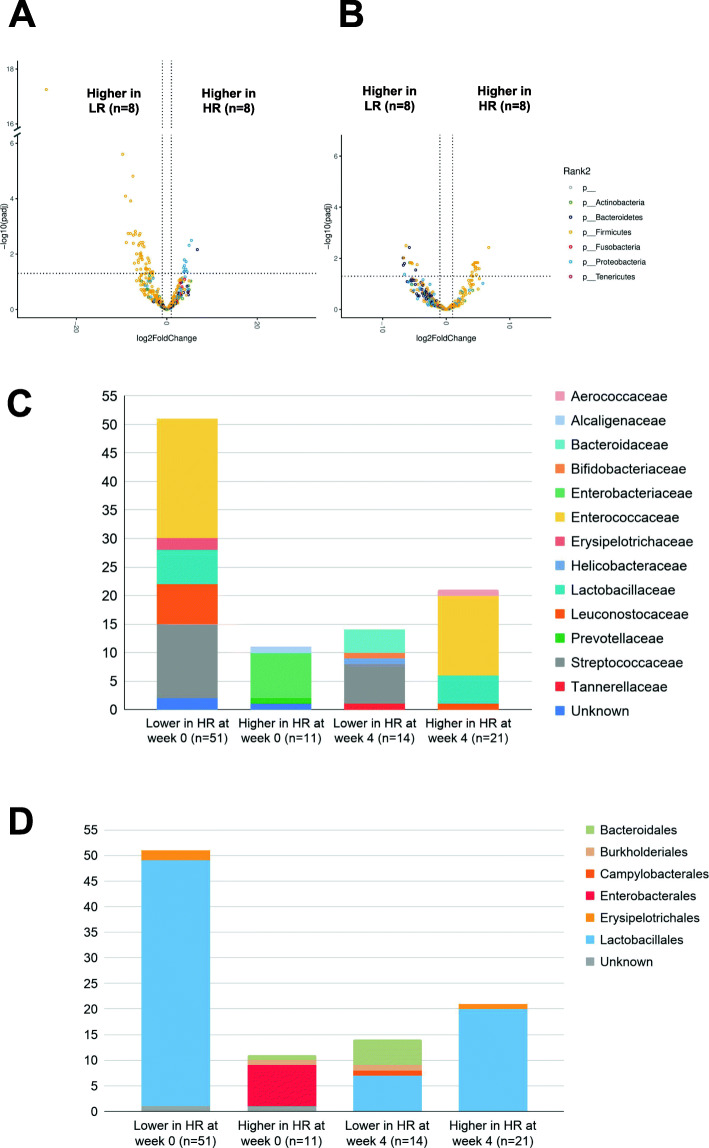
Volcano plots demonstrating fold-change (FC) in the differential abundance analysis of gut bacteria between HR (high-responders, *n* = 8) and LR (low-responders, *n* = 8) among dogs receiving the probiotics at **a** week 0 and **b** week 4. Each point represents a different species and points are colored by phylum. Numbers of taxa with significantly different abundances are shown at **c** the family level and **d** the order level

Significant differences in the abundances of 62 species of gut bacteria between HR and LR were observed at baseline, of which 51 species and 11 species were lower and higher in HR, respectively (Fig. 6a, Supplemental Figure 8A, Supplemental Table 8A). Among these species that were underrepresented in HR, 21 (41%) were *Enterococcaceae*, 13 (25%) *Streptococcaceae*, 7 (14%) *Leuconostocaceae*, and 6 (12%) *Lactobacillaceae* family (Fig. 6c). At the order level, all but three species (94%) were *Lactobacillales* (Fig. 6d and Supplemental Figure 8A). For those 11 species whose abundances were significantly higher in HR, 9 (82%) species, including *Escherichia coli* and *Escherichia albertii*, were in the *Enterobacteriaceae* family, which belongs to the *Enterobacterales* order. The other two species were *Achromobacter* and one unknown species that belonged to the *Gammaproteobacteria* class. This observation likely reflected the difference in PCoA3 scores between HR and LR at baseline (see High-, mid-, and low-responders section). None of the abundances of seven SN species differed between the guts of HR and LR at baseline.

After 4 weeks of SN supplementation, the abundance of 53 species significantly increased in HR, 51 (96%) of which belong to the order *Lactobacillales* (Supplemental Table 9A), while no significant increase of any species was observed in LR (Supplemental Table 9B). Of these 53 species, twenty one species were identified as having significantly higher abundances in HR as compared to LR at week 4 (Fig. 6b, Supplemental Figure 8B, Supplemental Table 8B). None of these species were overrepresented in HR at baseline. Among these 21 species, 14 (67%) belonged to the *Enterococcaceae* family and 5 (24%) belonged to the *Lactobacillaceae* family (Fig. 6c). At the order level, all but one species (95%) were *Lactobacillales*, similar to those species in the *Lactobacillales* order that were present in low levels in HR at baseline (Fig. 6d and Supplemental Figure 8B). *L. reuteri* (log_2_FC = 4.84 ± 1.39, adjusted *p* = 0.015) and *E. faecium* (log_2_FC = 4.19 ± 1.25, adjusted *p* = 0.018), both of which were in the SN supplement, were among these 21 identified species. The species in the *Enterobacteriaceae* family which had been overrepresented in HR at baseline were no longer overrepresented in HR at week 4. This was further supported by the observation that PCoA3 scores were no longer different among HR, MR, and LR at week 4 (Fig. 5c). Conversely, 14 species were observed to have lower abundances among HR at week 4, 7 (50%) and 4 (29%) of which belonged to the *Streptococcaceae* family and the *Bacteroidaceae* family, respectively. At the order level, 7 (50%) were *Lactobacillales* and 5 (36%) were *Bacteroidales*. At week 6 (2 weeks after stopping the SN supplementation), abundances of all species did not significantly differ between HR and LR.

Likewise, differences at the species level were observed when MR was compared with HR or LR. At baseline, 18 species were differentially present between HR vs MR, and 48 species were differentially present between MR vs LR (Supplemental Table 10). At week 4, 13 species were differentially present between HR vs MR, and 13 species were differentially present between MR vs LR (Supplemental Table 11). As shown in the tables, many of the identified species were consistent with the above-mentioned results when HR and LR were compared.

The average relative abundance of GM in HR and LR at the family and order levels are also shown in Supplemental Figure 9. The figure reflects the changes at the species level previously described.

### Health outcomes

No health outcomes and adverse effects were statistically different between the SN and PL groups at the end of the intervention at week 4 (Table 3). While 16% of dogs in the PL group had an incidence of diarrhea during the 4-week period, none of the dogs in the SN group experienced diarrhea. After starting the supplement, vomiting was reported in 4 dogs (21%) in the PL group and 1 dog (4%) in the SN group during the 4-week study period. Although both the diarrhea and vomiting incidences were reduced in the SN group, neither reached statistical significance (*p* = 0.08 for diarrhea, Fisher’s exact test). Other reported adverse effects included constipation (*n* = 1) in the SN group and itching (*n* = 1) in the PL group.

**Table 3:** Health outcomes after the supplementation at week 4 compared to baseline

Feature	Synbiotics (***n*** = 24)	Placebo (***n*** = 19)	***p*** value*
Overall health			0.717
Better	1 (4%)	1 (5%)	
No effect	23 (96%)	17 (89%)	
Worse	0 (0%)	1 (5%)	
Physical activity			1
More active	2 (8%)	2 (11%)	
No effect	22 (92%)	17 (89%)	
Less active	0 (0%)	0 (0%)	
Body weight			0.217
Improved	0 (0%)	2 (11%)	
No effect	23 (96%)	15 (79%)	
Worsened	1 (4%)	2 (11%)	
Appetite			0.111
Increased	7 (29%)	1 (5%)	
No effect	15 (63%)	14 (74%)	
Reduced	2 (8%)	4 (21%)	
Coat condition			0.855
Improved	4 (17%)	4 (21%)	
No effect	19 (79%)	14 (74%)	
Worsened	1 (4%)	1 (5%)	
Bristol stool score	2.5 ± 0.9	3.0 ± 1.0	0.095
Defecation frequency			0.405
Increased	5 (21%)	1(5%)	
No effect	15 (63%)	15 (79%)	
Decreased	4 (17%)	3 (16%)	
Flatulence frequency			0.529
Increased	1 (4%)	1 (5%)	
No effect	22 (91%)	15 (79%)	
Decreased	1 (4%)	3 (16%)	
Diarrhea incidence			0.078
Yes	0 (0%)	3 (16%)	
No	24 (100%)	16 (84%)	
Vomiting incidence			0.306
Yes	1 (4%)	3 (16%)	
No	23 (96%)	16 (84%)	

*Two-sample t-test for continuous variables and Fisher’s exact test for categorical variables

## Discussion

The primary goal of this randomized controlled trial was to identify the effects of a multi-species SN on the gut microbial community composition and function in healthy dogs. A limited number of studies have investigated the effect of probiotic administration on the fecal microbiota in healthy dogs, but include only Alaskan Husky sled dogs, Beagles, and Boxers [17–19]. The present study was a defined cohort of household dogs representative of pet dogs in the United States. The cohort comprised a diversity of ages and breeds, included both males and females, and had a relatively large sample size. Therefore, these findings should be more applicable to general dog populations than studies performed in laboratory or working dogs. Moreover, while a probiotic consisting of a single strain of bacteria was chosen as the supplementation in many studies [17, 40–47], the present study adopted a SN supplement consisting of inulin and seven bacterial species, three of which have canine origin. Finally, the use of shotgun metagenomic sequencing, which has not been widely used in the characterization of the canine microbiota [7, 48–50], increases taxonomic resolution and therefore detection of bacterial species and diversity, and is superior in functional prediction compared to the more commonly used 16S amplicon sequencing [26].

A variety of changes in bacterial species abundances were observed during the 4 week trial period in both the SN and PL groups. The observed differences in the PL group highlight normal short-term fluctuation of the GM of healthy dogs even in the absence of meaningful interventions. However, the greatest number and significance of differences were observed in the SN group. These differences were also reflected in changes of β-diversity along the PCoA4 axis solely in the SN group, indicating that these changes were most likely caused by the supplement. SN also led to increased abundances of the bacteria present in the supplement itself, which included species of *Lactobacillus*, *Enterococcus*, *Bifidobacterium*, and *Pediococcus*. To further examine this, we mapped the samples collected at week 4 against genomic sequences or MAGs generated from metagenomic sequencing of the supplement, and demonstrated significant alignment. The high abundances of DNA from these probiotic bacteria in feces indicate that these bacteria likely survived gastric transit. These probiotic strains have demonstrated health benefits such as ameliorating symptoms in idiopathic inflammatory bowel disease and acute or intermittent diarrhea, improving stool quality, and improving the intestinal barrier integrity in dogs [7, 51, 52].

Dogs given the supplement also had decreased abundances of three species of *Clostridium* that are rare potential pathogens (*C. celatum*, *C. baratii*, and *C. disporicum*) [53–55] and several species with evidence of opportunistic pathogenicity (e.g., *Citrobacter* spp. and *Bacteroides* spp.) [56–59]. However, the pathogenicity of these *Clostridium*, *Citrobacter*, and *Bacteroides* species has been documented only in humans. These results are consistent with probiotic bacteria reducing pathogen load of the intestines by preventing pathogen colonization, particularly in the case of *Clostridium* species [60, 61].

The synbiotic in this study was formulated to include bacterial species with demonstrated effects on various health outcomes, including GI health [8, 9, 25]. Given that inulin was given together with probiotics, its isolated prebiotic effect cannot be determined in this study. Previously, inulin supplementation led to a decrease in *Coprobacillus* and *Fusobacterium*, and an increase in *Eubacterium*, *Turicibacter*, and *Megamonas* [23, 24]. We did not observe changes in the abundances of these genera in our study (Fig. 6a and Supplemental Figure 5A). Therefore, it is suggested that the prebiotic effect of inulin may vary depending on diet, other supplements, and pre-intervention microbiota composition.

As demonstrated by the few changes in the abundance of the KO terms, overall GM function did not differ drastically before and after SN supplementation. None of the identified KO terms have been previously reported to have any associations with canine health. This observation likely reflects the relatively small, albeit significant, observed compositional changes in healthy dogs, as well as no reported overall change in health outcomes. Further investigation incorporating other -omics approaches such as transcriptomics and metabolomics may provide better insights into direct or indirect synbiotic functionality.

In dogs given the SN, changes in the β-diversity (PCoA4) returned to baseline after the supplement use was discontinued for 2 weeks. The abundances of six out of the seven probiotic species also returned to baseline. The one exception was *L. acidophilus*, whose abundance in feces remained high for at least 2 weeks after probiotic use was discontinued, indicating that *L. acidophilus* may have more permanently engrafted in the gut. Otherwise this observation indicates that the probiotic bacteria did not permanently colonize the GI tract at detectable levels. In contrast, the abundances of two species, *Kurthia sp. Dielmo* and an unknown *Arthrobacter* sp., did not return to their baseline levels after being reduced by SN use. Little is known about *Kurthia sp. Dielmo*, and *Arthrobacter* species are generally found in soil, but have been isolated from oral cavities of healthy dogs [62–64]. With the exception of these examples, our findings corroborated previous findings that GM changes occur in a relatively short period of time (within 6–15 days) after stopping supplementation [13, 19, 41, 46, 47]. They also support that engraftment of probiotics is generally uncommon and not required for their beneficial effects [65], and that continued administration of probiotics or synbiotics may be necessary to produce prolonged effects.

Changes in community composition after the SN supplementation were most striking in a subset of the dogs, termed “high-responders” or HR. The abundances of 53 species increased in the HR, 96% of which belonged to the order *Lactobacillales*. These changes were no longer evident 2 weeks after SN use was discontinued. Interestingly, changes in the abundances of these 53 species were not observed among the “low-responders” or LR despite their receiving the identical supplementation throughout the 4 weeks. It should be noted that the level of “response” reported in this study refers solely to the magnitude of changes in the GM composition (β-diversity), which may or may not be associated with clinical responses given the lack of clinical data.

This raises the question of what factors contribute to such differential responses in this cohort of healthy dogs. We identified that baseline GM composition of HR versus LR significantly differed in β-diversity (PCoA3) and the abundances of some bacterial species. These differences may be the reason certain dogs were more responsive to the effects of SN supplementation than others. Specifically, species belonging to the *Lactobacillales* order were underrepresented in HR prior to supplementation. One possibility is that the *Lactobacillus* niche in LR is already being utilized by other lactic-acid bacteria, therefore they were more resistant to any change induced by the supplement. Baseline compositional differences that result in varying magnitudes of response to supplementation are increasingly common in the literature. With regard to probiotic supplementation, this observation is consistent with studies in human subjects that have identified certain individuals whose gut microbial communities respond to probiotics whereas the communities in other individuals exhibit lower or no responses [66, 67]. To our knowledge, this is the first study documenting such an effect in companion dogs. Further, it appears as though the GM community in the MR, whose α-diversity was higher than HR and LR at baseline, became less diverse with the supplementation, even though the shift was not significant (Supplemental Figure 7). This is interesting as it has been previously observed that communities with higher compositional diversity may be less likely to respond to probiotic interventions [68]. Rather than a one-size-fits-all regimen, the results from our study support the notion that the prediction of response to SN supplementation and tailored probiotic recommendation may be possible based on their identified baseline microbiota [66].

It should be noted that baseline GM composition may be influenced by a myriad of factors. Even though age, sex, and BCS were not significantly different among HR, MR, and LR (Supplemental Table 3), this study was not designed to have a statistical power to detect such possible associations. All dogs were on a nutritionally balanced mildly cooked diet from the same manufacturer, and therefore the diet was controlled to a certain extent. However, the variety of recipe combinations does introduce some uncertainty in understanding the role of diet on GM compositional change (Supplemental Table 3).

In the HR, many of the bacteria whose abundances were significantly reduced by the SN are known have been associated with GI diseases in dogs [69], including species of *Shigella* and *Escherichia* [70, 71]. These dogs did not have reported clinical symptoms of GI diseases, so the clinical impact of this change by SN is unknown. It is intriguing to speculate that the presence of these pathogens may indicate increased susceptibility or predisposition to developing GI disorders or diseases, and that SN supplementation may have a prophylactic effect by reducing their abundances as previously reported [72–74]. Consistent with this was our finding that the PL group had a non-significant trend toward higher incidence of diarrhea than SN.

## Conclusion

SN administration for 4 weeks caused a small but significant shift in the GM profile and predicted function in healthy dogs. The shift included an increase in the abundance of bacteria contained in the SN and a decrease in potentially pathogenic bacteria, and GM composition largely returned to baseline 2 weeks after the termination of SN supplementation. Heterogeneity of response to supplementation was observed. Dogs whose GM at baseline included low levels of *Lactobacillales* and high levels of several pathogens, despite having no clinical symptoms, responded to the SN supplementation to a greater extent by reducing pathogen load and increasing abundances of beneficial lactic-acid bacteria, among others. Future trials with longer duration in healthy dogs or in a population at higher risk of diarrhea are respectively warranted to investigate the possible effect of SN supplementation on health maintenance and disease prophylaxis.

## Supplementary Information

**Additional file 1: Figure S1**. Rarefaction curves demonstrate sequencing coverage used to calculate species (A) richness and (B) Shannon’s diversity index in subjects receiving synbiotic (*n* = 23) or placebo (*n* = 19). Species richness was calculated from 10,000 to 380,000 reads. Each point represents a mean and each error bar represents a standard deviation at each rarefaction depth. **Figure S2**. Shotgun metagenomic sequencing data quality control. Plots show the abundance (x-axis, as count) and the prevalence (y-axis, as percentage of all samples) of each read for all phyla (A) pre- and (B) post-filtering. Each data point represents a read. As a part of the filtering process, 19 taxa from phyla *Candidatus Kryptonia*, *Candidatus Saccharibacteria*, *Chloroflexi*, *Deinococcus-Thermus*, *Gemmatimonadetes*, *Nitrospirae*, *Planctomycetes*, *Synergistetes* were removed because each represented < 5% of samples or belonged to an unknown phylum. **Figure S3**. Scree plot showing eigenvalues of the first 20 principal coordinate axes. **Figure S4**. Relative abundance at the phylum level in the samples collected at different time points in dogs receiving placebo or synbiotic. **Figure S5**. Dot plots demonstrating the fold-change (FC) in the differential abundance analysis of gut bacteria at the species level (A) at week 4 compared to week 0 in the synbiotic group (*n* = 23); (B) at week 4 compared to week 0 in the placebo group (*n* = 19); and (C) at week 6 compared to week 0 in the synbiotic group (*n* = 21). Only species that were significantly different are shown (significance was determined by a negative binomial generalized linear model [GLM] using the differential expression analysis for sequence count data version 2 [DESeq2] package with log_2_FC at 1 and − 1 [i.e. FC at 2 and − 2] and -log_10_(adjusted p) = 2 [i.e. adjusted *p* = 0.01]). Each point represents a different species and points are colored by order. **Figure S6**. Box plots displaying the number of read counts (RC) in fecal samples collected at week 4 mapped to six strains in the synbiotic supplement. Comparison was performed using the general linear regression model and differences were significant in each species: *L. reuteri* (Δ [log_10_RC] = 1.68, adjusted *p* = 3.93e-09), *P. acidilactici* (Δ [log_10_RC] = 0.41, adjusted *p* = 7.10e-03), *E. faecium* (Δ [log_10_RC] = 1.17, adjusted *p* = 4.94e-07), *L. acidophilus* (Δ [log_10_RC] = 1.66, adjusted *p* = 1.37e-05), *B. animalis* (Δ [log_10_RC] = 1.24, adjusted *p* = 2.49e-05), and *L. fermentum* (Δ [log_10_RC] = 0.43, adjusted *p* = 0.023). **Figure S7**. (A) Evenness, (B) richness, (C) Shannon’s and (D) Simpson’s diversity indices of the gut bacteria among HR (high-responders, *n* = 8), MR (mid-responders, *n* = 7), and LR (low-responders, *n* = 8), at different time points. **Figure S8**. Dot plots demonstrating the fold-change (FC) in the differential abundance analysis of gut bacteria between HR (high-responders, *n* = 8) and LR (low-responders, *n* = 8) among dogs receiving the probiotics at (A) week 0 and (B) week 4. Only species that were significantly different are shown (significance was determined by a negative binomial generalized linear model [GLM] using the differential expression analysis for sequence count data version 2 [DESeq2] package with log_2_FC at 1 and − 1 [i.e. FC at 2 and − 2] and -log_10_(adjusted p) = 2 [i.e. adjusted *p* = 0.01]). Each point represents a different species and points are colored by order. **Figure S9**. Average relative abundance in high-responders (HR, *n* = 8) and low-responders (LR, *n* = 8) at week 0 and week 4 at the (A) family and (B) order levels. The legend shows only those whose relative abundance > 1% at any time point.

**Additional file 2: Table S1**. Guaranteed analysis and ingredients of the 4 cooked diet recipes.

**Additional file 3: Table S2**. Measures of gut microbial evenness and α-diversity at weeks 0, 4, and 6 at sequencing coverage 380,000 reads.

**Additional file 4: Table S3**. Breeds and diet of study participants (placebo *n* = 19, synbiotic *n* = 24).

**Additional file 5: Table S4A**. Species of gut bacteria with significant increase or decrease in the differential abundance analysis (|fold change| ≥ 2 and adjusted *p* < 0.05) at week 4 relative to week 0 in the synbiotic group (*n* = 23). Species in bold were present in the synbiotic supplement. **Table S4B**. Species of gut bacteria with significant increase or decrease in the differential abundance analysis (|fold change| ≥ 2 and adjusted *p* < 0.05) at week 4 relative to week 0 in the placebo group (*n* = 19). Species in bold were present in the synbiotic supplement. **Table S4C**. Species of gut bacteria with significant increase or decrease in their abundance (|fold change| ≥ 2 and adjusted *p* < 0.05) at week 6 relative to week 0 in the synbiotic group (*n* = 21). Species in bold were present in the synbiotic supplement.

**Additional file 6: Table S5**. Relative abundances (as % of reads in the synbiotic supplement) for species with significantly increased abundances in the stool at week 4 in the synbiotic group. Only species not added to the supplement are reported and relative abundances were averaged from two samples.

**Additional file 7: Table S6**. Baseline subject characteristics of high- (HR), mid- (MR), and low-responders (LR) in the synbiotics group.

**Additional file 8: Table S7**. Median (IQR) of gut microbial evenness, richness, and α-diversity indices among tertiles of responders (*n* = 23) at sequencing coverage of 380,000 reads.

**Additional file 9: Table S8A**. Bacterial species that were significantly different in the differential abundance analysis (|fold change| ≥ 2 and *p* < 0.05) between high-responders (HR, *n* = 8) and low-responders (LR, *n* = 8) at baseline. Species in bold were present in the synbiotic supplement. **Table S8B**. Bacterial species that were significantly different in the differential abundance analysis (|fold change| ≥ 2 and *p* < 0.05) between high-responders (HR, *n* = 8) and low-responders (LR, *n* = 8) at week 4. Species in bold were present in the synbiotic supplement.

**Additional file 10: Table S9A**. Bacterial species that were significantly different in the differential abundance analysis (|fold change| ≥ 2 and adjusted *p* < 0.05) at week 4 relative to week 0 in high-responders (*n* = 8). Species in bold were present in the synbiotic supplement. **Table S9B**. Bacterial species that were significantly different in the differential abundance analysis (|fold change| ≥ 2 and adjusted *p* < 0.05) at week 4 relative to week 0 in low-responders (*n* = 8).

**Additional file 11: Table S10A**. Gut bacteria from samples collected at baseline that were significantly different in the differential abundance analysis (|fold change| ≥ 2 and *p* < 0.05) between high-responders (HR, *n* = 8) and mid-responders (MR, *n* = 7). Species in red were identified with the same trend when HR was compared with low-responders (LR). **Table S10B**. Gut bacteria from samples collected at baseline that were significantly different in the differential abundance analysis (|fold change| ≥ 2 and *p* < 0.05) between mid-responders (MR, *n* = 7) and low-responders (LR, *n* = 8). Species in red were identified with the same trend when high-responders (HR) was compared with LR.

**Additional file 12: Table S11A**. Gut bacteria from samples collected at week 4 that were significantly different in the differential abundance analysis (|fold change| ≥ 2 and *p* < 0.05) between high-responders (HR, *n* = 8) and mid-responders (MR, *n* = 7). Species in red were identified with the same trend when HR was compared with low-responders (LR). **Table S11B**. Gut bacteria from samples collected at week 4 that were significantly different in the differential abundance analysis (|fold change| ≥ 2 and *p* < 0.05) between mid-responders (MR, *n* = 7) and low-responders (LR, *n* = 8). Species in red were identified with the same trend when high-responders (HR) was compared with LR.

## Data Availability

Survey data included in this published article and its supplementary information files. Fastq files and OTU tables are available from the corresponding author RWH upon request.

## Abbreviations

BCS: Body condition score
FDR: False discovery rate
GI: Gastrointestinal
GM: Gut microbiome
HR: High-responder
KEGG: Kyoto Encyclopedia of Genes and Genomes
KO: KEGG Orthology
LR: Low-responder
MAG: Metagenome-assembled genome
MR: Mid-responder
PCoA: Principal coordinate analysis
PERMANOVA: Permutational multivariate analysis of variance
PL: Placebo
RC: Read count
SD: Standard deviation
SEM: Standard error of the mean
SN: Synbiotic
