# Supplementary figures and images for: The microbiota of healthy dogs demonstrates individualized responses to synbiotic supplementation in a randomized controlled trial
Source: Anim Microbiome. 2021 May 10;3:36. doi: 10.1186/s42523-021-00098-0 (PMC8111948; doi:10.1186/s42523-021-00098-0)

**A**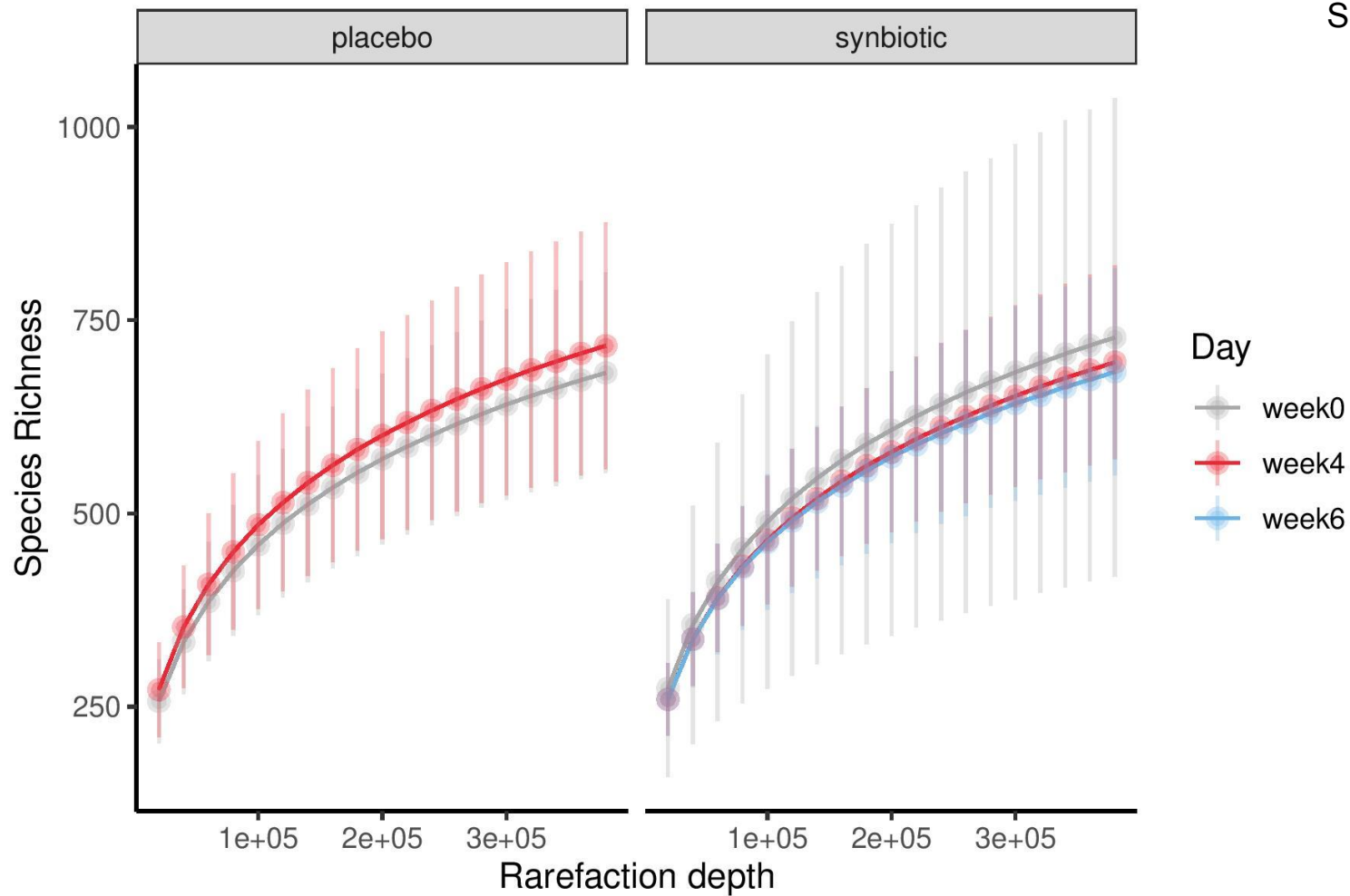

**B**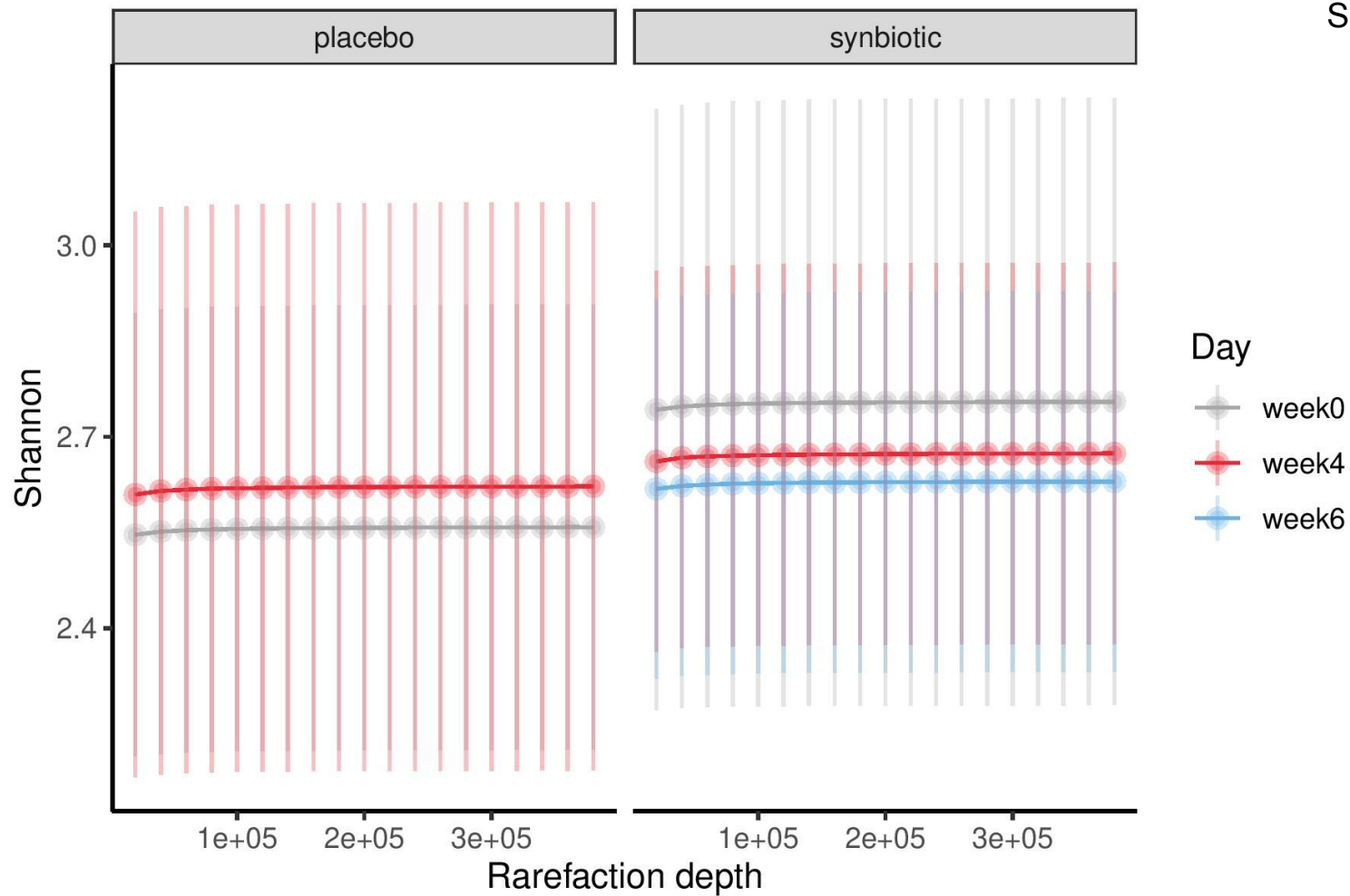

A

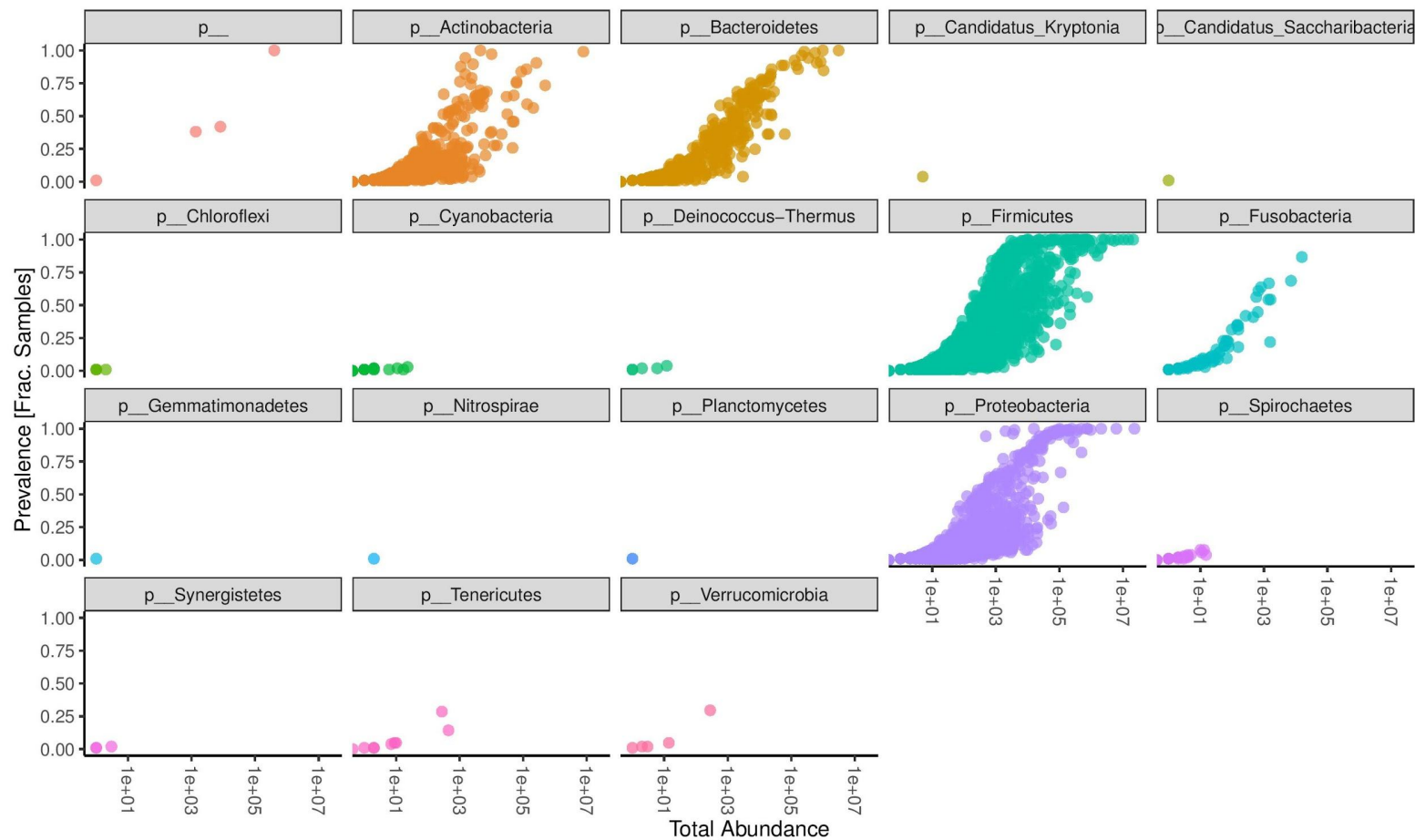

B

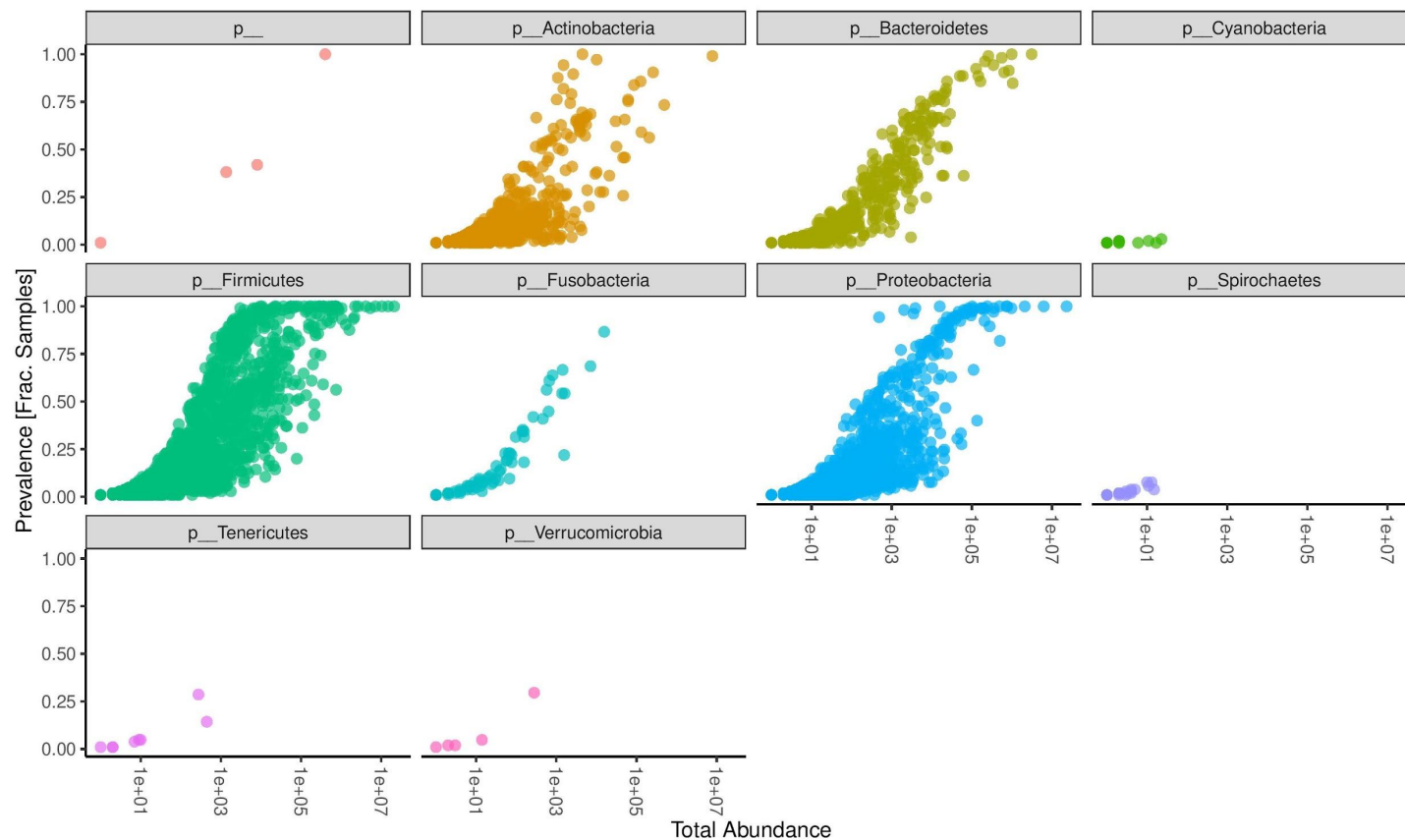

# Bray–Curtis ordination eigenvalues

Sup Fig 3

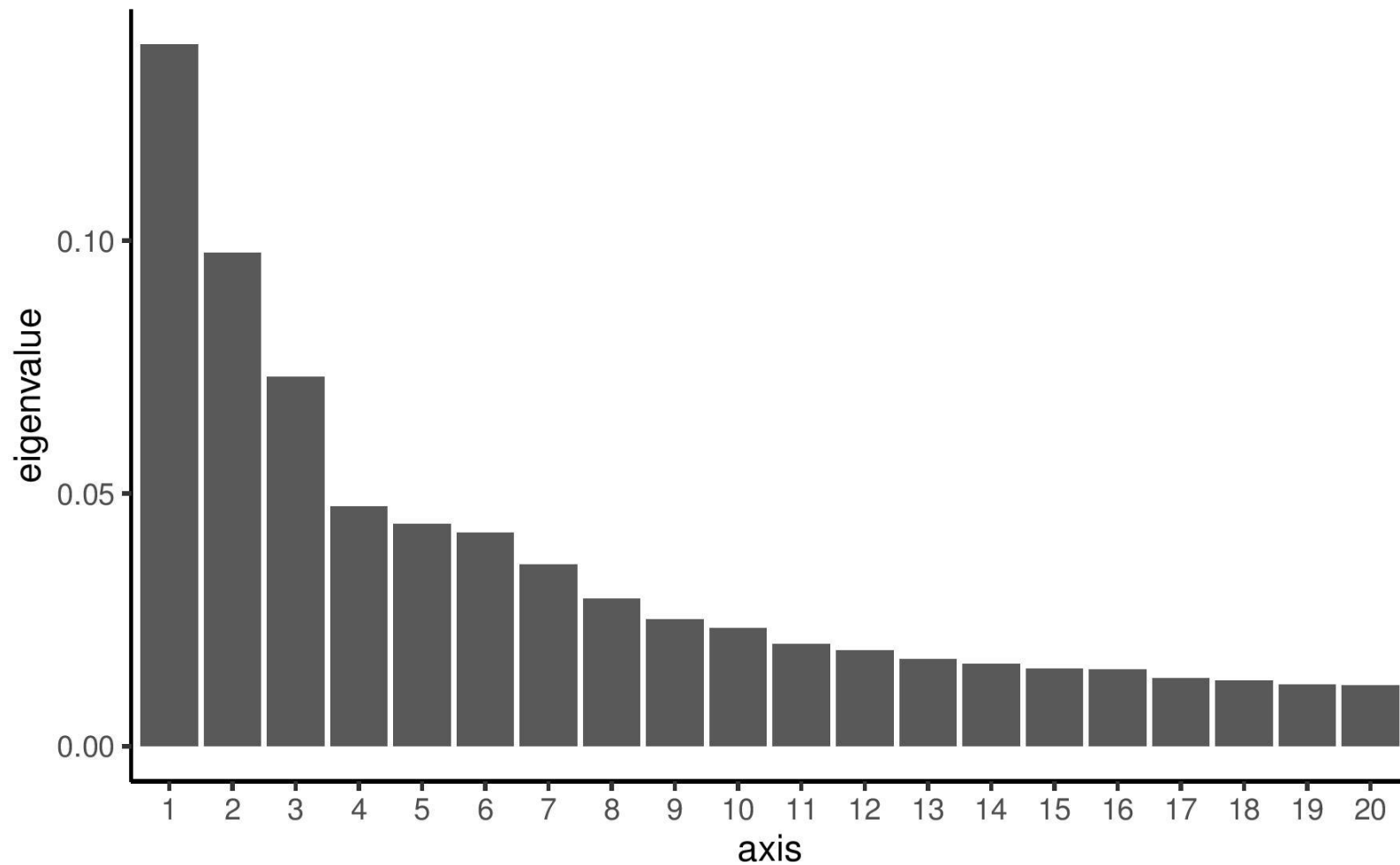

Sup Fig 4

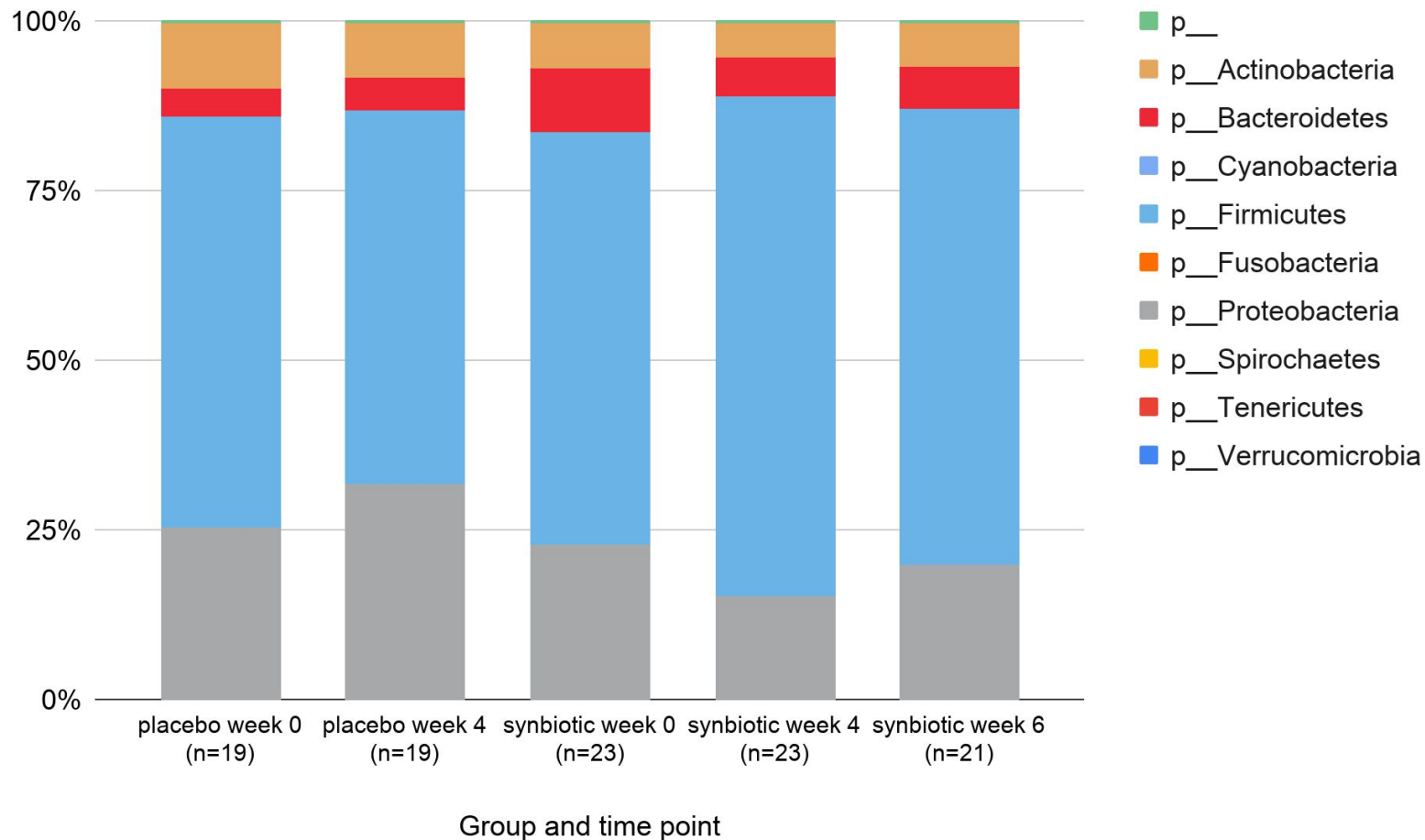

A

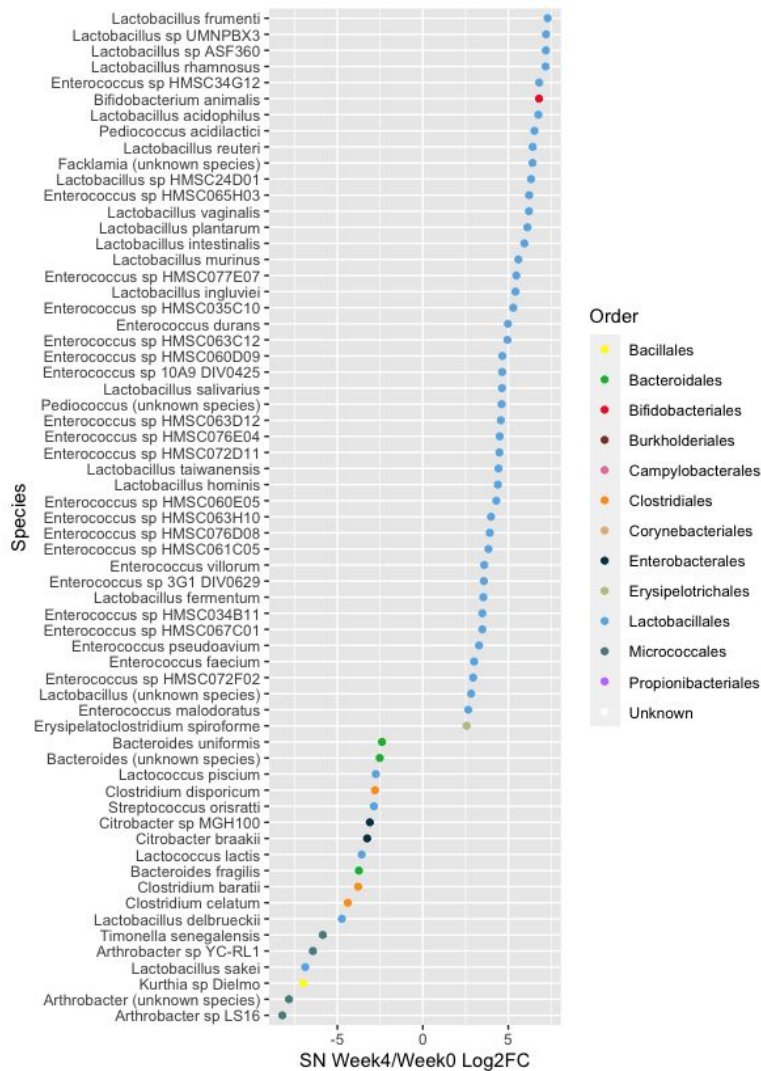

B

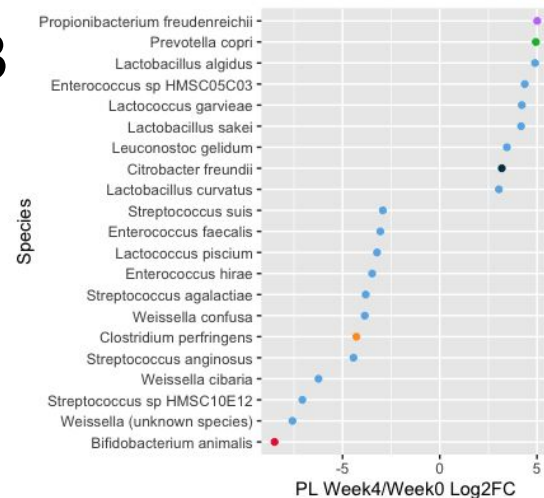

C

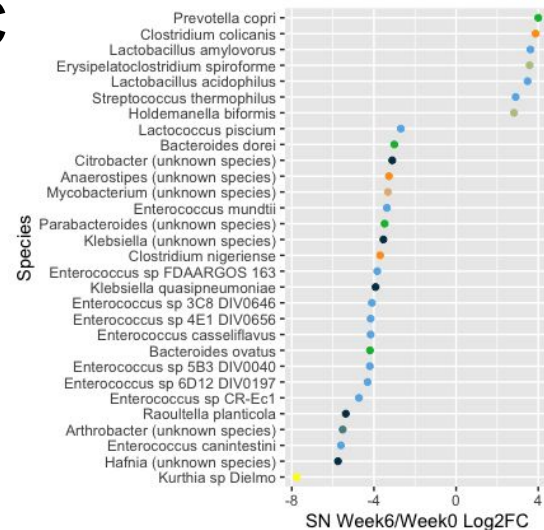

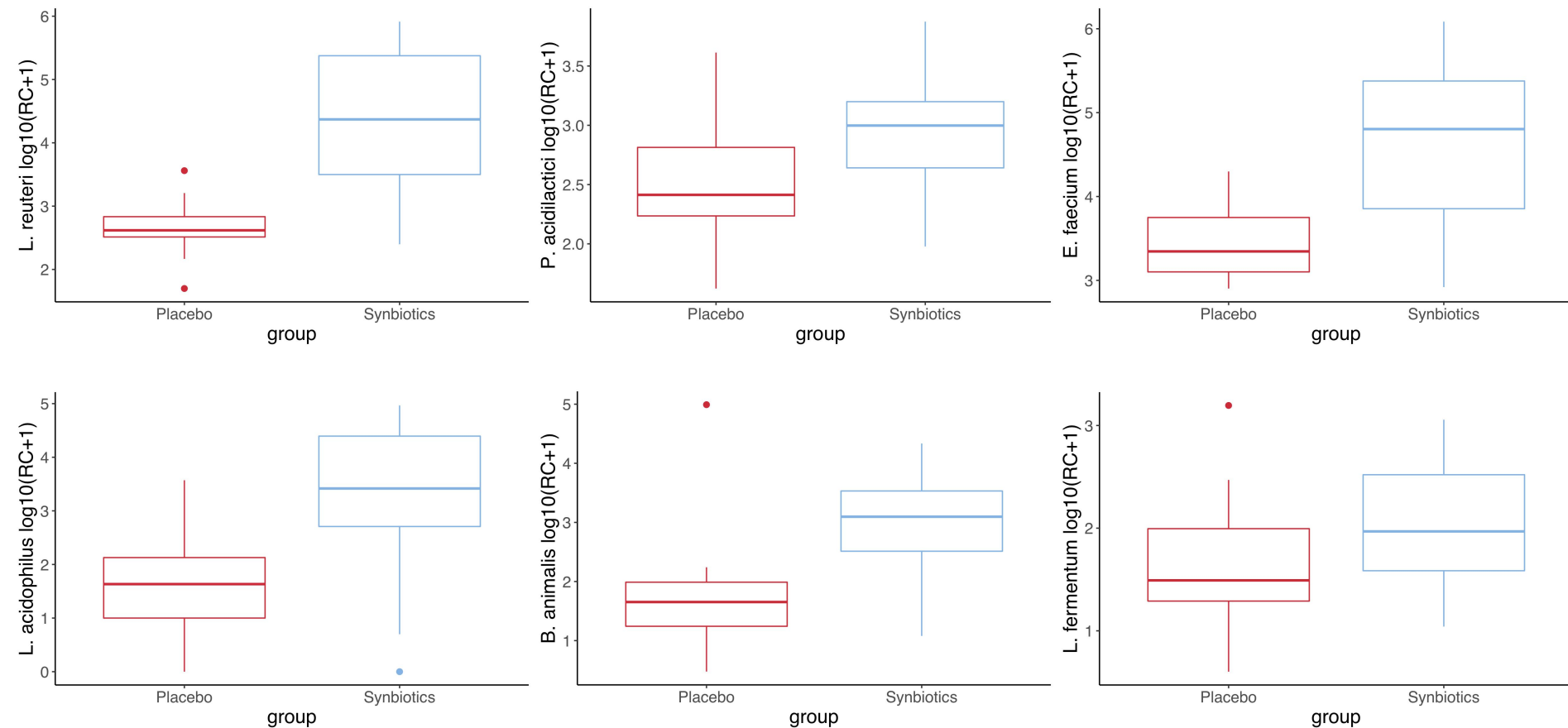

**A**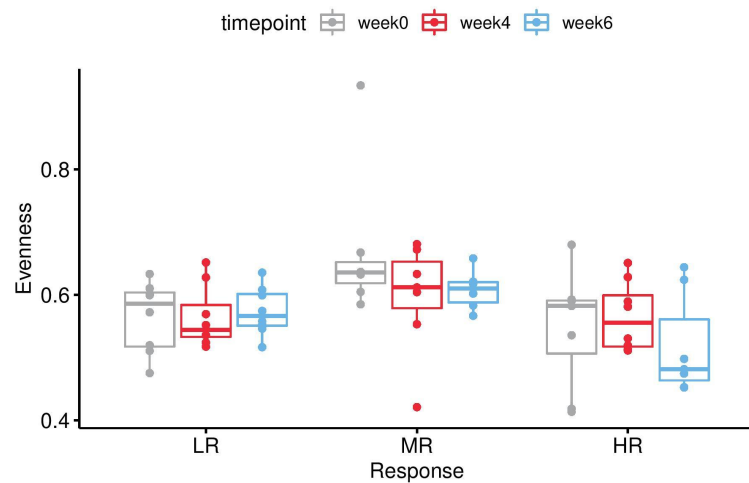**B**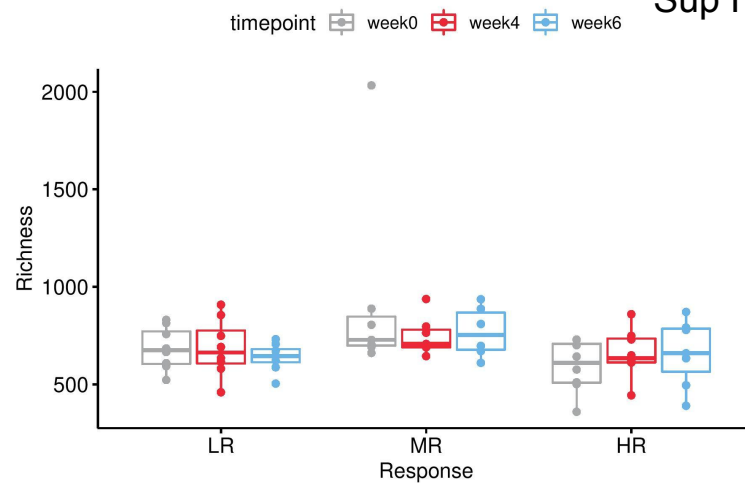**C**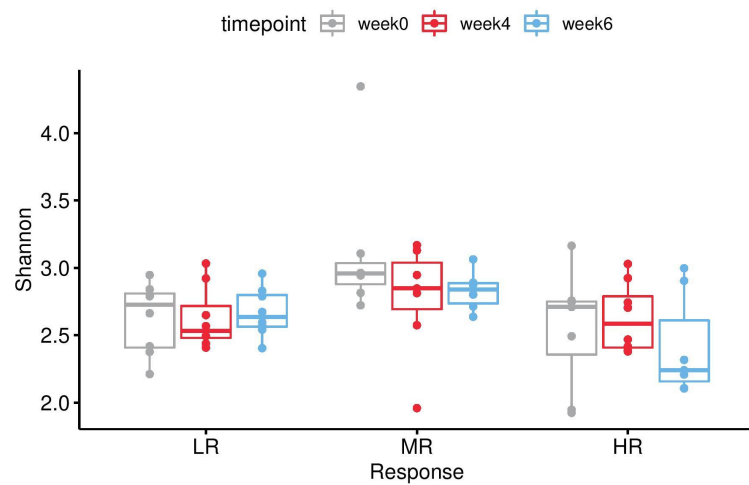**D**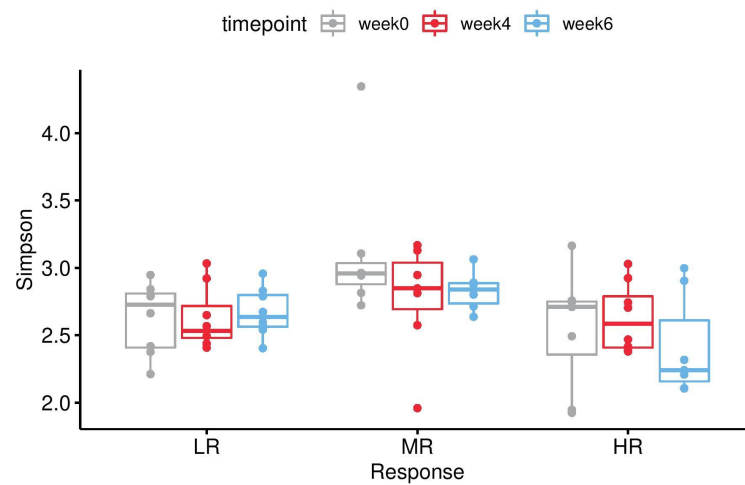

A

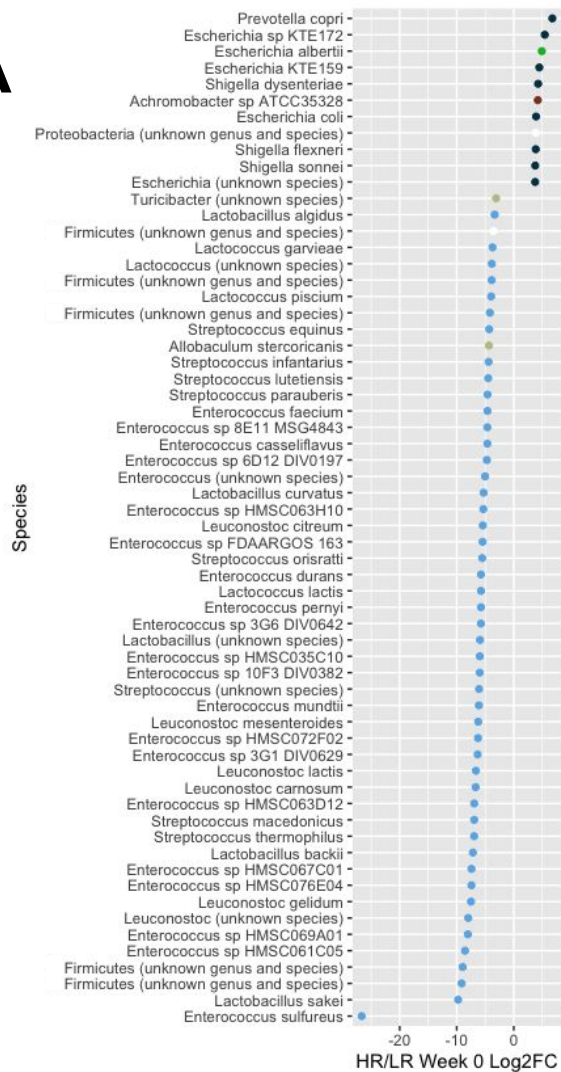

B

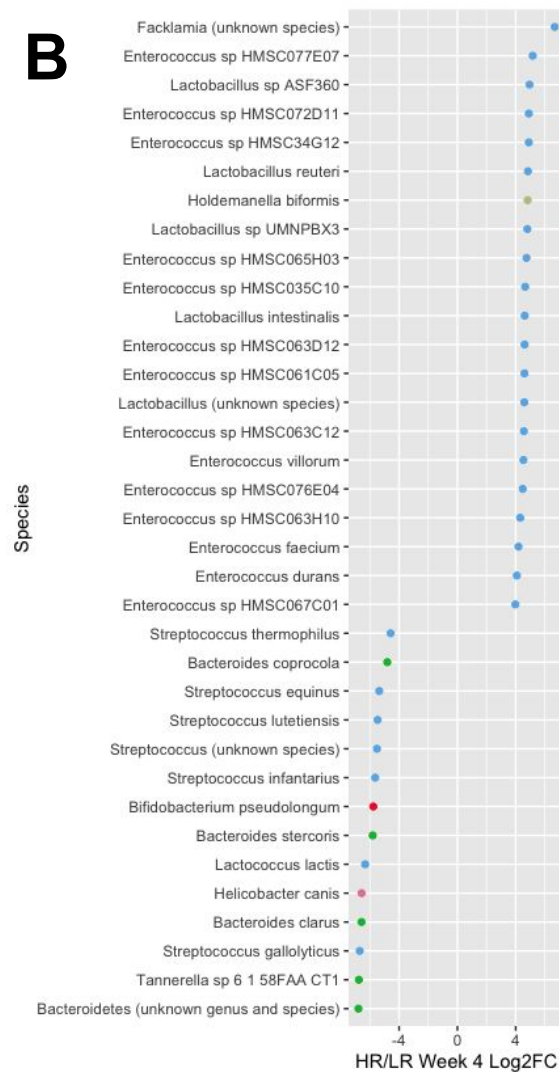

**A**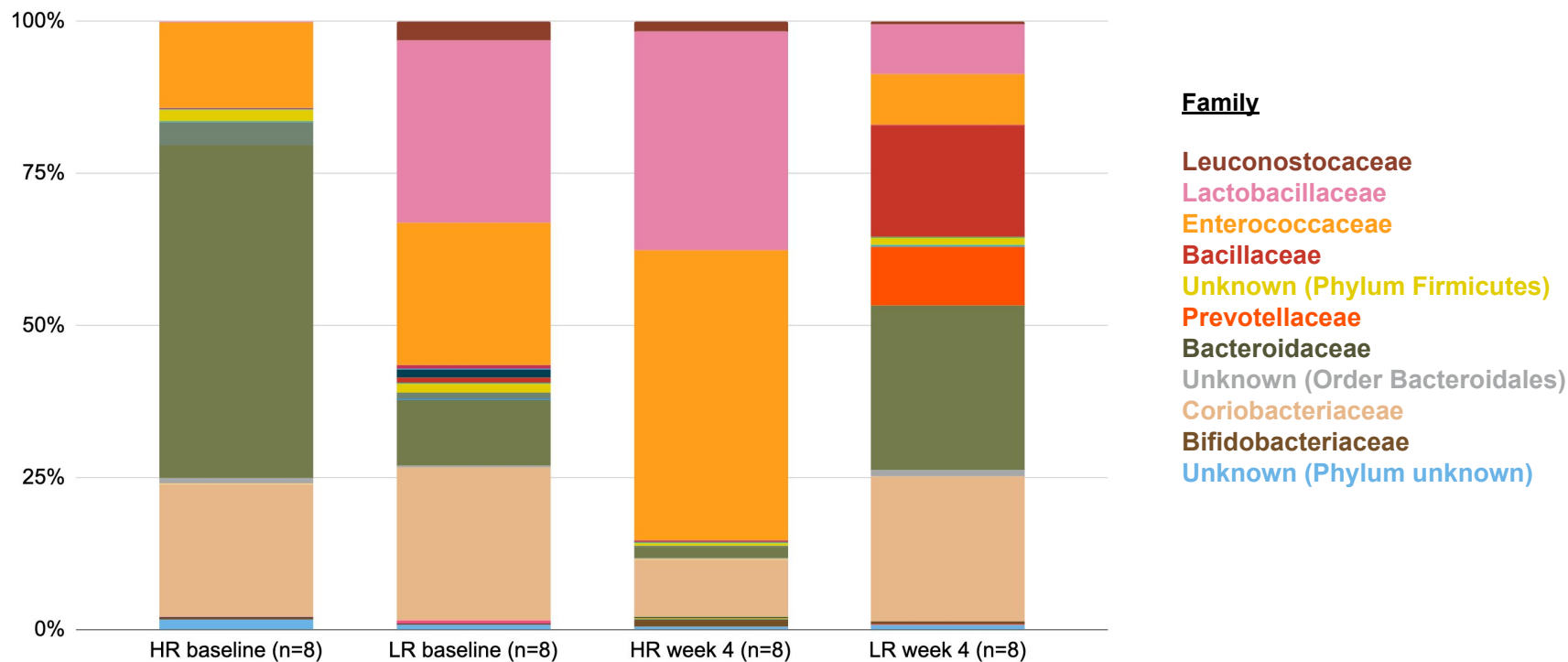

**B**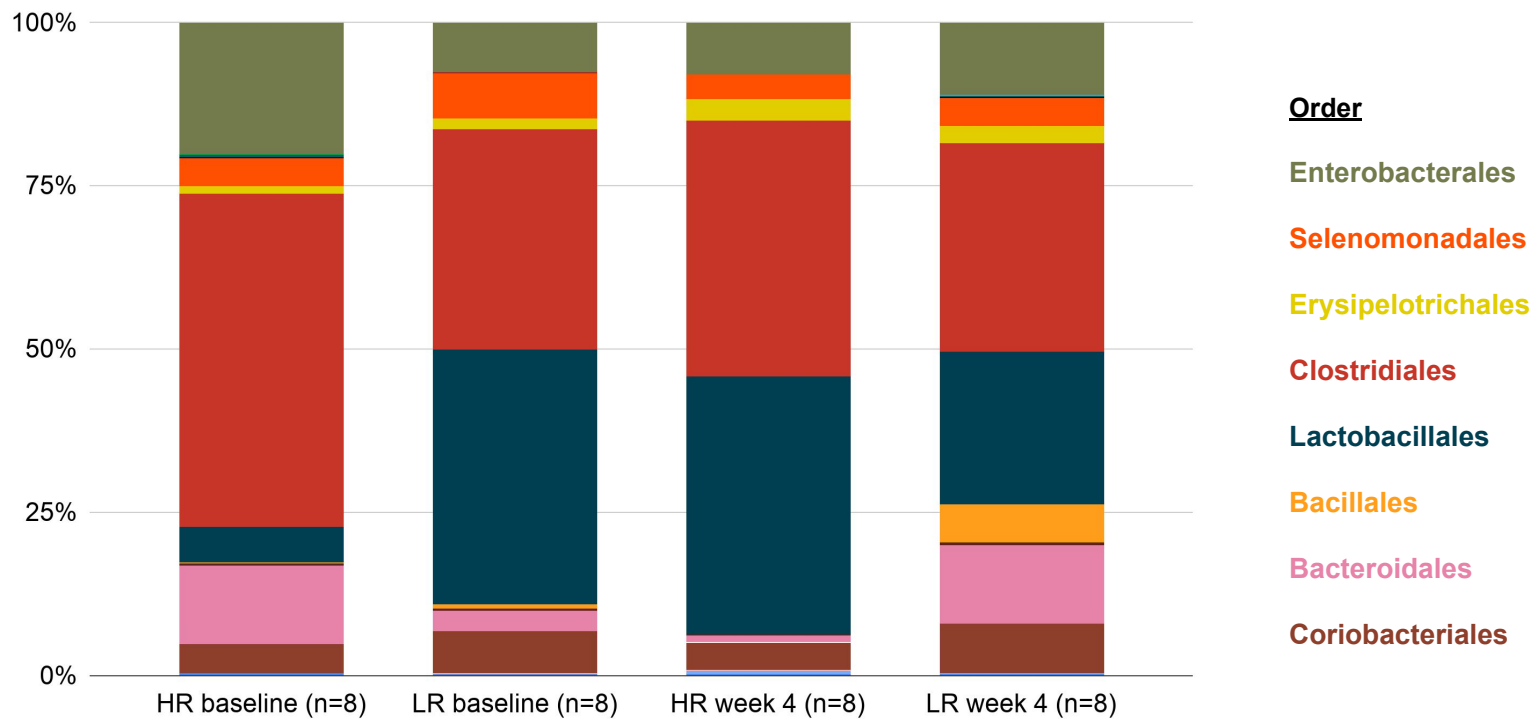

Supplement: Supplementary file 1 — Additional file 1: Figure S1. Rarefaction curves demonstrate sequencing coverage used to calculate species (A) richness and (B) Shannon’s diversity index in subjects receiving synbiotic (n = 23) or placebo (n = 19). Species richness was calculated from 10,000 to 380,000 reads. Each point represents a mean and each error bar represents a standard deviation at each rarefaction depth. Figure S2. Shotgun metagenomic sequencing data quality control. Plots show the abundance (x-axis, as count) and the prevalence (y-axis, as percentage of all samples) of each read for all phyla (A) pre- and (B) post-filtering. Each data point represents a read. As a part of the filtering process, 19 taxa from phyla Candidatus Kryptonia, Candidatus Saccharibacteria, Chloroflexi, Deinococcus-Thermus, Gemmatimonadetes, Nitrospirae, Planctomycetes, Synergistetes were removed because each represented < 5% of samples or belonged to an unknown phylum. Figure S3. Scree plot showing eigenvalues of the first 20 principal coordinate axes. Figure S4. Relative abundance at the phylum level in the samples collected at different time points in dogs receiving placebo or synbiotic. Figure S5. Dot plots demonstrating the fold-change (FC) in the differential abundance analysis of gut bacteria at the species level (A) at week 4 compared to week 0 in the synbiotic group (n = 23); (B) at week 4 compared to week 0 in the placebo group (n = 19); and (C) at week 6 compared to week 0 in the synbiotic group (n = 21). Only species that were significantly different are shown (significance was determined by a negative binomial generalized linear model [GLM] using the differential expression analysis for sequence count data version 2 [DESeq2] package with log2FC at 1 and − 1 [i.e. FC at 2 and − 2] and -log10(adjusted p) = 2 [i.e. adjusted p = 0.01]). Each point represents a different species and points are colored by order. Figure S6. Box plots displaying the number of read counts (RC) in fecal samples collected at week [file 42523_2021_98_MOESM1_ESM.pdf]
