# Supplementary material for: The microbiota of healthy dogs demonstrates individualized responses to synbiotic supplementation in a randomized controlled trial
Source: Anim Microbiome. 2021 May 10;3:36. doi: 10.1186/s42523-021-00098-0 (PMC8111948; doi:10.1186/s42523-021-00098-0)
Supplement: Supplementary file 2 — Additional file 2: Table S1. Guaranteed analysis and ingredients of the 4 cooked diet recipes. [file 42523_2021_98_MOESM2_ESM.docx]

**Supplemental Table 1.** Guaranteed analysis and ingredients of the 4 cooked diet recipes

| **Guaranteed analysis** | **Beef recipe** | **Chicken recipe** | **Pork recipe** | **Turkey recipe** |
| --- | --- | --- | --- | --- |
| Calorie density (kcal/kg)  Crude protein (% as fed)  Crude fat (% as fed)  Crude fiber (% as fed)  Moisture (% as fed) | 1,239  10% min  5% min  1% max  73% max | 1,255  8.5% min  6% min  1% max  77% max | 1,246  8% min  5% min  2% max  75% max | 1,479  11% min  5% min  1% max  70% max |
| Ingredients | Ground beef, russet potatoes, eggs, carrots, peas, dicalcium phosphate, calcium carbonate, salt, fish oil, sunflower oil, vinegar, citric acid, taurine, choline bitartrate, zinc gluconate, ferrous sulfate, vitamin E supplement, copper gluconate, manganese gluconate, thiamine mononitrate, selenium yeast, riboflavin, vitamin B12 supplement, cholecalciferol, potassium iodide | Diced chicken, sweet potatoes, yellow squash, spinach, sunflower oil, canola oil, dicalcium phosphate, calcium carbonate, fish oil, vinegar, citric acid, taurine, choline bitartrate, zinc gluconate, ferrous sulfate, vitamin E supplement, copper gluconate, manganese gluconate, thiamine mononitrate, selenium yeast, riboflavin, vitamin B12 supplement, cholecalciferol, potassium iodide | Ground pork, russet potatoes, green beans, yellow squash, kale, brown mushrooms, dicalcium phosphate, salt, fish oil, vinegar, citric acid, taurine, choline bitartrate, zinc gluconate, ferrous sulfate, vitamin E supplement, copper gluconate, manganese gluconate, thiamine mononitrate, selenium yeast, riboflavin, vitamin B12 supplement, cholecalciferol, potassium iodide | Ground turkey, brown rice, eggs, carrots, spinach, dicalcium phosphate, calcium carbonate, salt, fish oil, vinegar, citric acid, taurine, choline bitartrate, zinc gluconate, ferrous sulfate, vitamin E supplement, copper gluconate, manganese gluconate, thiamine mononitrate, selenium yeast, riboflavin, vitamin B12 supplement, cholecalciferol, potassium iodide |
