# Supplementary material for: The microbiota of healthy dogs demonstrates individualized responses to synbiotic supplementation in a randomized controlled trial
Source: Anim Microbiome. 2021 May 10;3:36. doi: 10.1186/s42523-021-00098-0 (PMC8111948; doi:10.1186/s42523-021-00098-0)
Supplement: Supplementary file 3 — Additional file 3: Table S2. Measures of gut microbial evenness and α-diversity at weeks 0, 4, and 6 at sequencing coverage 380,000 reads. [file 42523_2021_98_MOESM3_ESM.docx]

# **Supplemental Table 2.** Measures of gut microbial evenness and 𝛂-diversity at weeks 0, 4, and 6 at sequencing coverage 380,000 reads

| **Measure** | **Week** | **Synbiotics**  **(SN, n=23)** | | **Placebo**  **(PL, n=19)** | | **P**** | **P***** |
| --- | --- | --- | --- | --- | --- | --- | --- |
|  |  | **Median (IQR)** | **P_vs M0_*** | **Median (IQR)** | **P_vs M0_*** |  |  |
| Evenness  Richness  Shannon’s diversity index  Simpson’s diversity index | 0  4  6  0  4  6  0  4  6  0  4  6 | 0.59 (0.55-0.63)  0.60 (0.53-0.63)  0.57 (0.53-0.63)  692 (601-744)  690 (618-757)  670 (622-779)  2.76 (2.58-2.95)  2.65 (2.45-2.92)  2.67 (2.40-2.88)  2.76 (2.58-2.95)  2.65 (2.45-2.92)  2.67 (2.40-2.88) | NA  0.870  0.393  NA  0.823  0.633  NA  0.870  0.393  NA  0.870  0.393 | 0.57 (0.50-0.60)  0.55 (0.51-0.60)  NA  677 (592-757)  694 (622-763)  NA  2.63 (2.31-2.80)  2.58 (2.38-2.81)  NA  2.63 (2.31-2.80)  2.58 (2.38-2.81)  NA | NA  0.568  NA  NA  0.294  NA  NA  0.568  NA  NA  0.568  NA | 0.137  0.548  NA  0.960  0.920  NA  0.137  0.548  NA  0.137  0.548  NA | NA  0.653  NA  NA  0.617  NA  NA  0.653  NA  NA  0.653  NA |

* Wilcoxon signed-rank test comparing week 4 or 6 and week 0 within each group.

** Wilcoxon rank sum test comparing each time point between SN and PL.

*** Wilcoxon rank sum test comparing changes from week 0 to week 4 between SN and PL.

NA: not applicable.
