# Supplementary material for: The microbiota of healthy dogs demonstrates individualized responses to synbiotic supplementation in a randomized controlled trial
Source: Anim Microbiome. 2021 May 10;3:36. doi: 10.1186/s42523-021-00098-0 (PMC8111948; doi:10.1186/s42523-021-00098-0)
Supplement: Supplementary file 4 — Additional file 4: Table S3. Breeds and diet of study participants (placebo n = 19, synbiotic n = 24). [file 42523_2021_98_MOESM4_ESM.docx]

**Supplemental Table 3.** Breeds and diet of study participants (placebo n=19, synbiotic n=24)

| **Subject** | **Group** | **Age, in years** | **Sex** | **Breed** | **Body Condition Score*** | **Recipe**** |
| --- | --- | --- | --- | --- | --- | --- |
| S01 | PL | 6 | M | Zuchon (Bichon Frise/Shih Tzu mix) | 6 | B,T |
| S03 | PL | 8 | F | Yorkshire Terrier | 4-5 | B |
| S05 | PL | 2 | M | Brussels Griffon | 4-5 | P |
| S06 | PL | 7 | M | Havanese | 4-5 | B,P |
| S12 | PL | 5 | M | Dachshund (miniature) | 4-5 | T |
| S16 | PL | 10 | F | Dachshund (miniature) | 4-5 | B,T |
| S17 | PL | 8 | M | Japanese Chin | 4-5 | T |
| S19 | PL | 4 | M | French Bulldog | 4-5 | B,C,P,T |
| S22 | PL | 7 | M | Miniature Pinscher | 6 | B |
| S25 | PL | 4 | M | Boston Terrier | 4-5 | B,P |
| S27 | PL | 9 | F | Mixed | 4-5 | P |
| S29 | PL | 6 | M | No data | 7 | P |
| S33 | PL | 11 | M | Bichon Frise | 4-5 | B,C,P,T |
| S36 | PL | 3 | F | Yorkshire Terrier | 4-5 | B,C,P,T |
| S37 | PL | 3 | M | Great Pyrenees/German Shepherd mix | 4-5 | P |
| S46 | PL | 11 | F | Chihuahua | 6 | B,C,P,T |
| S47 | PL | 9 | M | Weimaraner | 6 | P |
| S50 | PL | 7 | M | Boxer | 4-5 | C,T |
| S51 | PL | 1 | M | Maltese | 6 | C,T |
| S02 | SN, MR | 9 | F | No data | 4-5 | C |
| S04 | SN, HR | 3 | F | Pit Bull Terrier | 3 | P |
| S09 | SN, LR | 9 | F | Australian Cattle Dog | 6 | B,T |
| S10 | SN, HR | 5 | M | American Cocker Spaniel | 4-5 | B |
| S11 | SN, LR | 1 | M | Maltese | 4-5 | B |
| S13 | SN, HR | 3 | M | Biewer Yorkie | 4-5 | B |
| S14 | SN, HR | 2 | M | Goldendoodle | 4-5 | B,T |
| S15 | SN, LR | 11 | M | Chiweenie | 4-5 | T |
| S20 | SN, LR | 9 | M | Poodle Mix | 4-5 | C,T |
| S21 | SN, MR | 6 | M | Pointer/Chihuahua Mix | 4-5 | T |
| S23 | SN, LR | 3 | F | Schnauzer (miniature) | 4-5 | B,C |
| S24 | SN, LR | 7 | M | Shetland Sheepdog (sheltie) | 4-5 | T |
| S28 | SN, LR | 3 | M | Dachshund (miniature) | 4-5 | C |
| S30 | SN, MR | 3 | M | Chihuahua | 4-5 | T |
| S32*** | SN | 5 | M | Chihuahua | 4-5 | P |
| S35 | SN, HR | 8 | M | Daschund (miniature) | 6 | B |
| S38 | SN, HR | 4 | F | Cavalier King Charles Spaniel | 4-5 | B,P |
| S39 | SN, MR | 3 | M | American Staffordshire Terrier | 4-5 | T |
| S40 | SN, HR | 1 | F | Schnoodle | 4-5 | B,C |
| S41 | SN, MR | 5 | M | Jack Russell Terrier | 4-5 | B,C |
| S42 | SN, MR | 1 | F | American Staffordshire Terrier | 4-5 | T |
| S43 | SN, LR | 3 | F | Boston Terrier | 4-5 | P |
| S45 | SN, HR | 5 | M | Yorkshire Terrier | 4-5 | T |
| S49 | SN, MR | 10 | F | Cavalier King Charles Spaniel/Bichon Frise mix | 3 | C,T |

PL: placebo, SN: synbiotics, LR: low-responder, MR: mid-responder, HR: high-responder (see section 3.3.1 for the definition of LR, MR, HR)

*9-point body condition score: 3 (mildly underweight), 4-5 (normal weight), 6 (mildly overweight), 7 (overweight)

**Each participant was on one, two, or four of the four available recipes which were B (beef), C (chicken), P (pork), and T (turkey). Ingredients and nutritional profiles of each recipe are available in Supplemental Table 1 and [www.nomnomnow.com](http://www.nomnomnow.com)

*** S32 not included in the gut microbiome analysis because fecal sample was not processed on the same batch as other samples
