# Supplementary material for: The microbiota of healthy dogs demonstrates individualized responses to synbiotic supplementation in a randomized controlled trial
Source: Anim Microbiome. 2021 May 10;3:36. doi: 10.1186/s42523-021-00098-0 (PMC8111948; doi:10.1186/s42523-021-00098-0)
Supplement: Supplementary file 5 — Additional file 5: Table S4A. Species of gut bacteria with significant increase or decrease in the differential abundance analysis (|fold change| ≥ 2 and adjusted p < 0.05) at week 4 relative to week 0 in the synbiotic group (n = 23). Species in bold were present in the synbiotic supplement. Table S4B. Species of gut bacteria with significant increase or decrease in the differential abundance analysis (|fold change| ≥ 2 and adjusted p < 0.05) at week 4 relative to week 0 in the placebo group (n = 19). Species in bold were present in the synbiotic supplement. Table S4C. Species of gut bacteria with significant increase or decrease in their abundance (|fold change| ≥ 2 and adjusted p < 0.05) at week 6 relative to week 0 in the synbiotic group (n = 21). Species in bold were present in the synbiotic supplement. [file 42523_2021_98_MOESM5_ESM.docx]

**Supplemental Table 4A.** Species of gut bacteria with significant increase or decrease in the differential abundance analysis (|fold change| ≥ 2 and adjusted p < 0.05) at week 4 relative to week 0 in the synbiotic group (n=23). Species in bold were present in the synbiotic supplement.

| **Phylum** | **Class** | **Order** | **Family** | **Genus** | **Species** | **DESeq2 results**  **week 4/week 0** | | **Relative abundance, in %** | | | |
| --- | --- | --- | --- | --- | --- | --- | --- | --- | --- | --- | --- |
|  |  |  |  |  |  | **Log 2 FC***  **Mean ± SE** | **Adjusted p**** | **Week 0**  **Median (IQR)** | | **Week 4**  **Median (IQR)** | |
| **Increased at week 4** | | | | | | | | | | | |
| Firmicutes | Bacilli | Lactobacillales | Lactobacillaceae | Lactobacillus | frumenti | 7.30 ± 1.21 | < 0.001 | 0.00E+0 (0.00E+0 - 0.00E+0) | | 2.23E-4 (0.00E+0 - 3.23E-3) | |
| Firmicutes | Bacilli | Lactobacillales | Lactobacillaceae | Lactobacillus | sp UMNPBX3 | 7.21 ± 1.11 | < 0.001 | 0.00E+0 (0.00E+0 - 9.77E-5) | | 5.25E-3 (6.56E-4 - 3.98E-2) | |
| Firmicutes | Bacilli | Lactobacillales | Lactobacillaceae | Lactobacillus | sp ASF360 | 7.20 ± 1.16 | < 0.001 | 0.00E+0 (0.00E+0 - 0.00E+0) | | 2.00E-4 (3.39E-5 - 2.72E-5) | |
| **Firmicutes** | **Bacilli** | **Lactobacillales** | **Lactobacillaceae** | **Lactobacillus** | **rhamnosus** | 7.18 ± 1.35 | < 0.001 | 0.00E+0 (0.00E+0 - 0.00E+0) | | 8.32E-5 (0.00E+0 - 1.76E-3) | |
| Firmicutes | Bacilli | Lactobacillales | Enterococcaceae | Enterococcus | sp HMSC34G12 | 6.81 ± 1.07 | < 0.001 | 0.00E+0 (0.00E+0 - 0.00E+0) | | 1.67E-3 (1.46E-4 - 6.35E-3) | |
| **Firmicutes** | **Actinobacteria** | **Bifidobacteriales** | **Bifidobacteriaceae** | **Bifidobacterium** | **animalis** | 6.80 ± 0.93 | < 0.001 | 1.53E-4 (0.00E+0 - 5.96E-4) | | 7.40E-2 (1.01E-2 - 1.51E-1) | |
| **Firmicutes** | **Bacilli** | **Lactobacillales** | **Lactobacillaceae** | **Lactobacillus** | **acidophilus** | 6.76 ± 0.88 | < 0.001 | 2.96E-3 (9.22E-4 - 2.49E-2) | | 1.70E-1 (4.14E-2 - 2.72E+0) | |
| **Firmicutes** | **Bacilli** | **Lactobacillales** | **Lactobacillaceae** | **Pediococcus** | **acidilactici** | 6.53 ± 1.10 | < 0.001 | 0.00E+0 (0.00E+0 - 7.01E-5) | | 7.85E-3 (6.98E-4 - 3.94E-2) | |
| **Firmicutes** | **Bacilli** | **Lactobacillales** | **Lactobacillaceae** | **Lactobacillus** | **reuteri** | 6.43 ± 1.00 | < 0.001 | 1.46E-3 (4.80E-4 - 1.84E-2) | | 1.09E+0 (1.43E-1 - 8.96E+0) | |
| Firmicutes | Bacilli | Lactobacillales | Aerococcaceae | Facklamia | ND | 6.42 ± 1.23 | < 0.001 | 0.00E+0 (0.00E+0 - 0.00E+0) | | 3.35E-4 (0.00E+0 - 1.04E-3) | |
| Firmicutes | Bacilli | Lactobacillales | Lactobacillaceae | Lactobacillus | sp HMSC24D01 | 6.33 ± 1.15 | < 0.001 | 0.00E+0 (0.00E+0 - 0.00E+0) | | 1.33E-4 (3.33E-05 - 8.80E-4) | |
| Firmicutes | Bacilli | Lactobacillales | Enterococcaceae | Enterococcus | sp HMSC065H03 | 6.22 ± 1.09 | < 0.001 | 0.00E+0 (0.00E+0 - 0.00E+0) | | 2.71E-4 (0.00E+0 - 8.51E-4) | |
| Firmicutes | Bacilli | Lactobacillales | Lactobacillaceae | Lactobacillus | vaginalis | 6.21 ± 1.16 | < 0.001 | 0.00E+0 (0.00E+0 - 0.00E+0) | | 4.66E-4 (3.07E-5 - 1.53E-3) | |
| Firmicutes | Bacilli | Lactobacillales | Lactobacillaceae | Lactobacillus | plantarum | 6.12 ± 0.97 | < 0.001 | 2.44E-4 (5.88E-5 - 5.32E-4) | | 3.55E-3 (4.85E-4 - 9.96E-2) | |
| Firmicutes | Bacilli | Lactobacillales | Lactobacillaceae | Lactobacillus | intestinalis | 5.94 ± 1.27 | < 0.001 | 0.00E+0 (0.00E+0 - 0.00E+0) | | 9.99E-4 (3.39E-5 - 5.95E-3) | |
| Firmicutes | Bacilli | Lactobacillales | Lactobacillaceae | Lactobacillus | murinus | 5.59 ± 1.22 | < 0.001 | 0.00E+0 (0.00E+0 - 0.00E+0) | | 8.32E-5 (0.00E+0 - 6.29E-4) | |
| Firmicutes | Bacilli | Lactobacillales | Enterococcaceae | Enterococcus | sp HMSC077E07 | 5.47 ± 1.26 | < 0.001 | 0.00E+0 (0.00E+0 - 0.00E+0) | | 1.10E-4 (0.00E+0 - 4.38E-4) | |
| Firmicutes | Bacilli | Lactobacillales | Lactobacillaceae | Lactobacillus | ingluviei | 5.42 ± 1.23 | < 0.001 | 0.00E+0 (0.00E+0 - 0.00E+0) | | 1.12E-4 (0.00E+0 - 7.35E-4) | |
| Firmicutes | Bacilli | Lactobacillales | Enterococcaceae | Enterococcus | sp HMSC035C10 | 5.29 ± 0.91 | < 0.001 | 1.04E-4 (0.00E+0 - 4.66E-4) | | 5.75E-3 (8.12E-4 - 1.75E-2) | |
| Firmicutes | Bacilli | Lactobacillales | Enterococcaceae | Enterococcus | durans | 4.98 ± 0.87 | < 0.001 | 3.67E-4 (7.08E-5 - 2.84E-3) | | 8.93E-3 (1.91E-3 - 2.72E-2) | |
| Firmicutes | Bacilli | Lactobacillales | Enterococcaceae | Enterococcus | sp HMSC063C12 | 4.95 ± 1.07 | < 0.001 | 0.00E+0 (0.00E+0 - 0.00E+0) | | 1.10E-4 (0.00E+0 - 3.48E-4) | |
| Firmicutes | Bacilli | Lactobacillales | Enterococcaceae | Enterococcus | sp HMSC060D09 | 4.65 ± 1.25 | 0.005 | 0.00E+0 (0.00E+0 - 0.00E+0) | | 6.13E-5 (0.00E+0 - 2.48E-4) | |
| Firmicutes | Bacilli | Lactobacillales | Enterococcaceae | Enterococcus | sp 10A9 DIV0425 | 4.64 ± 0.96 | < 0.001 | 0.00E+0 (0.00E+0 - 1.11E-4) | | 7.79E-4 (2.22E-4 - 2.78E-3) | |
| Firmicutes | Bacilli | Lactobacillales | Lactobacillaceae | Lactobacillus | salivarius | 4.63 ± 0.84 | < 0.001 | 4.47E-5 (0.00E+0 - 6.81E-5) | | 5.58E-4 (7.86E-5 - 2.13E-3) | |
| Firmicutes | Bacilli | Lactobacillales | Lactobacillaceae | Pediococcus | ND | 4.61 ± 1.34 | 0.012 | 0.00E+0 (0.00E+0 - 0.00E+0) | | 0.00E+0 (0.00E+0 - 3.05E-4) | |
| Firmicutes | Bacilli | Lactobacillales | Enterococcaceae | Enterococcus | sp HMSC063D12 | 4.56 ± 0.98 | < 0.001 | 3.05E-4 ( 0.00E+0 - 6.21E-4) | | 8.54E-2 (7.52E-3 - 2.78E-1) | |
| Firmicutes | Bacilli | Lactobacillales | Enterococcaceae | Enterococcus | sp HMSC076E04 | 4.50 ± 1.03 | < 0.001 | 6.26E-5 (0.00E+0 - 3.43E-4) | | 4.64E-2 (4.94E-3 - 1.49E-1) | |
| Firmicutes | Bacilli | Lactobacillales | Enterococcaceae | Enterococcus | sp HMSC072D11 | 4.48 ± 1.09 | 0.001 | 0.00E+0 (0.00E+0 - 0.00E+0) | | 6.74E-4 (2.79E-5 - 2.33E-3) | |
| Firmicutes | Bacilli | Lactobacillales | Lactobacillaceae | Lactobacillus | taiwanensis | 4.43 ± 0.95 | < 0.001 | 0.00E+0 (0.00E+0 - 5.28E-5) | | 2.01E-4 (2.79E-5 - 6.40E-4) | |
| Firmicutes | Bacilli | Lactobacillales | Lactobacillaceae | Lactobacillus | hominis | 4.39 ± 1.38 | 0.026 | 0.00E+0 (0.00E+0 - 0.00E+0) | | 1.33E-4 (0.00E+0 - 2.97E-3) | |
| Firmicutes | Bacilli | Lactobacillales | Enterococcaceae | Enterococcus | sp HMSC060E05 | 4.30 ± 1.13 | 0.003 | 0.00E+0 (0.00E+0 - 0.00E+0) | | 1.23E-4 (0.00E+0 - 2.98E-4) | |
| Firmicutes | Bacilli | Lactobacillales | Enterococcaceae | Enterococcus | sp HMSC063H10 | 3.99 ± 0.85 | < 0.001 | 1.10E-4 (5.03E-5 - 7.89E-4) | | 2.93E-3 (5.83E-4 - 1.17E-2) | |
| Firmicutes | Bacilli | Lactobacillales | Enterococcaceae | Enterococcus | sp HMSC076D08 | 3.92 ± 1.31 | 0.041 | 0.00E+0 (0.00E+0 - 0.00E+0) | | 5.37e-5 (0.00E+0 - 1.54E-4) | |
| Firmicutes | Bacilli | Lactobacillales | Enterococcaceae | Enterococcus | sp HMSC061C05 | 3.84 ± 1.07 | 0.008 | 4.47E-5 (0.00E+0 - 2.60E-4) | | 3.16E-2 (2.93E-3 - 9.71E-2) | |
| Firmicutes | Bacilli | Lactobacillales | Enterococcaceae | Enterococcus | villorum | 3.59 ± 0.88 | 0.001 | 0.00E+0 (0.00E+0 - 7.63E-5) | | 4.19E-4 (8.32E-5 - 1.84E-3) | |
| Firmicutes | Bacilli | Lactobacillales | Enterococcaceae | Enterococcus | sp 3G1 DIV0629 | 3.57 ± 1.08 | 0.018 | 0.00E+0 (0.00E+0 - 3.27E-4) | | 3.94E-3 (7.11E-4 - 1.40E-2) | |
| **Firmicutes** | **Bacilli** | **Lactobacillales** | **Lactobacillaceae** | **Lactobacillus** | **fermentum** | 3.54 ± 0.88 | 0.002 | 1.26E-4 (6.79E-5 - 1.02E-3) | | 7.08E-3 (3.70E-4 - 2.81E-2) | |
| Firmicutes | Bacilli | Lactobacillales | Enterococcaceae | Enterococcus | sp HMSC067C01 | 3.48 ± 1.01 | 0.012 | 7.63E-5 (0.00E+0 - 6.23E-4) | | 3.39E-2 (5.02E-3 - 1.10E-1) | |
| Firmicutes | Bacilli | Lactobacillales | Enterococcaceae | Enterococcus | sp HMSC034B11 | 3.48 ± 1.07 | 0.021 | 0.00E+0 (0.00E+0 - 5.19E-5) | | 1.36E-4 (0.00E+0 - 3.48E-4) | |
| Firmicutes | Bacilli | Lactobacillales | Enterococcaceae | Enterococcus | pseudoavium | 3.29 ± 1.04 | 0.027 | 0.00E+0 (0.00E+0 - 2.48E-5) | | 2.68E-4 (0.00E+0 - 6.06E-4) | |
| **Firmicutes** | **Bacilli** | **Lactobacillales** | **Enterococcaceae** | **Enterococcus** | **faecium** | 3.00 ± 0.82 | 0.006 | 1.04E-3 (1.30E-4 - 6.30E-3) | | 3.99E-2 (6.22E-3 - 1.19E-1) | |
| Firmicutes | Bacilli | Lactobacillales | Enterococcaceae | Enterococcus | sp HMSC072F02 | 2.94 ± 0.99 | 0.045 | 1.83E-4 (0.00E+0 - 1.07E-3) | | 1.49E-2 (2.25E-3 - 5.02E-2) | |
| Firmicutes | Bacilli | Lactobacillales | Lactobacillaceae | Lactobacillus | ND | 2.83 ± 0.96 | 0.047 | 3.72E-3 (9.24E-4 - 1.10E-2) | | 2.51E-1 (2.07E-2 - 1.33E+0) | |
| Firmicutes | Bacilli | Lactobacillales | Enterococcaceae | Enterococcus | malodoratus | 2.66 ± 0.80 | 0.019 | 0.00E+0 (0.00E+0 - 1.92E-4) | | 3.77E-4 (6.46E-5 - 1.15E-3) | |
| Firmicutes | Erysipelotrichia | Erysipelotrichales | Erysipelotrichaceae | Erysipelatoclostridium | spiroforme | 2.56 ± 0.80 | 0.026 | 1.09E-4 (0.00E+0 - 4.04E-4) | | 2.25E-4 (2.69E-5 - 6.08E-4) | |
| **Decreased at week 4** | | | | | | | | | | | |
| Actinobacteria | Actinobacteria | Micrococcales | Micrococcaceae | Arthrobacter | sp LS16 | -8.22 ± 2.36 | 0.011 | 0.00E+0 (0.00E+0 - 2.75E-5) | | 0.00E+0 (0.00E+0 - 0.00E+0) | |
| Actinobacteria | Actinobacteria | Micrococcales | Micrococcaceae | Arthrobacter | ND | -7.83 ± 1.45 | < 0.001 | 5.80E-5 (0.00E+0 - 2.64E-4) | | 0.00E+0 (0.00E+0 - 7.17E-5) | |
| Firmicutes | Bacilli | Bacillales | Planococcaceae | Kurthia | sp Dielmo | -7.00 ± 2.11 | 0.018 | 0.00E+0 (0.00E+0 - 6.79E-5) | | 0.00E+0 (0.00E+0 - 0.00E+0) | |
| Firmicutes | Bacilli | Lactobacillales | Lactobacillaceae | Lactobacillus | sakei | -6.88 ± 1.04 | < 0.001 | 2.86E-3 (4.95E-4 - 2.65E-2) | | 6.53E-4 (2.91E-4 - 4.96E-3) | |
| Actinobacteria | Actinobacteria | Micrococcales | Micrococcaceae | Arthrobacter | sp YC-RL1 | -6.43 ± 1.95 | 0.019 | 0.00E+0 (0.00E+0 - 1.39E-4) | | 0.00E+0 (0.00E+0 - 0.00E+0) | |
| Actinobacteria | Actinobacteria | Micrococcales | ND | Timonella | senegalensis | -5.85 ± 1.96 | 0.044 | 0.00E+0 (0.00E+0 - 2.07E-4) | | 0.00E+0 (0.00E+0 - 0.00E+0) | |
| Firmicutes | Bacilli | Lactobacillales | Lactobacillaceae | Lactobacillus | delbrueckii | -4.74 ± 1.55 | 0.035 | 0.00E+0 (0.00E+0 - 2.17E-4) | | 0.00E+0 (0.00E+0 - 0.00E+0) | |
| Firmicutes | Clostridia | Clostridiales | Clostridiaceae | Clostridium | celatum | -4.39 ± 1.40 | 0.029 | 0.00E+0 (0.00E+0 - 2.66E-4) | | 0.00E+0 (0.00E+0 - 6.77E-5) | |
| Firmicutes | Clostridia | Clostridiales | Clostridiaceae | Clostridium | baratii | -3.79 ± 1.23 | 0.032 | 6.26E-5 (0.00E+0 - 5.75E-4) | | 0.00E+0 (0.00E+0 - 1.57E-4) | |
| Bacteroidetes | Bacteroidia | Bacteroidales | Bacteroidaceae | Bacteroides | fragilis | -3.74 ± 0.96 | 0.003 | 4.38E-3 (7.43E-4 - 1.91E-2) | | 5.08E-3 (1.39E-3 - 9.73E-3) | |
| Firmicutes | Bacilli | Lactobacillales | Streptococcaceae | Lactococcus | lactis | -3.58 ± 0.93 | 0.003 | 4.51E-3 (3.76E-4 - 6.93E-2) | | 1.70E-3 (7.15E-4 - 1.37E-2) | |
| Proteobacteria | Gammaproteobacteria | Enterobacterales | Enterobacteriaceae | Citrobacter | braakii | -3.26 ± 0.93 | 0.011 | 4.38E-3 (7.43E-4 - 1.91E-2) | | 1.77E-4 (6.40E-5 - 5.10E-4) | |
| Proteobacteria | Gammaproteobacteria | Enterobacterales | Enterobacteriaceae | Citrobacter | sp MGH100 | -3.10 ± 1.05 | 0.047 | 6.53E-5 (0.00E+0 - 2.31E-4) | | 0.00E+0 (0.00E+0 - 0.00E+0) | |
| Firmicutes | Bacilli | Lactobacillales | Streptococcaceae | Streptococcus | orisratti | -2.86 ± 0.92 | 0.031 | 4.34E-4 (2.28E-5 - 2.59E-3) | | 6.79E-5 (0.00E+0 - 5.20E-4) | |
| Firmicutes | Clostridia | Clostridiales | Clostridiaceae | Clostridium | disporicum | -2.80 ± 0.90 | 0.032 | 3.76E-4 (6.44E-5 - 1.53E-3) | | 1.20E-4 (0.00E+0 - 7.73E-4) | |
| Firmicutes | Bacilli | Lactobacillales | Streptococcaceae | Lactococcus | piscium | -2.75 ± 0.82 | 0.016 | 4.06E-4 (1.26E-4 - 2.61E-3) | | 1.36E-4 (0.00E+0 - 4.22E-4) | |
| Bacteroidetes | Bacteroidia | Bacteroidales | Bacteroidaceae | Bacteroides | ND | -2.52 ± 0.78 | 0.021 | 2.69E-1 (3.21E-2 - 1.65E+0) | | 1.29E-1 (2.49E-2 - 6.28E-1) | |
| Bacteroidetes | Bacteroidia | Bacteroidales | Bacteroidaceae | Bacteroides | uniformis | -2.39 ± 0.81 | 0.047 | 8.18E-4 (1.28E-4 - 1.19E-2) | | 8.03E-4 (0.00E+0 - 2.36E-3) | |

* Log_2_FC > 1 represents 2 fold-change at week 4 or 6 compared to week 0

** P values were adjusted using the false discovery rate

FC: fold change, ND: no data (unknown), NS: not significant

**Supplemental Table 4B.** Species of gut bacteria with significant increase or decrease in the differential abundance analysis (|fold change| ≥ 2 and adjusted p < 0.05) at week 4 relative to week 0 in the placebo group (n=19). Species in bold were present in the synbiotic supplement.

| **Phylum** | **Class** | **Order** | **Family** | **Genus** | **Species** | **DESeq2 results**  **week 4/week 0** | | **Relative abundance, in %** | | | |
| --- | --- | --- | --- | --- | --- | --- | --- | --- | --- | --- | --- |
|  |  |  |  |  |  | **Log 2 FC***  **Mean ± SE** | **Adjusted p**** | **Week 0**  **Median (IQR)** | | **Week 4**  **Median (IQR)** | |
| **Increased at week 4** | | | | | | | | | | | |
| Actinobacteria | Actinobacteria | Propionibacteriales | Propionibacteriaceae | Propionibacterium | freudenreichii | 5.01 ± 1.38 | 0.012 | 0.00E+0 (0.00E+0 - 0.00E+0) | | 7.83E-5 (0.00E+0 - 3.57E-4) | |
| Bacteroidetes | Bacteroidia | Bacteroidales | Prevotellaceae | Prevotella | copri | 4.94 ± 1.13 | 0.001 | 7.05E-4 (1.16E-4 - 3.36E-3) | | 6.20E-4 (8.13E-5 - 1.63E-2) | |
| Firmicutes | Bacilli | Lactobacillales | Lactobacillaceae | Lactobacillus | algidus | 4.90 ± 1.16 | 0.001 | 7.76E-4 (1.10E-4 - 7.37E-3) | | 9.75E-4 (4.14E-4 - 8.01E-3) | |
| Firmicutes | Bacilli | Lactobacillales | Enterococcaceae | Enterococcus | sp HMSC05C03 | 4.37 ± 1.33 | 0.021 | 0.00E+0 (0.00E+0 - 1.88E-4) | | 3.92E-5 (0.00E+0 - 1.82E-3) | |
| Firmicutes | Bacilli | Lactobacillales | Streptococcaceae | Lactococcus | garvieae | 4.22 ± 1.09 | 0.005 | 5.43E-4 (0.00E+0 - 1.73E-3) | | 2.76E-4 (1.11E-4 - 2.34E-3) | |
| Firmicutes | Bacilli | Lactobacillales | Lactobacillaceae | Lactobacillus | sakei | 4.18 ± 1.18 | 0.012 | 2.16E-3 (2.76E-4 - 9.08E-3) | | 1.63E-3 (3.68E-4 - 6.68E-3) | |
| Firmicutes | Bacilli | Lactobacillales | Leuconostocaceae | Leuconostoc | gelidum | 3.45 ± 1.07 | 0.024 | 1.29E-3 (7.74E-5 - 3.24E-2) | | 1.96E-3 (6.18E-4 - 6.94E-3) | |
| Proteobacteria | Gammaproteobacteria | Enterobacterales | Enterobacteriaceae | Citrobacter | freundii | 3.19 ± 0.91 | 0.012 | 1.20E-3 (2.83E-5 - 5.22E-3) | | 1.19E-3 (8.66E-4 - 1.64E-2) | |
| Firmicutes | Bacilli | Lactobacillales | Lactobacillaceae | Lactobacillus | curvatus | 3.04 ± 0.94 | 0.024 | 2.57E-4 (1.80E-5 - 6.29E-4) | | 4.11E-4 (7.41E-5 - 1.55E-3) | |
| **Decreased at week 4** | | | | | | | | | | | |
| **Firmicutes** | **Actinobacteria** | **Bifidobacteriales** | **Bifidobacteriaceae** | **Bifidobacterium** | **animalis** | -8.50 ± 1.32 | < 0.001 | 9.48E-4 (7.55E-5 - 2.74E-3) | | 7.83E-5 (0.00E+0 - 5.35E-4) | |
| Firmicutes | Bacilli | Lactobacillales | Leuconostocaceae | Weissella | ND | -7.58 ± 1.37 | < 0.001 | 1.27E-4 (0.00E+0 - 1.22E-3) | | 1.05E-4 (0.00E+0 - 1.94E-4) | |
| Firmicutes | Bacilli | Lactobacillales | Streptococcaceae | Streptococcus | sp HMSC10E12 | -7.07 ± 2.02 | 0.012 | 0.00E+0 (0.00E+0 - 8.86E-5) | | 0.00E+0 (0.00E+0 - 3.17E-5) | |
| Firmicutes | Bacilli | Lactobacillales | Leuconostocaceae | Weissella | cibaria | -6.24 ± 1.22 | < 0.001 | 1.27E-4 (0.00E+0 - 2.40E-3) | | 1.57E-4 (4.66E-5 - 6.80E-4) | |
| Firmicutes | Bacilli | Lactobacillales | Streptococcaceae | Streptococcus | anginosus | -4.44 ± 1.25 | 0.012 | 1.91E-4 (0.00E+0 - 1.12E-3) | | 1.08E-4 (0.00E+0 - 2.92E-4) | |
| Firmicutes | Clostridia | Clostridiales | Clostridiaceae | Clostridium | perfringens | -4.29 ± 0.96 | 0.001 | 9.78E-3 (1.16E-3 - 3.71E-1) | | 3.88E-3 (1.59E-3 - 2.62E-2) | |
| Firmicutes | Bacilli | Lactobacillales | Leuconostocaceae | Weissella | confusa | -3.86 ± 1.17 | 0.021 | 3.29E-4 (0.00E+0 - 3.00E-3) | | 5.40E-5 (0.00E+0 - 3.66E-4) | |
| Firmicutes | Bacilli | Lactobacillales | Streptococcaceae | Streptococcus | agalactiae | -3.81 ± 1.07 | 0.012 | 2.33E-4 (4.94E-5 - 5.31E-4) | | 0.00E+0 (0.00E+0 - 8.36E-5) | |
| Firmicutes | Bacilli | Lactobacillales | Enterococcaceae | Enterococcus | hirae | -3.48 ± 0.87 | 0.003 | 4.68E-4 (1.46E-4 - 8.12E-3) | | 4.69E-4 (1.82E-4 - 2.13E-3) | |
| Firmicutes | Bacilli | Lactobacillales | Streptococcaceae | Lactococcus | piscium | -3.23 ± 1.01 | 0.026 | 1.65E-4 (0.00E+0 - 3.82E-3) | | 3.35E-4 (1.24E-4 - 6.27E-4) | |
| Firmicutes | Bacilli | Lactobacillales | Enterococcaceae | Enterococcus | faecalis | -3.06 ± 1.01 | 0.045 | 8.52E-3 (1.06E-3 - 1.18E-1) | | 0.00E+0 (0.00E+0 - 0.00E+0) | |
| Firmicutes | Bacilli | Lactobacillales | Streptococcaceae | Streptococcus | suis | -2.93 ± 0.84 | 0.012 | 1.13E-3 (1.52E-4 - 6.64E-3) | | 7.13E-4 (4.24E-4 - 3.84E-3) | |

* Log_2_FC > 1 represents 2 fold-change at week 4 or 6 compared to week 0

** P values were adjusted using the false discovery rate

FC: fold change, ND: no data (unknown), NS: not significant

**Supplemental Table 4C.** Species of gut bacteria with significant increase or decrease in their abundance (|fold change| ≥ 2 and adjusted p < 0.05) at week 6 relative to week 0 in the synbiotic group (n=21). Species in bold were present in the synbiotic supplement.

| **Phylum** | **Class** | **Order** | **Family** | **Genus** | **Species** | **DESeq2 results**  **week 6/week 0** | | **Relative abundance, in %** | | | |
| --- | --- | --- | --- | --- | --- | --- | --- | --- | --- | --- | --- |
|  |  |  |  |  |  | **Log 2 FC***  **Mean ± SE** | **Adjusted p**** | **Week 0**  **Median (IQR)** | | **Week 6**  **Median (IQR)** | |
| **Increased at week 6** | | | | | | | | | | | |
| Bacteroidetes | Bacteroidia | Bacteroidales | Prevotellaceae | Prevotella | copri | 4.00 ± 1.15 | 0.023 | 8.94E-4 (2.12E-4 - 7.23E-3) | | 6.90E-3 (7.03E-4 - 4.06E-2) | |
| Firmicutes | Clostridia | Clostridiales | Clostridiaceae | Clostridium | colicanis | 3.87 ± 1.24 | 0.045 | 2.81E-4 (0.00E+0 - 8.22E-4) | | 7.06E-5 (0.00E+0 - 2.79E-3) | |
| Firmicutes | Bacilli | Lactobacillales | Lactobacillaceae | Lactobacillus | amylovorus | 3.62 ± 1.16 | 0.045 | 7.74E-5 (0.00E+0 - 1.27E-3) | | 1.01E-4 (0.00E+0 - 1.70E-3) | |
| Firmicutes | Erysipelotrichia | Erysipelotrichales | Erysipelotrichaceae | Erysipelatoclostridium | spiroforme | 3.58 ± 0.91 | 0.008 | 1.09E-4 (0.00E+0 - 4.04E-4) | | 1.41E-4 (5.00E-5 - 1.11E-3) | |
| **Firmicutes** | **Bacilli** | **Lactobacillales** | **Lactobacillaceae** | **Lactobacillus** | **acidophilus** | 3.48 ± 1.07 | 0.037 | 2.96E-3 (9.22E-4 - 2.49E-2) | | 2.94E-3 (1.17E-4 - 3.96E-2) | |
| Firmicutes | Bacilli | Lactobacillales | Streptococcaceae | Streptococcus | thermophilus | 2.90 ± 0.95 | 0.048 | 0.00E+0 (0.00E+0 - 0.00E+0) | | 1.22E-3 (5.09E-4 - 1.43E-2) | |
| Firmicutes | Erysipelotrichia | Erysipelotrichales | Erysipelotrichaceae | Holdemanella | biformis | 2.82 ± 0.92 | 0.048 | 9.81E-4 (3.49E-4 - 4.97E-3) | | 3.22E-3 (9.07E-4 - 1.39E-1) | |
| **Decreased at week 6** | | | | | | | | | | | |
| Firmicutes | Bacilli | Bacillales | Planococcaceae | Kurthia | sp Dielmo | -7.76 ± 2.07 | 0.012 | 0.00E+0 (0.00E+0 - 6.79E-5) | | 0.00E+0 (0.00E+0 - 0.00E+0) | |
| Proteobacteria | Gammaproteobacteria | Enterobacterales | Hafniaceae | Hafnia | ND | -5.75 ± 1.85 | 0.045 | 0.00E+0 (0.00E+0 - 9.27E-5) | | 0.00E+0 (0.00E+0 - 0.00E+0) | |
| Firmicutes | Bacilli | Lactobacillales | Enterococcaceae | Enterococcus | canintestini | -5.61 ± 1.17 | 0.001 | 2.18E-4 (0.00E+0 - 6.38E-3) | | 7.73E-5 (0.00E+0 - 3.57E-4) | |
| Actinobacteria | Actinobacteria | Micrococcales | Micrococcaceae | Arthrobacter | ND | -5.52 ± 1.42 | 0.008 | 5.80E-5 (0.00E+0 - 2.64E-4) | | 4.21E-5 (0.00E+0 - 5.41E-4) | |
| Proteobacteria | Gammaproteobacteria | Enterobacterales | Enterobacteriaceae | Raoultella | planticola | -5.37 ± 1.71 | 0.045 | 0.00E+0 (0.00E+0 - 1.52E-4) | | 0.00E+0 (0.00E+0 - 0.00E+0) | |
| Firmicutes | Bacilli | Lactobacillales | Enterococcaceae | Enterococcus | sp CR-Ec1 | -4.73 ± 1.27 | 0.012 | 5.59E-5 (0.00E+0 - 3.15E-3) | | 0.00E+0 (0.00E+0 - 3.04E-4) | |
| Firmicutes | Bacilli | Lactobacillales | Enterococcaceae | Enterococcus | sp 6D12 DIV0197 | -4.31 ± 1.16 | 0.012 | 2.18E-4 (2.79E-5 - 1.33E-2) | | 0.00E+0 (0.00E+0 - 1.35E-3) | |
| Firmicutes | Bacilli | Lactobacillales | Enterococcaceae | Enterococcus | sp 5B3 DIV0040 | -4.20 ± 1.29 | 0.037 | 2.24E-4 (0.00E+0 - 1.01E-2) | | 0.00E+0 (0.00E+0 - 1.05E-3) | |
| Bacteroidetes | Bacteroidia | Bacteroidales | Bacteroidaceae | Bacteroides | ovatus | -4.19 ± 0.88 | 0.001 | 0.00E+0 (0.00E+0 - 0.00E+0) | | 1.05E-3 (5.71E-4 - 7.26E-3) | |
| Firmicutes | Bacilli | Lactobacillales | Enterococcaceae | Enterococcus | casseliflavus | -4.16 ± 1.02 | 0.008 | 2.29E-3 (2.89E-4 - 1.39E-1) | | 3.39E-4 (7.13E-5 - 1.21E-2) | |
| Firmicutes | Bacilli | Lactobacillales | Enterococcaceae | Enterococcus | sp 4E1 DIV0656 | -4.16 ± 1.35 | 0.046 | 7.64E-5 (0.00E+0 - 3.59E-3) | | 0.00E+0 (0.00E+0 - 2.60E-4) | |
| Firmicutes | Bacilli | Lactobacillales | Enterococcaceae | Enterococcus | sp 3C8 DIV0646 | -4.10 ± 1.30 | 0.045 | 1.12E-4 (0.00E+0 - 1.25E-3) | | 0.00E+0 (0.00E+0 - 8.68E-5) | |
| Proteobacteria | Gammaproteobacteria | Enterobacterales | Enterobacteriaceae | Klebsiella | quasipneumoniae | -3.92 ± 1.00 | 0.008 | 9.29E-4 (1.71E-4 -1.16E-2) | | 1.84E-4 (0.00E+0 - 1.03E-3) | |
| Firmicutes | Bacilli | Lactobacillales | Enterococcaceae | Enterococcus | sp FDAARGOS 163 | -3.84 ± 1.24 | 0.045 | 4.17E-4 (0.00E+0 - 5.80E-3) | | 0.00E+0 (0.00E+0 - 6.14E-4) | |
| Firmicutes | Clostridia | Clostridiales | Clostridiaceae | Clostridium | nigeriense | -3.70 ± 1.14 | 0.039 | 6.70E-5 (0.00E+0 - 1.64E-4) | | 0.00E+0 (0.00E+0 - 4.34E-5) | |
| Proteobacteria | Gammaproteobacteria | Enterobacterales | Enterobacteriaceae | Klebsiella | ND | -3.54 ± 0.85 | 0.006 | 7.31E-3 (2.28E-3 - 1.51E-1) | | 4.97E-3 (1.57E-3 - 1.98E-2) | |
| Bacteroidetes | Bacteroidia | Bacteroidales | Tannerellaceae | Parabacteroides | ND | -3.48 ± 0.97 | 0.017 | 3.82E-4 (0.00E+0 - 2.38E-3) | | 1.61E-4 (7.13E-5 - 4.64E-4) | |
| Firmicutes | Bacilli | Lactobacillales | Enterococcaceae | Enterococcus | mundtii | -3.37 ± 0.86 | 0.008 | 4.64E-4 (1.19E-4 - 2.74E-3) | | 2.32E-4 ( 7.13E-5 - 7.36E-4) | |
| Actinobacteria | Actinobacteria | Corynebacteriales | Mycobacteriaceae | Mycobacterium | ND | -3.32 ± 1.04 | 0.043 | 6.70E-5 (0.00E+0 - 4.54E-4) | | 0.00E+0 (0.00E+0 - 6.22E-5) | |
| Firmicutes | Clostridia | Clostridiales | Lachnospiraceae | Anaerostipes | ND | -3.27 ± 0.91 | 0.017 | 3.48E-4 (2.75E-5 - 1.31E-3) | | 2.19E-4 (8.06E-5 - 4.89E-4) | |
| Proteobacteria | Gammaproteobacteria | Enterobacterales | Enterobacteriaceae | Citrobacter | ND | -3.11 ± 0.94 | 0.037 | 4.82E-3 (1.07E-3 - 2.13E-2) | | 1.98E-3 (1.43E-4 - 1.14E-2) | |
| Bacteroidetes | Bacteroidia | Bacteroidales | Bacteroidaceae | Bacteroides | dorei | -3.01 ± 0.87 | 0.023 | 1.15E-3 (9.67E-5 - 7.22E-3) | | 2.95E-4 (7.13E-5 - 8.07E-4) | |
| Firmicutes | Bacilli | Lactobacillales | Streptococcaceae | Lactococcus | piscium | -2.69 ± 0.80 | 0.032 | 4.06E-4 (1.26E-4 - 2.61E-3) | | 2.82E-4 (6.22E-5 - 7.99E-4) | |

* Log_2_FC > 1 represents 2 fold-change at week 4 or 6 compared to week 0

** P values were adjusted using the false discovery rate

FC: fold change, ND: no data (unknown), NS: not significant
