# Supplementary material for: The microbiota of healthy dogs demonstrates individualized responses to synbiotic supplementation in a randomized controlled trial
Source: Anim Microbiome. 2021 May 10;3:36. doi: 10.1186/s42523-021-00098-0 (PMC8111948; doi:10.1186/s42523-021-00098-0)
Supplement: Supplementary file 6 — Additional file 6: Table S5. Relative abundances (as % of reads in the synbiotic supplement) for species with significantly increased abundances in the stool at week 4 in the synbiotic group. Only species not added to the supplement are reported and relative abundances were averaged from two samples. [file 42523_2021_98_MOESM6_ESM.docx]

**Supplemental Table 5.** Relative abundances (as % of reads in the synbiotic supplement) for species with significantly increased abundances in the stool at week 4 in the synbiotic group. Only species not added to the supplement are reported and relative abundances were averaged from two samples.

| **Species** | **Relative abundance, in %** |
| --- | --- |
| Enterococcus durans  Enterococcus malodoratus  Enterococcus pseudoavium  Enterococcus sp 10A9 DIV0425  Enterococcus sp 3G1 DIV0629  Enterococcus sp HMSC034B11  Enterococcus sp HMSC035C10  Enterococcus sp HMSC060D09  Enterococcus sp HMSC060E05  Enterococcus sp HMSC061C05  Enterococcus sp HMSC063C12  Enterococcus sp HMSC063D12  Enterococcus sp HMSC063H10  Enterococcus sp HMSC065H03  Enterococcus sp HMSC067C01  Enterococcus sp HMSC072D11  Enterococcus sp HMSC072F02  Enterococcus sp HMSC076D08  Enterococcus sp HMSC076E04  Enterococcus sp HMSC077E07  Enterococcus sp HMSC34G12  Enterococcus villorum  Erysipelatoclostridium spiroforme  Lactobacillus frumenti  Lactobacillus hominis  Lactobacillus ingluviei  Lactobacillus intestinalis  Lactobacillus murinus  Lactobacillus plantarum  Lactobacillus salivarius  Lactobacillus sp ASF360  Lactobacillus sp HMSC24D01  Lactobacillus sp UMNPBX3  Lactobacillus taiwanensis  Lactobacillus vaginalis  unknown Lactobacillus  unknown Pediococcus  unknown Facklamia | 0.014  0.001  <0.001  0.005  0.019  0.001  0.023  <0.001  0.001  0.149  <0.001  0.390  0.024  0.001  0.152  0.004  0.076  <0.001  0.223  0.001  0.008  0.002  not detected  not detected  <0.001  not detected  <0.001  not detected  3.145  <0.001  <0.001  0.021  not detected  <0.001  not detected  9.243  0.022  0.002 |
