# Supplementary material for: The microbiota of healthy dogs demonstrates individualized responses to synbiotic supplementation in a randomized controlled trial
Source: Anim Microbiome. 2021 May 10;3:36. doi: 10.1186/s42523-021-00098-0 (PMC8111948; doi:10.1186/s42523-021-00098-0)
Supplement: Supplementary file 7 — Additional file 7: Table S6. Baseline subject characteristics of high- (HR), mid- (MR), and low-responders (LR) in the synbiotics group. [file 42523_2021_98_MOESM7_ESM.docx]

# **Supplemental Table 6.** Baseline subject characteristics of high- (HR), mid- (MR), and low-responders (LR) in the synbiotics group.

| **Feature** | **HR (n = 8)** | **MR (n = 7)** | **LR**  **(n = 8)** | **p value*** |
| --- | --- | --- | --- | --- |
| Age, in years  Male  Spayed or neutered  Body condition score  1-3  4-5  6  Ideal body weight, in kg  Physical activity level  Normal  Active  Very active | 3.9 ± 2.2  5 (63%)  8 (100%)  1 (13%)  6 (75%)  1 (13%)  10.7 ± 8.5  5 (63%)  2 (25%)  1 (13%) | 5.3 ± 3.3  4 (57%)  7 (100%)  1 (14%)  6 (86%)  0 (0%)  12.9 ± 10.2  5 (71%)  1 (14%)  1 (14%) | 5.8 ± 3.7  5 (63%)  6 (75%)  0 (0%)  7 (88%)  1 (13%)  8.2 ± 4.7  5 (63%)  2 (25%)  1 (13%) | 0.572  1  0.304  1  0.693  1 |

* Kruskal-Wallis test for continuous variables and Fisher’s exact test for categorical variables.
