# Supplementary material for: The microbiota of healthy dogs demonstrates individualized responses to synbiotic supplementation in a randomized controlled trial
Source: Anim Microbiome. 2021 May 10;3:36. doi: 10.1186/s42523-021-00098-0 (PMC8111948; doi:10.1186/s42523-021-00098-0)
Supplement: Supplementary file 8 — Additional file 8: Table S7. Median (IQR) of gut microbial evenness, richness, and α-diversity indices among tertiles of responders (n = 23) at sequencing coverage of 380,000 reads. [file 42523_2021_98_MOESM8_ESM.docx]

**Supplemental Table 7.** Median (IQR) of gut microbial evenness, richness, and 𝛂-diversity indices among tertiles of responders (n = 23) at sequencing coverage of 380,000 reads.*

| **Measure** | **Week** | **LR**  **(n=8)** | | **MR**  **(n=7)** | | **HR**  **(n=8)** | | **p**** | **p***** |
| --- | --- | --- | --- | --- | --- | --- | --- | --- | --- |
|  |  |  |  |  |  |  |  |  |  |
| Evenness | 0  4  6 | 0.59^a^ (0.52-0.60)  0.54 (0.53-0.58)  0.57 (0.55-0.60) | | 0.64^b^ (0.62-0.65)  0.61 (0.58-0.65)  0.61 (0.59-0.62) | | 0.58^a^ (0.51-0.59)  0.56 (0.52-0.60)  0.48 (0.46-0.56) | | 0.022  0.302  0.099 | NA  0.769  0.768 |
| Richness | 0  4  6 | 675 (605-772)  663 (607-775)  645 (613-681) | | 728 (698-847)  707 (690-781)  753 (677-868) | | 609 (508-708)  635 (612-781)  660 (565-785) | | 0.067  0.308  0.210 | NA  0.431  0.406 |
| Shannon’s diversity index | 0  4  6 | 2.73^a^ (2.41-2.81)  2.53 (2.48-2.72)  2.64 (2.56-2.80) | | 2.96^b^ (2.88-3.04)  2.85 (2.69-3.04)  2.84 (2.74-2.89) | | 2.71^a^ (2.36-2.75)  2.59 (2.41-2.79)  2.24 (2.16-2.61) | | 0.022  0.302  0.099 | NA  0.769  0.768 |
| Simpson’s diversity index | 0  4  6 | 2.73^a^ (2.41-2.81)  2.53 (2.48-2.72)  2.64 (2.56-2.80) | | 2.96^b^ (2.88-3.04)  2.85 (2.69-3.04)  2.84 (2.74-2.89) | | 2.71^a^ (2.36-2.75)  2.59 (2.41-2.79)  2.24 (2.16-2.61) | | 0.022  0.302  0.099 | NA  0.769  0.768 |

LR: low responder, MR: mid responder, HR: high responder

* Data at week 6 are available for 21 subjects: 7 in LR, 6 in MR, and 8 in HR.

** Kruskall-Wallis test comparing measures at each timepoint between LR, MR, and HR. Medians that do not share the same superscript are statistically different as shown by Wilcoxon rank sum test with FDR adjustment for pairwise comparisons.

*** Kruskall-Wallis test comparing changes in each measure from week 0 among LR, MR, and HR.
