# Supplementary material for: The microbiota of healthy dogs demonstrates individualized responses to synbiotic supplementation in a randomized controlled trial
Source: Anim Microbiome. 2021 May 10;3:36. doi: 10.1186/s42523-021-00098-0 (PMC8111948; doi:10.1186/s42523-021-00098-0)
Supplement: Supplementary file 9 — Additional file 9: Table S8A. Bacterial species that were significantly different in the differential abundance analysis (|fold change| ≥ 2 and p < 0.05) between high-responders (HR, n = 8) and low-responders (LR, n = 8) at baseline. Species in bold were present in the synbiotic supplement. Table S8B. Bacterial species that were significantly different in the differential abundance analysis (|fold change| ≥ 2 and p < 0.05) between high-responders (HR, n = 8) and low-responders (LR, n = 8) at week 4. Species in bold were present in the synbiotic supplement. [file 42523_2021_98_MOESM9_ESM.docx]

# Supplemental Table 8A**.** Bacterial species that were significantly different in the differential abundance analysis (|fold change| ≥ 2 and p < 0.05) between high-responders (HR, n = 8) and low-responders (LR, n = 8) at baseline. Species in bold were present in the synbiotic supplement.

| **Phylum** | **Class** | **Order** | **Family** | **Genus** | **Species** | **DESeq2 results**  **HR/LR** | | **Relative abundance, in %** | | | |
| --- | --- | --- | --- | --- | --- | --- | --- | --- | --- | --- | --- |
|  |  |  |  |  |  | **Log 2 FC***  **mean ± SE** | **Adjusted p**** | **HR**  **Median (IQR)** | | **LR**  **Median (IQR)** | |
| **Higher in HR at baseline** | | | | | | | | | | | |
| Proteobacteria | Gammaproteobacteria | Enterobacterales | Enterobacteriaceae | Prevotella | copri | 6.76 ± 1.88 | 0.007 | 1.48E-3 (4.86E-4 - 5.35E-1) | | 7.41E-4 (1.30E-4 - 2.85E-3) | |
| Proteobacteria | Gammaproteobacteria | Enterobacterales | Enterobacteriaceae | Escherichia | sp KTE172 | 5.44 ± 1.39 | 0.003 | 6.62E-3 (4.96E-4 - 9.53E-2) | | 5.48E-4 (1.85E-4 - 3.27E-3) | |
| Bacteroidetes | Bacteroidia | Bacteroidales | Prevotellaceae | Escherichia | albertii | 4.92 ± 1.33 | 0.005 | 5.43E-2 (1.37E-2 - 3.69E-1) | | 3.71E-3 (7.46E-4 - 3.00E-2) | |
| Proteobacteria | Gammaproteobacteria | Enterobacterales | Enterobacteriaceae | Escherichia | KTE159 | 4.48 ± 1.60 | 0.044 | 1.12E-3 (7.87E-4 - 1.08E-2) | | 2.90E-5 (0.00E+0 - 8.79E-4) | |
| Proteobacteria | Gammaproteobacteria | Enterobacterales | Enterobacteriaceae | Shigella | dysenteriae | 4.27 ± 1.34 | 0.019 | 4.11E-2 (9.36E-3 - 2.80E-1) | | 4.02E-3 (1.14E-3 - 3.67E-2) | |
| Proteobacteria | Betaproteobacteria | Burkholderiales | Alcaligenaceae | Achromobacter | sp ATCC35328 | 4.22 ± 1.45 | 0.035 | 3.59E-3 (8.18E-4 - 2.10E-2) | | 4.69E-4 (1.67E-4 - 2.96E-3) | |
| Proteobacteria | Gammaproteobacteria | Enterobacterales | Enterobacteriaceae | Escherichia | coli | 3.91 ± 1.28 | 0.026 | 5.60E-1 (3.66E-1 - 5.46E+0) | | 6.98E-2 (1.78E-2 - 5.89E-1) | |
| Proteobacteria | Gammaproteobacteria | ND | ND | ND | ND | 3.88 ± 1.19 | 0.016 | 3.60E-3 (1.22E-3 - 1.39E-2) | | 6.79E-4 (3.31E-4 - 2.20E-3) | |
| Proteobacteria | Gammaproteobacteria | Enterobacterales | Enterobacteriaceae | Shigella | flexneri | 3.85 ± 1.36 | 0.042 | 3.48E-2 (1.36E-2 - 4.30E-1) | | 4.49E-3 (1.14E-3 - 5.61E-2) | |
| Proteobacteria | Gammaproteobacteria | Enterobacterales | Enterobacteriaceae | Shigella | sonnei | 3.76 ± 1.35 | 0.044 | 3.21E-2 (8.86E-3 - 2.35E-1) | | 4.65E-3 (1.69E-3 - 4.33E-2) | |
| Proteobacteria | Gammaproteobacteria | Enterobacterales | Enterobacteriaceae | Escherichia | ND | 3.75 ± 1.25 | 0.031 | 1.01E+0 (5.06E-1 - 5.36E+0) | | 9.10E-2 (1.78E-2 - 2.90E-1) | |
| **Lower in HR at baseline** | | | | | | | | | | | |
| Firmicutes | Bacilli | Lactobacillales | Enterococcaceae | Enterococcus | sulfureus | -26.66 ± 2.86 | < 0.001 | 0.00E+0 (0.00E+0 - 0.00E+0) | | 0.00E+0 (0.00E+0 - 4.83E-3) | |
| Firmicutes | Bacilli | Lactobacillales | Lactobacillaceae | Lactobacillus | sakei | -9.75 ± 1.70 | < 0.001 | 5.51E-4 (1.85E-4 - 1.25E-3) | | 1.57E-2 (2.26E-3 - 3.37E-1) | |
| Firmicutes | Bacilli | Lactobacillales | Lactobacillaceae | ND | ND | -9.13 ± 1.84 | < 0.001 | 0.00E+0 (0.00E+0 - 8.25E-5) | | 2.15E-3 (1.35E-4 - 1.17E-2) | |
| Firmicutes | Bacilli | Lactobacillales | Leuconostocaceae | ND | ND | -8.96 ± 2.33 | 0.004 | 0.00E+0 (0.00E+0 - 0.00E+0) | | 4.32E-4 (0.00E+0 - 1.36E-2) | |
| Firmicutes | Bacilli | Lactobacillales | Enterococcaceae | Enterococcus | sp HMSC061C05 | -8.55 ± 2.05 | 0.002 | 0.00E+0 (0.00E+0 - 5.05E-5) | | 1.42E-3 (0.00E+0 - 8.84E-3) | |
| Firmicutes | Bacilli | Lactobacillales | Enterococcaceae | Enterococcus | sp HMSC069A01 | -8.04 ± 1.95 | 0.002 | 0.00E+0 (0.00E+0 - 1.83E-5) | | 5.88E-4 (1.65E-4 - 4.10E-3) | |
| Firmicutes | Bacilli | Lactobacillales | Leuconostocaceae | Leuconostoc | ND | -7.98 ± 1.65 | < 0.001 | 0.00E+0 (0.00E+0 - 8.81E-5) | | 1.14E-3 (1.09E-4 - 1.60E-2) | |
| Firmicutes | Bacilli | Lactobacillales | Leuconostocaceae | Leuconostoc | gelidum | -7.49 ± 1.40 | < 0.001 | 6.82E-4 (2.23E-4 - 1.52E-3) | | 4.28E-3 (1.37E-3 - 5.09E-1) | |
| Firmicutes | Bacilli | Lactobacillales | Enterococcaceae | Enterococcus | sp HMSC076E04 | -7.42 ± 1.96 | 0.004 | 0.00E+0 (0.00E+0 - 8.25E-5) | | 1.16E-3 (4.19E-5 - 1.09E-2) | |
| Firmicutes | Bacilli | Lactobacillales | Enterococcaceae | Enterococcus | sp HMSC067C01 | -7.41 ± 1.83 | 0.002 | 0.00E+0 (0.00E+0 - 1.45E-4) | | 3.39E-3 (4.31E-4 - 1.27E-2) | |
| Firmicutes | Bacilli | Lactobacillales | Lactobacillaceae | Lactobacillus | backii | -7.17 ± 2.29 | 0.021 | 0.00E+0 (0.00E+0 - 0.00E+0) | | 1.92E-4 (3.72E-5 - 2.35E-3) | |
| Firmicutes | Bacilli | Lactobacillales | Streptococcaceae | Streptococcus | thermophilus | -6.95 ± 1.64 | 0.002 | 1.32E-4 (7.81E-5 - 1.03E-3) | | 9.92E-3 (3.09E-4 - 6.70E-2) | |
| Firmicutes | Bacilli | Lactobacillales | Streptococcaceae | Streptococcus | macedonicus | -6.94 ± 1.63 | 0.002 | 1.03E-4 (0.00E+0 - 3.76E-4) | | 6.45E-3 (6.30E-4 - 4.65E-2) | |
| Firmicutes | Bacilli | Lactobacillales | Enterococcaceae | Enterococcus | sp HMSC063D12 | -6.94 ± 1.69 | 0.002 | 9.87E-5 (0.00E+0 - 1.94E-4) | | 2.07E-3 (2.51E-4 - 1.53E-2) | |
| Firmicutes | Bacilli | Lactobacillales | Leuconostocaceae | Leuconostoc | carnosum | -6.68 ± 1.93 | 0.010 | 0.00E+0 (0.00E+0 - 4.99E-5) | | 9.67E-5 (0.00E+0 - 5.26E-3) | |
| Firmicutes | Bacilli | Lactobacillales | Leuconostocaceae | Leuconostoc | lactis | -6.63 ± 2.30 | 0.036 | 0.00E+0 (0.00E+0 - 0.00E+0) | | 2.90E-4 (0.00E+0 - 1.51E-3) | |
| Firmicutes | Bacilli | Lactobacillales | Enterococcaceae | Enterococcus | sp 3G1 DIV0629 | -6.33 ± 1.95 | 0.017 | 0.00E+0 (0.00E+0 - 8.25E-5) | | 3.43E-4 (0.00E+0 - 2.66E-3) | |
| Firmicutes | Bacilli | Lactobacillales | Enterococcaceae | Enterococcus | sp HMSC072F02 | -6.27 ± 1.99 | 0.020 | 0.00E+0 (0.00E+0 - 6.71E-5) | | 1.51E-3 (2.46E-4 - 5.92E-3) | |
| Firmicutes | Bacilli | Lactobacillales | Leuconostocaceae | Leuconostoc | mesenteroides | -6.23 ± 1.68 | 0.005 | 4.47E-5 (0.00E+0 - 2.24E-4) | | 1.29E-3 (8.25E-4 - 1.31E-2) | |
| Firmicutes | Bacilli | Lactobacillales | Enterococcaceae | Enterococcus | mundtii | -6.10 ± 1.51 | 0.002 | 8.47E-5 (0.00E+0 - 1.38E-4) | | 9.99E-4 (4.20E-4 - 3.00E-2) | |
| Firmicutes | Bacilli | Lactobacillales | Streptococcaceae | Streptococcus | ND | -6.08 ± 1.61 | 0.004 | 3.86E-2 (6.26E-3 - 9.29E-2) | | 1.29E+1 (1.24E-1 - 2.68E+1) | |
| Firmicutes | Bacilli | Lactobacillales | Enterococcaceae | Enterococcus | sp 10F3 DIV0382 | -5.96 ± 1.72 | 0.010 | 8.37E-5 (0.00E+0 - 4.92E-4) | | 1.78E-3 (4.78E-4 - 3.13E-2) | |
| Firmicutes | Bacilli | Lactobacillales | Enterococcaceae | Enterococcus | sp HMSC035C10 | -5.95 ± 1.78 | 0.014 | 0.00E+0 (0.00E+0 - 1.12E-5) | | 5.99E-4 (1.24E-4 - 7.67E-4) | |
| Firmicutes | Bacilli | Lactobacillales | Lactobacillaceae | Lactobacillus | ND | -5.91 ± 1.43 | 0.002 | 7.95E-4 (2.04E-4 - 1.88E-3) | | 1.02E-2 (2.01E-3 - 3.39E-2) | |
| Firmicutes | Bacilli | Lactobacillales | Enterococcaceae | Enterococcus | sp 3G6 DIV0642 | -5.76 ± 1.68 | 0.011 | 0.00E+0 (0.00E+0 - 3.16E-4) | | 7.36E-3 (1.10E-3 - 1.26E-2) | |
| Firmicutes | Bacilli | Lactobacillales | Enterococcaceae | Enterococcus | pernyi | -5.75 ± 1.95 | 0.035 | 0.00E+0 (0.00E+0 - 0.00E+0) | | 2.83E-4 (5.42E-5 - 5.20E-4) | |
| Firmicutes | Bacilli | Lactobacillales | Streptococcaceae | Lactococcus | lactis | -5.75 ± 1.64 | 0.009 | 0.00E+0 (0.00E+0 - 0.00E+0) | | 4.69E-2 (2.33E-2 - 5.56E-1) | |
| Firmicutes | Bacilli | Lactobacillales | Enterococcaceae | Enterococcus | durans | -5.75 ± 1.50 | 0.004 | 5.50E-5 (0.00E+0 - 2.12E-4) | | 3.22E-3 (2.13E-3 - 8.81E-3) | |
| Firmicutes | Bacilli | Lactobacillales | Streptococcaceae | Streptococcus | orisratti | -5.54 ± 1.63 | 0.012 | 3.66E-5 (0.00E+0 - 6.61E-4) | | 2.33E-3 (4.02E-4 - 1.69E-2) | |
| Firmicutes | Bacilli | Lactobacillales | Enterococcaceae | Enterococcus | sp FDAARGOS 163 | -5.46 ± 1.66 | 0.015 | 0.00E+0 (0.00E+0 - 1.04E-4) | | 3.21E-3 (6.17E-4 - 6.05E-3) | |
| Firmicutes | Bacilli | Lactobacillales | Leuconostocaceae | Leuconostoc | citreum | -5.43 ± 1.66 | 0.016 | 6.89E-5 (0.00E+0 - 3.63E-4) | | 1.56E-3 (1.28E-4 - 1.16E-2) | |
| Firmicutes | Bacilli | Lactobacillales | Enterococcaceae | Enterococcus | sp HMSC063H10 | -5.34 ± 1.44 | 0.005 | 5.50E-5 (0.00E+0 - 8.21E-5) | | 6.09E-4 (1.42E-4 - 1.40E-3) | |
| Firmicutes | Bacilli | Lactobacillales | Lactobacillaceae | Lactobacillus | curvatus | -5.29 ± 1.38 | 0.004 | 1.00E-4 (0.00E+0 - 2.83E-4) | | 2.25E-3 (3.66E-4 - 1.04E-2) | |
| Firmicutes | Bacilli | Lactobacillales | Enterococcaceae | Enterococcus | ND | -5.03 ± 1.38 | 0.005 | 4.04E-2 (1.86E-2 - 4.73E-1) | | 1.78E+0 (1.18E+0 - 3.56E+0) | |
| Firmicutes | Bacilli | Lactobacillales | Enterococcaceae | Enterococcus | sp 6D12 DIV0197 | -4.71 ± 1.60 | 0.034 | 3.35E-5 (0.00E+0 - 6.85E-4) | | 4.67E-3 (8.68E-4 - 1.22E-2) | |
| Firmicutes | Bacilli | Lactobacillales | Enterococcaceae | Enterococcus | casseliflavus | -4.63 ± 1.55 | 0.031 | 2.84E-4 (2.01E-4 - 4.15E-3) | | 4.29E-2 (5.61E-3 - 1.52E-1) | |
| Firmicutes | Bacilli | Lactobacillales | Enterococcaceae | Enterococcus | sp 8E11 MSG4843 | -4.63 ± 1.58 | 0.035 | 0.00E+0 (0.00E+0 - 2.90E-4) | | 1.38E-3 (3.02E-4 - 2.83E-3) | |
| **Firmicutes** | **Bacilli** | **Lactobacillales** | **Enterococcaceae** | **Enterococcus** | **faecium** | -4.61 ± 1.59 | 0.035 | 1.30E-4 (5.60E-5 - 5.97E-4) | | 4.34E-3 (1.39E-3 - 1.52E-2) | |
| Firmicutes | Bacilli | Lactobacillales | Streptococcaceae | Streptococcus | parauberis | -4.60 ± 1.48 | 0.023 | 8.94E-5 (6.65E-5 - 2.24E-4) | | 9.65E-4 (1.82E-4 - 1.49E-2) | |
| Firmicutes | Bacilli | Lactobacillales | Streptococcaceae | Streptococcus | lutetiensis | -4.48 ± 1.63 | 0.048 | 6.11E-3 (4.93E-4 - 1.69E-2) | | 1.74E-2 (6.20E-3 - 4.40E+0) | |
| Firmicutes | Bacilli | Lactobacillales | Streptococcaceae | Streptococcus | infantarius | -4.42 ± 1.53 | 0.036 | 2.11E-3 (6.16E-4 - 3.63E-3) | | 1.26E-2 (6.22E-3 - 1.19E+0) | |
| Firmicutes | Erysipelotrichia | Erysipelotrichales | Erysipelotrichaceae | Allobaculum | stercoricanis | -4.36 ± 1.50 | 0.035 | 3.70E-4 (2.04E-4 - 2.64E-3) | | 1.02E-3 (2.89E-4 - 8.29E-3) | |
| Firmicutes | Bacilli | Lactobacillales | Streptococcaceae | Streptococcus | equinus | -4.30 ± 1.48 | 0.035 | 6.20E-4 (3.18E-4 - 2.86E-3) | | 1.32E-1 (3.28E-3 - 5.13E-1) | |
| Firmicutes | Bacilli | Lactobacillales | ND | ND | ND | -4.19 ± 1.26 | 0.014 | 8.51E-4 (3.49E-4 - 2.35E-3) | | 9.04E-2 (3.44E-2 - 2.01E-1) | |
| Firmicutes | Bacilli | Lactobacillales | Streptococcaceae | Lactococcus | piscium | -3.97 ± 1.23 | 0.017 | 3.07E-4 (1.16E-4 - 6.85E-4) | | 6.48E-3 (1.03E-3 - 3.05E-2) | |
| Firmicutes | Bacilli | Lactobacillales | Streptococcaceae | ND | ND | -3.87 ± 1.23 | 0.020 | 1.35E-4 (1.00E-4 - 3.02E-4) | | 5.08E-3 (1.37E-3 - 1.70E-2) | |
| Firmicutes | Bacilli | Lactobacillales | Streptococcaceae | Lactococcus | ND | -3.85 ± 1.32 | 0.035 | 3.27E-5 (0.00E+0 - 1.56E-4) | | 3.19E-3 (1.48E-3 - 6.28E-3) | |
| Firmicutes | Bacilli | Lactobacillales | Streptococcaceae | Lactococcus | garvieae | -3.73 ± 1.36 | 0.048 | 6.71E-5 (0.00E+0 - 1.67E-4) | | 3.19E-3 (1.48E-3 - 6.28E-3) | |
| Firmicutes | Bacilli | ND | ND | ND | ND | -3.53 ± 1.27 | 0.046 | 6.39E-4 (1.17E-4 - 3.17E-3) | | 8.50E-3 (3.22E-3 - 5.36E-2) | |
| Firmicutes | Bacilli | Lactobacillales | Lactobacillaceae | Lactobacillus | algidus | -3.35 ± 1.20 | 0.044 | 8.07E-4 (1.36E-4 - 1.07E-3) | | 2.54E-3 (4.81E-4 - 6.68E-3) | |
| Firmicutes | Erysipelotrichia | Erysipelotrichales | Erysipelotrichaceae | Turicibacter | ND | -3.10 ± 1.11 | 0.044 | 8.21E-4 (4.58E-4 - 1.19E-3) | | 3.78E-3 (1.54E-3 - 1.50E-2) | |

* Log_2_FC = 1 represents 2 fold-change of HR:LR

** P values were adjusted using the false discovery rate

FC: fold change (HR/LR), ND: no data (unknown)

#

# Supplemental Table 8B. Bacterial species that were significantly different in the differential abundance analysis (|fold change| ≥ 2 and p < 0.05) between high-responders (HR, n = 8) and low-responders (LR, n = 8) at week 4. Species in bold were present in the synbiotic supplement.

| **Phylum** | **Class** | **Order** | **Family** | **Genus** | **Species** | **DESeq2 results**  **HR/LR** | | **Relative abundance, in %** | | | |
| --- | --- | --- | --- | --- | --- | --- | --- | --- | --- | --- | --- |
|  |  |  |  |  |  | **Log 2 FC***  **mean ± SE** | **Adjusted p**** | **HR**  **Median (IQR)** | | **LR**  **Median (IQR)** | |
| **Higher in HR at week 4** | | | | | | | | | | | |
| Firmicutes | Bacilli | Lactobacillales | Aerococcaceae | Facklamia | ND | 6.68 ± 1.58 | 0.004 | 8.69E-4 (5.71E-4 - 2.12E-3) | | 0.00E+0 (0.00E+0 - 0.00E+0) | |
| Firmicutes | Bacilli | Lactobacillales | Enterococcaceae | Enterococcus | sp HMSC077E07 | 5.17 ± 1.64 | 0.025 | 4.38E-4 (2.15E-4 - 9.93E-4) | | 0.00E+0 (0.00E+0 - 0.00E+0) | |
| Firmicutes | Bacilli | Lactobacillales | Lactobacillaceae | Lactobacillus | sp ASF360 | 4.95 ± 1.39 | 0.015 | 1.29E-3 (6.15E-4 - 4.04E-3) | | 4.38E-5 (0.00E+0 - 1.88E-4) | |
| Firmicutes | Bacilli | Lactobacillales | Enterococcaceae | Enterococcus | sp HMSC34G12 | 4.90 ± 1.42 | 0.015 | 5.80E-3 (4.24E-3 - 1.33E-2) | | 6.73E-5 (0.00E+0 - 1.40E-4) | |
| Firmicutes | Bacilli | Lactobacillales | Enterococcaceae | Enterococcus | sp HMSC072D11 | 4.90 ± 1.55 | 0.025 | 2.41E-3 (1.05E-3 - 4.07E-3) | | 0.00E+0 (0.00E+0 - 1.67E-5) | |
| **Firmicutes** | **Bacilli** | **Lactobacillales** | **Lactobacillaceae** | **Lactobacillus** | **reuteri** | 4.84 ± 1.39 | 0.015 | 5.00E+0 (2.32E+0 - 1.93E+1) | | 1.49E-1 (2.10E-3 - 5.25E-1) | |
| Firmicutes | Erysipelotrichia | Erysipelotrichales | Erysipelotrichaceae | Holdemanella | biformis | 4.82 ± 1.38 | 0.015 | 1.90E-3 (1.32E-3 - 6.49E-2) | | 1.64E-3 (2.98E-4 - 2.81E-3) | |
| Firmicutes | Bacilli | Lactobacillales | Lactobacillaceae | Lactobacillus | sp UMNPBX3 | 4.80 ± 1.36 | 0.015 | 2.25E-2 (1.01E-2 - 8.61E-2) | | 8.95E-4 (4.42E-5 - 2.45E-3) | |
| Firmicutes | Bacilli | Lactobacillales | Enterococcaceae | Enterococcus | sp HMSC065H03 | 4.74 ± 1.61 | 0.044 | 7.69E-4 (5.07E-4 - 1.73E-3) | | 0.00E+0 (0.00E+0 - 1.67E-5) | |
| Firmicutes | Bacilli | Lactobacillales | Enterococcaceae | Enterococcus | sp HMSC035C10 | 4.65 ± 1.33 | 0.015 | 1.80E-2 (1.24E-2 - 3.31E-2) | | 2.87E-4 (4.49E-5 - 7.76E-4) | |
| Firmicutes | Bacilli | Lactobacillales | Lactobacillaceae | Lactobacillus | intestinalis | 4.62 ± 1.43 | 0.022 | 3.40E-3 (2.16E-3 - 1.31E-2) | | 6.80E-5 (0.00E+0 - 4.34E-4) | |
| Firmicutes | Bacilli | Lactobacillales | Enterococcaceae | Enterococcus | sp HMSC063D12 | 4.61 ± 1.27 | 0.015 | 2.70E-1 (1.89E-1 - 5.01E-1) | | 2.73E-3 (6.24E-4 - 6.12E-3) | |
| Firmicutes | Bacilli | Lactobacillales | Enterococcaceae | Enterococcus | sp HMSC061C05 | 4.60 ± 1.33 | 0.015 | 9.45E-2 (6.58E-2 - 1.85E-1) | | 8.18E-4 (7.37E-5 - 2.17E-3) | |
| Firmicutes | Bacilli | Lactobacillales | Lactobacillaceae | Lactobacillus | ND | 4.59 ± 1.42 | 0.022 | 9.79E-1 (2.65E-1 - 2.51E+0) | | 2.07E-2 (8.53E-4 - 1.21E-1) | |
| Firmicutes | Bacilli | Lactobacillales | Enterococcaceae | Enterococcus | sp HMSC063C12 | 4.56 ± 1.47 | 0.029 | 3.03E-4 (1.56E-4 - 7.64E-4) | | 0.00E+0 (0.00E+0 - 1.53E-5) | |
| Firmicutes | Bacilli | Lactobacillales | Enterococcaceae | Enterococcus | villorum | 4.53 ± 1.41 | 0.022 | 1.24E-3 (7.08E-4 - 2.55E-3) | | 0.00E+0 (0.00E+0 - 1.44E-4) | |
| Firmicutes | Bacilli | Lactobacillales | Enterococcaceae | Enterococcus | sp HMSC076E04 | 4.48 ± 1.27 | 0.015 | 1.44E-1 (1.00E-1 - 2.80E-1) | | 1.72E-3 (5.00E-4 - 3.97E-3) | |
| Firmicutes | Bacilli | Lactobacillales | Enterococcaceae | Enterococcus | sp HMSC063H10 | 4.31 ± 1.30 | 0.018 | 1.18E-2 (7.90E-3 - 2.17E-2) | | 2.93E-4 (6.57E-5 - 5.57E-4) | |
| **Firmicutes** | **Bacilli** | **Lactobacillales** | **Enterococcaceae** | **Enterococcus** | **faecium** | 4.19 ± 1.25 | 0.018 | 1.09E-1 (7.54E-2 - 2.17E-1) | | 1.54E-3 (4.86E-4 - 7.48E-3) | |
| Firmicutes | Bacilli | Lactobacillales | Enterococcaceae | Enterococcus | durans | 4.08 ± 1.34 | 0.035 | 2.67E-2 (9.72E-3 - 9.28E-2) | | 6.61E-4 (1.07E-4 - 3.42E-3) | |
| Firmicutes | Bacilli | Lactobacillales | Enterococcaceae | Enterococcus | sp HMSC067C01 | 3.98 ± 1.36 | 0.047 | 1.06E-1 (7.23E-2 - 2.01E-1) | | 1.83E-3 (2.59E-4 - 5.13E-3) | |
| **Lower in HR at week 4** | | | | | | | | | | | |
| Bacteroidetes | Bacteroidia | Bacteroidales | Bacteroidaceae | ND | ND | -6.79 ± 1.75 | 0.010 | 2.22E-5 (0.00E+0 - 2.30E-4) | | 4.44E-3 (4.05E-4 - 3.44E-2) | |
| Bacteroidetes | Bacteroidia | Bacteroidales | Tannerellaceae | Tannerella | sp 6 1 58FAA CT1 | -6.76 ± 2.05 | 0.019 | 0.00E+0 (0.00E+0 - 0.00E+0) | | 8.15E-4 (0.00E+0 - 2.03E-3) | |
| Firmicutes | Bacilli | Lactobacillales | Streptococcaceae | Streptococcus | gallolyticus | -6.70 ± 1.71 | 0.010 | 1.45E-4 (4.03E-5 - 4.15E-4) | | 2.57E-2 (6.67E-4 - 9.61E-2) | |
| Bacteroidetes | Bacteroidia | Bacteroidales | Bacteroidaceae | Bacteroides | clarus | -6.58 ± 1.93 | 0.016 | 0.00E+0 (0.00E+0 - 1.46E-4) | | 1.47E-4 (5.66E-5 - 1.81E-2) | |
| Proteobacteria | Epsilonproteobacteria | Campylobacterales | Helicobacteraceae | Helicobacter | canis | -6.57 ± 2.22 | 0.042 | 0.00E+0 (0.00E+0 - 1.89E-5) | | 3.07E-5 (0.00E+0 - 9.63E-4) | |
| Firmicutes | Bacilli | Lactobacillales | Streptococcaceae | Lactococcus | lactis | -6.33 ± 1.41 | 0.003 | 2.17E-3 (6.63E-4 - 2.78E-3) | | 2.63E-2 (1.76E-3 - 2.53E-1) | |
| Bacteroidetes | Bacteroidia | Bacteroidales | Bacteroidaceae | Bacteroides | stercoris | -5.81 ± 1.35 | 0.004 | 3.07E-3 (1.43E-3 - 1.29E-2) | | 3.35E-2 (9.80E-3 - 1.31E+0) | |
| Actinobacteria | Actinobacteria | Bifidobacteriales | Bifidobacteriaceae | Bifidobacterium | pseudolongum | -5.77 ± 1.66 | 0.015 | 9.30E-5 (4.03E-5 - 2.16E-4) | | 3.25E-3 (4.60E-5 - 1.30E-2) | |
| Firmicutes | Bacilli | Lactobacillales | Streptococcaceae | Streptococcus | infantarius | -5.64 ± 1.56 | 0.015 | 9.76E-4 (6.98E-4 - 2.10E-3) | | 2.72E-1 (3.15E-3 - 9.33E-1) | |
| Firmicutes | Bacilli | Lactobacillales | Streptococcaceae | Streptococcus | ND | -5.52 ± 1.56 | 0.015 | 2.22E-2 (9.35E-3 - 2.94E-2) | | 6.48E+0 (7.45E-2 - 2.31E+1) | |
| Firmicutes | Bacilli | Lactobacillales | Streptococcaceae | Streptococcus | lutetiensis | -5.47 ± 1.59 | 0.015 | 3.92E-3 (1.19E-3 - 4.75E-3) | | 1.07E+0 (1.22E-2 - 4.30E+0) | |
| Firmicutes | Bacilli | Lactobacillales | Streptococcaceae | Streptococcus | equinus | -5.36 ± 1.58 | 0.016 | 8.83E-4 (7.10E-4 - 1.28E-3) | | 1.41E-1 (1.68E-3 - 5.44E-1) | |
| Bacteroidetes | Bacteroidia | Bacteroidales | Bacteroidaceae | Bacteroides | coprocola | -4.81 ± 1.55 | 0.029 | 3.45E-3 (1.38E-4 - 9.44E-3) | | 5.93E-2 (9.39E-3 - 2.72E-1) | |
| Firmicutes | Bacilli | Lactobacillales | Streptococcaceae | Streptococcus | thermophilus | -4.58 ± 1.37 | 0.018 | 1.31E-4 (1.03E-4 - 1.02E-3) | | 3.35E-3 (6.21E-4 - 5.68E-2) | |

* Log_2_FC = 1 represents 2 fold-change of HR:LR

** P values were adjusted using the false discovery rate

FC: fold change (HR/LR), ND: no data (unknown)
