# Supplementary material for: The microbiota of healthy dogs demonstrates individualized responses to synbiotic supplementation in a randomized controlled trial
Source: Anim Microbiome. 2021 May 10;3:36. doi: 10.1186/s42523-021-00098-0 (PMC8111948; doi:10.1186/s42523-021-00098-0)
Supplement: Supplementary file 10 — Additional file 10: Table S9A. Bacterial species that were significantly different in the differential abundance analysis (|fold change| ≥ 2 and adjusted p < 0.05) at week 4 relative to week 0 in high-responders (n = 8). Species in bold were present in the synbiotic supplement. Table S9B. Bacterial species that were significantly different in the differential abundance analysis (|fold change| ≥ 2 and adjusted p < 0.05) at week 4 relative to week 0 in low-responders (n = 8). [file 42523_2021_98_MOESM10_ESM.docx]

**Supplemental Table 9A.** Bacterial species that were significantly different in the differential abundance analysis (|fold change| ≥ 2 and adjusted p < 0.05) at week 4 relative to week 0 in high-responders (n=8). Species in bold were present in the synbiotic supplement.

| **Phylum** | **Class** | **Order** | **Family** | **Genus** | **Species** | **DESeq2 results**  **week 4/week 0** | | **Relative abundance, in %** | | | |
| --- | --- | --- | --- | --- | --- | --- | --- | --- | --- | --- | --- |
|  |  |  |  |  |  | **Log 2 FC***  **mean ± SE** | **Adjusted p**** | **Week 0**  **Median (IQR)** | | **Week 4**  **Median (IQR)** | |
| **Increased at week 4** | | | | | | | | | | | |
| **Firmicutes** | **Bacilli** | **Lactobacillales** | **Lactobacillaceae** | **Lactobacillus** | **reuteri** | **12.92±1.07** | **1.12E-30** | 1.20E-3 (4.96E-4 - 2.01E-3) | | 5.00E+0 (2.32E+0 - 1.93E+1) | |
| Firmicutes | Bacilli | Lactobacillales | Enterococcaceae | Enterococcus | sp HMSC061C05 | 12.42±1.40 | 1.81E-16 | 0.00E+0 (0.00E+0 - 5.05E-5) | | 9.45E-2 (6.58E-2 - 1.85E-1) | |
| Firmicutes | Bacilli | Lactobacillales | Enterococcaceae | Enterococcus | sp HMSC076E04 | 11.35±1.43 | 2.25E-13 | 0.00E+0 (0.00E+0 - 8.25E-5) | | 1.44E-1 (1.00E-1 - 2.80E-1) | |
| Firmicutes | Bacilli | Lactobacillales | Enterococcaceae | Enterococcus | sp HMSC063D12 | 10.87±1.29 | 4.97E-15 | 9.87E-5 (0.00E+0 - 1.94E-4) | | 2.70E-1 (1.89E-1 - 5.01E-1) | |
| Firmicutes | Bacilli | Lactobacillales | Lactobacillaceae | Lactobacillus | sp UMNPBX3 | 10.71±1.46 | 2.30E-11 | 0.00E+0 (0.00E+0 - 2.60E-5) | | 2.25E-2 (1.01E-2 - 8.61E-2) | |
| Firmicutes | Bacilli | Lactobacillales | Enterococcaceae | Enterococcus | sp HMSC035C10 | 10.68±1.40 | 2.60E-12 | 0.00E+0 (0.00E+0 - 1.12E-5) | | 1.80E-2 (1.24E-2 - 3.31E-2) | |
| Firmicutes | Bacilli | Lactobacillales | Lactobacillaceae | Lactobacillus | ND | 10.40±1.12 | 3.74E-18 | 7.95E-4 (2.04E-4 - 1.88E-3) | | 9.79E-1 (2.65E-1 - 2.51E+0) | |
| Firmicutes | Bacilli | Lactobacillales | Enterococcaceae | Enterococcus | sp HMSC067C01 | 10.10±1.44 | 1.64E-10 | 0.00E+0 (0.00E+0 - 1.45E-4) | | 1.06E-1 (7.23E-2 - 2.01E-1) | |
| Firmicutes | Bacilli | Lactobacillales | Enterococcaceae | Enterococcus | sp HMSC34G12 | 10.02±1.57 | 9.57E-09 | 0.00E+0 (0.00E+0 - 0.00E+0) | | 5.80E-3 (4.24E-3 - 1.33E-2) | |
| **Firmicutes** | **Bacilli** | **Lactobacillales** | **Lactobacillaceae** | **Lactobacillus** | **rhamnosus** | **9.89±2.10** | **5.78E-05** | 0.00E+0 (0.00E+0 - 0.00E+0) | | 8.79E-4 (6.24E-5 - 1.14E-2) | |
| **Firmicutes** | **Bacilli** | **Lactobacillales** | **Lactobacillaceae** | **Pediococcus** | **acidilactici** | **9.77±1.38** | **1.05E-10** | 3.27E-5 (0.00E+0 - 6.85E-5) | | 2.32E-2 (1.42E-2 - 4.19E-2) | |
| Firmicutes | Bacilli | Lactobacillales | Lactobacillaceae | Lactobacillus | intestinalis | 9.47±1.55 | 4.09E-08 | 0.00E+0 (0.00E+0 - 0.00E+0) | | 3.40E-3 (2.16E-3 - 1.31E-2) | |
| Firmicutes | Bacilli | Lactobacillales | Enterococcaceae | Enterococcus | sp 3G1 DIV0629 | 8.92±1.37 | 4.22E-09 | 0.00E+0 (0.00E+0 - 8.25E-5) | | 1.22E-2 (8.60E-3 - 2.38E-2) | |
| Firmicutes | Bacilli | Lactobacillales | Enterococcaceae | Enterococcus | sp HMSC069A01 | 8.74±1.37 | 9.18E-09 | 0.00E+0 (0.00E+0 - 1.83E-5) | | 6.32E-3 (4.35E-3 - 1.20E-2) | |
| Firmicutes | Bacilli | Lactobacillales | Enterococcaceae | Enterococcus | sp 10A9 DIV0425 | 8.63±1.47 | 1.89E-07 | 0.00E+0 (0.00E+0 - 0.00E+0) | | 2.45E-3 (1.93E-3 - 9.49E-3) | |
| Firmicutes | Bacilli | Lactobacillales | Lactobacillaceae | Lactobacillus | plantarum | 8.62±1.26 | 4.80E-10 | 8.87E-5 (6.19E-5 - 3.45E-4) | | 5.73E-2 (9.33E-3 - 2.04E-1) | |
| Firmicutes | Bacilli | Lactobacillales | Enterococcaceae | Enterococcus | sp HMSC063H10 | 8.61±1.24 | 2.90E-10 | 5.50E-5 (0.00E+0 - 8.21E-5) | | 1.18E-2 (7.90E-3 - 2.17E-2) | |
| Firmicutes | Bacilli | Lactobacillales | Lactobacillaceae | Lactobacillus | frumenti | 8.44±1.72 | 2.38E-05 | 0.00E+0 (0.00E+0 - 0.00E+0) | | 1.31E-3 (5.69E-4 - 6.12E-3) | |
| Firmicutes | Bacilli | Lactobacillales | Lactobacillaceae | Lactobacillus | hominis | 8.42±1.58 | 2.90E-06 | 0.00E+0 (0.00E+0 - 0.00E+0) | | 1.40E-3 (8.19E-4 - 6.55E-3) | |
| Firmicutes | Bacilli | Lactobacillales | Enterococcaceae | Enterococcus | sp HMSC072D11 | 8.32±1.50 | 1.06E-06 | 0.00E+0 (0.00E+0 - 0.00E+0) | | 2.41E-3 (1.05E-3 - 4.07E-3) | |
| Firmicutes | Bacilli | Lactobacillales | Lactobacillaceae | Lactobacillus | vaginalis | 8.14±1.59 | 8.24E-06 | 0.00E+0 (0.00E+0 - 0.00E+0) | | 1.02E-3 (7.90E-4 - 4.19E-3) | |
| Firmicutes | Bacilli | Lactobacillales | Enterococcaceae | Enterococcus | sp HMSC072F02 | 7.97±1.61 | 1.80E-05 | 0.00E+0 (0.00E+0 - 6.71E-5) | | 5.02E-2 (3.22E-2 - 9.21E-2) | |
| Firmicutes | Bacilli | Lactobacillales | Lactobacillaceae | Lactobacillus | sp ASF360 | 7.88±1.56 | 1.22E-05 | 0.00E+0 (0.00E+0 - 0.00E+0) | | 1.29E-3 (6.15E-4 - 4.04E-3) | |
| **Actinobacteria** | **Actinobacteria** | **Bifidobacteriales** | **Bifidobacteriaceae** | **Bifidobacterium** | **animalis** | **7.78±1.35** | **2.71E-07** | 2.29E-4 (0.00E+0 - 5.87E-4) | | 1.11E-1 (8.04E-2 - 5.06E-1) | |
| **Firmicutes** | **Bacilli** | **Lactobacillales** | **Lactobacillaceae** | **Lactobacillus** | **fermentum** | **7.76±1.33** | **1.89E-07** | 3.66E-5 (0.00E+0 - 1.47E-4) | | 1.84E-2 (6.08E-3 - 3.14E-2) | |
| Firmicutes | Bacilli | Lactobacillales | Enterococcaceae | Enterococcus | durans | 7.55±1.40 | 2.29E-06 | 5.50E-5 (0.00E+0 - 2.12E-4) | | 2.67E-2 (9.72E-3 - 9.28E-2) | |
| Firmicutes | Bacilli | Lactobacillales | Lactobacillaceae | Lactobacillus | sp HMSC24D01 | 7.45±1.60 | 7.09E-05 | 0.00E+0 (0.00E+0 - 0.00E+0) | | 8.58E-4 (2.83E-4 - 2.27E-3) | |
| Firmicutes | Bacilli | Lactobacillales | Aerococcaceae | Facklamia | ND | 7.44±1.58 | 5.78E-05 | 0.00E+0 (0.00E+0 - 0.00E+0) | | 8.69E-4 (5.71E-4 - 2.12E-3) | |
| **Firmicutes** | **Bacilli** | **Lactobacillales** | **Enterococcaceae** | **Enterococcus** | **faecium** | **7.23±1.36** | **2.90E-06** | 1.30E-4 (5.60E-5 - 5.97E-4) | | 1.09E-1 (7.54E-2 - 2.17E-1) | |
| **Firmicutes** | **Bacilli** | **Lactobacillales** | **Lactobacillaceae** | **Lactobacillus** | **acidophilus** | **7.15±1.23** | **2.35E-07** | 2.39E-3 (1.01E-3 - 1.33E-2) | | 1.04E+0 (2.63E-1 - 2.74E+0) | |
| Firmicutes | Bacilli | Lactobacillales | Enterococcaceae | Enterococcus | villorum | 7.09±1.45 | 2.38E-05 | 0.00E+0 (0.00E+0 - 0.00E+0) | | 1.24E-3 (7.08E-4 - 2.55E-3) | |
| Firmicutes | Bacilli | Lactobacillales | Lactobacillaceae | ND | ND | 7.03±1.60 | 2.30E-04 | 0.00E+0 (0.00E+0 - 8.25E-5) | | 1.47E-3 (4.59E-4 - 8.10E-3) | |
| Firmicutes | Bacilli | Lactobacillales | Enterococcaceae | Enterococcus | sp HMSC065H03 | 6.99±1.65 | 4.46E-04 | 0.00E+0 (0.00E+0 - 0.00E+0) | | 7.69E-4 (5.07E-4 - 1.73E-3) | |
| Firmicutes | Bacilli | Lactobacillales | Lactobacillaceae | Lactobacillus | salivarius | 6.85±1.38 | 1.80E-05 | 0.00E+0 (0.00E+0 - 4.93E-5) | | 1.46E-3 (8.12E-4 - 4.53E-3) | |
| Firmicutes | Bacilli | Lactobacillales | Enterococcaceae | Enterococcus | sp 4E1 DIV0656 | 6.59±1.98 | 1.28E-02 | 0.00E+0 (0.00E+0 - 8.21E-5) | | 2.82E-4 (0.00E+0 - 3.59E-3) | |
| Firmicutes | Bacilli | Lactobacillales | Lactobacillaceae | Lactobacillus | ingluviei | 6.57±1.72 | 2.59E-03 | 0.00E+0 (0.00E+0 - 0.00E+0) | | 3.12E-4 (2.20E-4 - 1.68E-3) | |
| Firmicutes | Bacilli | Lactobacillales | Leuconostocaceae | Weissella | cibaria | 6.27±1.91 | 1.39E-02 | 0.00E+0 (0.00E+0 - 1.63E-5) | | 1.43E-4 (6.24E-5 - 2.84E-3) | |
| Firmicutes | Bacilli | Lactobacillales | Enterococcaceae | Enterococcus | pseudoavium | 6.03±1.61 | 3.27E-03 | 0.00E+0 (0.00E+0 - 0.00E+0) | | 5.71E-4 (3.50E-4 - 7.45E-4) | |
| Firmicutes | Bacilli | Lactobacillales | Lactobacillaceae | Lactobacillus | taiwanensis | 5.96±1.57 | 2.66E-03 | 0.00E+0 (0.00E+0 - 1.12E-5) | | 2.86E-4 (1.84E-4 - 1.80E-3) | |
| Firmicutes | Bacilli | Lactobacillales | Enterococcaceae | Enterococcus | sp HMSC034B11 | 5.96±1.64 | 5.00E-03 | 0.00E+0 (0.00E+0 - 1.12E-5) | | 4.47E-4 (2.50E-4 - 1.51E-3) | |
| Proteobacteria | Gammaproteobacteria | Pseudomonadales | Moraxellaceae | Acinetobacter | calcoaceticus | 5.95±1.65 | 5.33E-03 | 0.00E+0 (0.00E+0 - 0.00E+0) | | 3.77E-4 (2.85E-4 - 7.01E-4) | |
| Firmicutes | Bacilli | Lactobacillales | Leuconostocaceae | Leuconostoc | ND | 5.90±1.64 | 5.33E-03 | 0.00E+0 (0.00E+0 - 8.81E-5) | | 4.92E-4 (7.50E-5 - 1.08E-2) | |
| Firmicutes | Bacilli | Lactobacillales | Enterococcaceae | Enterococcus | sp HMSC077E07 | 5.86±1.63 | 5.33E-03 | 0.00E+0 (0.00E+0 - 0.00E+0) | | 4.38E-4 (2.15E-4 - 9.93E-4) | |
| Firmicutes | Bacilli | Lactobacillales | Enterococcaceae | Enterococcus | sp HMSC063C12 | 5.64±1.53 | 4.20E-03 | 0.00E+0 (0.00E+0 - 0.00E+0) | | 3.03E-4 (1.56E-4 - 7.64E-4) | |
| Firmicutes | Bacilli | Lactobacillales | Enterococcaceae | Enterococcus | sp HMSC035B04 | 5.52±1.66 | 1.28E-02 | 0.00E+0 (0.00E+0 - 0.00E+0) | | 2.95E-4 (1.48E-4 - 5.05E-4) | |
| Firmicutes | Bacilli | Lactobacillales | Lactobacillaceae | Lactobacillus | mucosae | 5.40±1.82 | 3.57E-02 | 0.00E+0 (0.00E+0 - 0.00E+0) | | 1.58E-4 (3.32E-5 - 5.01E-4) | |
| Firmicutes | Bacilli | Lactobacillales | Lactobacillaceae | Lactobacillus | murinus | 5.38±1.61 | 1.25E-02 | 0.00E+0 (0.00E+0 - 0.00E+0) | | 3.64E-4 (1.21E-4 - 1.05E-3) | |
| Firmicutes | Bacilli | Lactobacillales | Enterococcaceae | Enterococcus | sp 10F3 DIV0382 | 5.18±1.50 | 8.74E-03 | 8.37E-5 (0.00E+0 - 4.92E-4) | | 3.74E-2 (2.43E-2 - 7.36E-2) | |
| Firmicutes | Bacilli | Lactobacillales | Leuconostocaceae | Leuconostoc | citreum | 5.11±1.63 | 2.17E-02 | 6.89E-5 (0.00E+0 - 3.63E-4) | | 2.79E-3 (2.01E-4 - 8.29E-3) | |
| Firmicutes | Bacilli | Lactobacillales | Enterococcaceae | Enterococcus | sp HMSC060D09 | 5.03±1.70 | 3.57E-02 | 0.00E+0 (0.00E+0 - 0.00E+0) | | 1.68E-4 (8.78E-5 - 4.23E-4) | |
| Firmicutes | Bacilli | Lactobacillales | Enterococcaceae | Enterococcus | malodoratus | 4.32±1.32 | 1.39E-02 | 0.00E+0 (0.00E+0 - 6.56E-5) | | 4.33E-4 (3.66E-4 - 1.02E-3) | |
| Firmicutes | Bacilli | Lactobacillales | Lactobacillaceae | Lactobacillus | johnsonii | 4.25±1.20 | 6.21E-03 | 5.39E-5 (0.00E+0 - 6.85E-5) | | 4.22E-4 (3.09E-4 - 1.48E-3) | |
| Firmicutes | Bacilli | Lactobacillales | Streptococcaceae | Lactococcus | ND | 3.86±1.35 | 4.95E-02 | 3.27E-5 (0.00E+0 - 1.56E-4) | | 4.10E-4 (1.32E-4 - 1.38E-3) | |
| **Decreased at week 4** | | | | | | | | | | | |
| Actinobacteria | Actinobacteria | Bifidobacteriales | Bifidobacteriaceae | Bifidobacterium | pseudolongum | -6.53±1.95 | 1.21E-02 | 1.25E-4 (0.00E+0 - 5.66E-2) | | 9.30E-5 (4.03E-5 - 2.16E-4) | |
| Bacteroidetes | Bacteroidia | Bacteroidales | Prevotellaceae | Prevotella | copri | -6.18±1.85 | 1.21E-02 | 1.48E-3 (4.86E-4 - 5.35E-1) | | 8.02E-4 (4.19E-4 - 6.36E-3) | |
| Bacteroidetes | Bacteroidia | Bacteroidales | Bacteroidaceae | Bacteroides | plebeius | -4.77±1.51 | 2.03E-02 | 4.28E-4 (1.75E-4 - 2.48E-1) | | 6.49E-3 (2.69E-3 - 7.40E-3) | |
| Bacteroidetes | Bacteroidia | Bacteroidales | Bacteroidaceae | Bacteroides | fragilis | -4.21±1.41 | 3.49E-02 | 2.52E-3 (5.11E-4 - 9.61E-2) | | 1.39E-3 (8.71E-4 - 4.20E-3) | |
| Proteobacteria | Gammaproteobacteria | Enterobacterales | Enterobacteriaceae | Shigella | ND | -3.97±1.27 | 2.29E-02 | 1.04E-1 (2.81E-2 - 7.28E-1) | | 2.90E-2 (2.11E-2 - 6.75E-2) | |
| Bacteroidetes | Bacteroidia | Bacteroidales | Bacteroidaceae | Bacteroides | stercoris | -3.92±1.31 | 3.39E-02 | 6.74E-3 (1.18E-3 - 2.30E-1) | | 3.07E-3 (1.43E-3 - 1.29E-2) | |
| Proteobacteria | Gammaproteobacteria | Enterobacterales | Enterobacteriaceae | Shigella | sonnei | -3.76±1.30 | 4.47E-02 | 3.21E-2 (8.86E-3 - 2.35E-1) | | 1.73E-2 (8.28E-3 - 2.87E-2) | |

* Log_2_FC > 1 represents 2 fold-change at week 4 or 6 compared to week 0

** P values were adjusted using the false discovery rate

FC: fold change, ND: no data (unknown), NS: not significant

**Supplemental Table 9B.** Bacterial species that were significantly different in the differential abundance analysis (|fold change| ≥ 2 and adjusted p < 0.05) at week 4 relative to week 0 in low-responders (n=8).

| **Phylum** | **Class** | **Order** | **Family** | **Genus** | **Species** | **DESeq2 results**  **week 4/week 0** | | **Relative abundance, in %** | | | |
| --- | --- | --- | --- | --- | --- | --- | --- | --- | --- | --- | --- |
|  |  |  |  |  |  | **Log 2 FC***  **mean ± SE** | **Adjusted p**** | **Week 0**  **Median (IQR)** | | **Week 4**  **Median (IQR)** | |
| Firmicutes | Bacilli | Lactobacillales | Lactobacillaceae | Lactobacillus | sakei | -9.82±1.69 | 1.44E-05 | 1.57E-2 (2.26E-3 - 3.37E-1) | | 8.30E-4 (3.41E-4 - 4.81E-3) | |

* Log_2_FC > 1 represents 2 fold-change at week 4 or 6 compared to week 0

** P values were adjusted using the false discovery rate

FC: fold change, ND: no data (unknown), NS: not significant
