# Supplementary material for: The microbiota of healthy dogs demonstrates individualized responses to synbiotic supplementation in a randomized controlled trial
Source: Anim Microbiome. 2021 May 10;3:36. doi: 10.1186/s42523-021-00098-0 (PMC8111948; doi:10.1186/s42523-021-00098-0)
Supplement: Supplementary file 11 — Additional file 11: Table S10A. Gut bacteria from samples collected at baseline that were significantly different in the differential abundance analysis (|fold change| ≥ 2 and p < 0.05) between high-responders (HR, n = 8) and mid-responders (MR, n = 7). Species in red were identified with the same trend when HR was compared with low-responders (LR). Table S10B. Gut bacteria from samples collected at baseline that were significantly different in the differential abundance analysis (|fold change| ≥ 2 and p < 0.05) between mid-responders (MR, n = 7) and low-responders (LR, n = 8). Species in red were identified with the same trend when high-responders (HR) was compared with LR. [file 42523_2021_98_MOESM11_ESM.docx]

# **Supplemental Table 10A.** Gut bacteria from samples collected at baseline that were significantly different in the differential abundance analysis (|fold change| ≥ 2 and p < 0.05) between high-responders (HR, n = 8) and mid-responders (MR, n = 7). Species in red were identified with the same trend when HR was compared with low-responders (LR).

| **Phylum** | **Class** | **Order** | **Family** | **Genus** | **Species** | **HR vs MR** | | **Relative abundance, in %** | | | |
| --- | --- | --- | --- | --- | --- | --- | --- | --- | --- | --- | --- |
|  |  |  |  |  |  | **Log 2 FC***  **mean ± SE** | **Adjusted p**** | **HR Median (IQR)** | | **MR Median (IQR)** | |
| **Higher in HR** | | | | | | | | | | | |
| **Bacteroidetes** | **Bacteroidia** | **Bacteroidales** | **Prevotellaceae** | **Prevotella** | **copri** | 6.07 ± 1.89 | 0.040 | 1.48E-3 (4.86E-4 - 5.35E-1) | | 1.22E-3 (3.02E-4 - 1.65E-2) | |
| **Lower in HR** | | | | | | | | | | | |
| Proteobacteria | Gammaproteobacteria | Enterobacterales | Enterobacteriaceae | Citrobacter | sp KTE151 | -24.18 ± 2.58 | < 0.001 | 0.00E+0 (0.00E+0 - 0.00E+0) | | 3.65E-4 (0.00E+0 - 8.10E-3) | |
| Proteobacteria | Gammaproteobacteria | Enterobacterales | Enterobacteriaceae | Citrobacter | sp L55 | -9.97 ± 2.29 | 0.003 | 0.00E+0 (0.00E+0 - 0.00E+0) | | 1.77E-4 (9.44E-5 - 1.47E-2) | |
| Actinobacteria | Coriobacteriia | Eggerthellales | Eggerthellaceae | Slackia | piriformis | -8.87 ± 2.15 | 0.003 | 6.89E-5 (0.00E+0 - 2.83E-4) | | 6.26E-5 (0.00E+0 - 2.55E-2) | |
| Proteobacteria | Gammaproteobacteria | Enterobacterales | Hafniaceae | Hafnia | ND | -8.56 ± 2.60 | 0.036 | 0.00E+0 (0.00E+0 - 1.12E-5) | | 0.00E+0 (0.00E+0 - 7.22E-3) | |
| Proteobacteria | Betaproteobacteria | Burkholderiales | ND | ND | ND | -8.37 ± 2.61 | 0.040 | 0.00E+0 (0.00E+0 - 6.57E-5) | | 0.00E+0 (0.00E+0 - 1.44E-2) | |
| Firmicutes | Erysipelotrichia | Erysipelotrichales | Erysipelotrichaceae | Erysipelatoclostridium | spiroforme | -7.90 ± 1.90 | 0.003 | 3.35E-5 (0.00E+0 - 9.98E-5) | | 7.16E-4 (7.90E-5 - 1.22E-2) | |
| **Firmicutes** | **Erysipelotrichia** | **Erysipelotrichales** | **Erysipelotrichaceae** | **Allobaculum** | **stercoricanis** | -7.31 ± 1.76 | 0.003 | 3.70E-4 (2.04E-4 - 2.64E-3) | | 1.67E-3 (1.51E-4 - 1.74E-1) | |
| Proteobacteria | Gammaproteobacteria | Enterobacterales | Enterobacteriaceae | Citrobacter | ND | -6.97 ± 1.64 | 0.003 | 2.66E-3 (4.58E-4 - 5.41E-3) | | 3.14E-2 (7.96E-3 - 9.65E-1) | |
| Firmicutes | Clostridia | Clostridiales | Peptostreptococcaceae | Terrisporobacter | glycolicus | -6.59 ± 1.74 | 0.010 | 3.35E-5 (0.00E+0 - 1.96E-4) | | 4.50E-3 (1.66E-3 - 1.52E-2) | |
| **Firmicutes** | **Bacilli** | **Lactobacillales** | **Streptococcaceae** | **Lactococcus** | **lactis** | -6.39 ± 1.78 | 0.019 | 3.65E-4 (1.00E-4 - 2.34E-3) | | 4.51E-3 (1.75E-3 - 4.45E-2) | |
| Firmicutes | Clostridia | Clostridiales | Peptostreptococcaceae | Romboutsia | lituseburensis | -6.20 ± 1.83 | 0.031 | 2.24E-5 (0.00E+0 - 8.21E-5) | | 5.01E-4 (6.26E-5 - 5.32E-3) | |
| **Firmicutes** | **Bacilli** | **Lactobacillales** | **Leuconostocaceae** | **Leuconostoc** | **gelidum** | -6.02 ± 1.35 | 0.002 | 6.82E-4 (2.23E-4 - 1.52E-3) | | 2.47E-2 (1.42E-3 - 9.67E-2) | |
| Firmicutes | Clostridia | Clostridiales | Peptostreptococcaceae | Romboutsia | dakarense | -5.88 ± 1.75 | 0.031 | 2.24E-5 (0.00E+0 - 6.77E-5) | | 5.01E-4 (2.83E-4 - 7.42E-3) | |
| Proteobacteria | Gammaproteobacteria | Enterobacterales | Enterobacteriaceae | Enterobacter | hormaechei | -5.59 ± 1.66 | 0.031 | 0.00E+0 (0.00E+0 - 1.88E-4) | | 2.19E-3 (1.63E-3 - 5.98E-3) | |
| Firmicutes | Clostridia | Clostridiales | Peptostreptococcaceae | Romboutsia | timonensis | -5.38 ± 1.67 | 0.040 | 1.17E-4 (0.00E+0 - 4.28E-4) | | 1.11E-2 (1.87E-4 - 1.85E-2) | |
| Firmicutes | Bacilli | Lactobacillales | Lactobacillaceae | Lactobacillus | reuteri | -5.05 ± 1.36 | 0.012 | 1.20E-3 (4.96E-4 - 2.01E-3) | | 3.63E-2 (4.34E-3 - 7.39E-2) | |
| **Firmicutes** | **Erysipelotrichia** | **Erysipelotrichales** | **Erysipelotrichaceae** | **Turicibacter** | **ND** | -4.95 ± 1.40 | 0.019 | 8.21E-4 (4.58E-4 - 1.19E-3) | | 8.98E-3 (2.96E-3 - 4.29E-2) | |

* Log_2_FC = 1 represents 2 fold-change of HR:MR

** P values were adjusted using the false discovery rate

FC: fold change (HR/LR), ND: no data (unknown)

# **Supplemental Table 10B.** Gut bacteria from samples collected at baseline that were significantly different in the differential abundance analysis (|fold change| ≥ 2 and p < 0.05) between mid-responders (MR, n = 7) and low-responders (LR, n = 8). Species in red were identified with the same trend when high-responders (HR) was compared with LR.

| **Phylum** | **Class** | **Order** | **Family** | **Genus** | **Species** | **MR vs LR** | | **Relative abundance, in %** | | | |
| --- | --- | --- | --- | --- | --- | --- | --- | --- | --- | --- | --- |
|  |  |  |  |  |  | **Log 2 FC***  **mean ± SE** | **Adjusted p**** | **MR**  **Median (IQR)** | | **LR**  **Median (IQR)** | |
| **Higher in MR** | | | | | | | | | | | |
| Proteobacteria | Betaproteobacteria | Burkholderiales | ND | ND | ND | 9.03 ± 2.74 | 0.028 | 0.00E+0 (0.00E+0 - 1.44E-2) | | 0.00E+0 (0.00E+0 - 1.81E-5) | |
| Proteobacteria | Gammaproteobacteria | Enterobacterales | Morganellaceae | Providencia | alcalifaciens | 8.34 ± 2.60 | 0.030 | 0.00E+0 (0.00E+0 - 3.17E-3) | | 0.00E+0 (0.00E+0 - 0.00E+0) | |
| Actinobacteria | Coriobacteriia | Eggerthellales | Eggerthellaceae | Slackia | piriformis | 7.53 ± 2.32 | 0.029 | 6.26E-5 (0.00E+0 - 2.55E-2) | | 2.90E-5 (0.00E+0 - 1.46E-4) | |
| Firmicutes | Bacilli | Bacillales | Paenibacillaceae | Paenibacillus | sp VT-16-81 | 7.38 ± 2.25 | 0.028 | 5.62E-5 (2.28E-5 - 1.40E-3) | | 0.00E+0 (0.00E+0 - 0.00E+0) | |
| Bacteroidetes | ND | ND | ND | ND | ND | 7.38 ± 2.53 | 0.046 | 6.11E-5 (0.00E+0 - 1.50E-3) | | 8.34E-2 (1.76E-2 - 1.29E-1) | |
| Proteobacteria | Gammaproteobacteria | Enterobacterales | Morganellaceae | Providencia | rettgeri | 7.28 ± 1.84 | 0.005 | 6.15E-3 (3.07E-3 - 9.10E-3) | | 0.00E+0 (0.00E+0 - 1.36E-5) | |
| Firmicutes | Clostridia | Clostridiales | Lachnospiraceae | Catonella | morbi | 6.88 ± 2.15 | 0.030 | 0.00E+0 (0.00E+0 - 0.00E+0) | | 0.00E+0 (0.00E+0 - 0.00E+0) | |
| Proteobacteria | Gammaproteobacteria | Enterobacterales | Enterobacteriaceae | Klebsiella | sp 4 1 44FAA | 6.72 ± 1.98 | 0.021 | 1.25E-4 (3.82E-5 - 6.29E-3) | | 1.14E-4 (4.19E-5 - 5.36E-4) | |
| Firmicutes | Erysipelotrichia | Erysipelotrichales | Erysipelotrichaceae | Erysipelatoclostridium | spiroforme | 6.62 ± 1.82 | 0.011 | 7.16E-4 (7.90E-5 - 1.22E-2) | | 1.09E-4 (0.00E+0 - 3.55E-4) | |
| Proteobacteria | Gammaproteobacteria | Enterobacterales | Enterobacteriaceae | Kluyvera | cryocrescens | 6.57 ± 1.79 | 0.011 | 1.41E-3 (1.19E-3 - 1.68E-3) | | 0.00E+0 (0.00E+0 - 5.66E-5) | |
| **Proteobacteria** | **Gammaproteobacteria** | **Enterobacterales** | **Enterobacteriaceae** | **Escherichia** | **ND** | 6.47 ± 1.30 | 0.000 | 1.38E+1 (6.12E+0 - 1.75E+1) | | 9.10E-2 (1.78E-2 - 2.90E-1) | |
| Firmicutes | Clostridia | Clostridiales | Clostridiaceae | Clostridium | baratii | 6.34 ± 2.01 | 0.035 | 9.12E-5 (3.13E-5 - 2.96E-2) | | 6.66E-5 (0.00E+0 - 2.87E-4) | |
| Firmicutes | Clostridia | Clostridiales | Ruminococcaceae | Fournierella | massiliensis | 6.33 ± 1.85 | 0.021 | 1.83E-4 (2.81E-5 - 7.05E-3) | | 1.40E-4 (3.72E-5 - 1.65E-4) | |
| Proteobacteria | Gammaproteobacteria | Enterobacterales | Enterobacteriaceae | Escherichia | sp KTE52 | 6.29 ± 1.61 | 0.005 | 4.33E-3 (1.79E-3 - 9.93E-3) | | 2.00E-4 (4.35E-5 - 7.08E-4) | |
| **Proteobacteria** | **Gammaproteobacteria** | **Enterobacterales** | **Enterobacteriaceae** | **Escherichia** | **sp KTE172** | 6.27 ± 1.37 | 0.001 | 3.15E-2 (1.33E-2 - 4.97E-2) | | 5.48E-4 (1.85E-4 - 3.27E-3) | |
| Proteobacteria | Gammaproteobacteria | Enterobacterales | Enterobacteriaceae | Citrobacter | rodentium | 6.25 ± 1.71 | 0.011 | 5.79E-3 (5.19E-4 - 2.47E-2) | | 5.28E-5 (0.00E+0 - 4.38E-4) | |
| Bacteroidetes | Bacteroidia | Bacteroidales | Bacteroidaceae | Bacteroides | sp 1 1 30 | 6.12 ± 1.89 | 0.029 | 4.28E-4 (6.77E-5 - 1.77E-3) | | 0.00E+0 (0.00E+0 - 1.37E-5) | |
| Bacteroidetes | Bacteroidia | Bacteroidales | Bacteroidaceae | Bacteroides | ovatus | 5.85 ± 1.50 | 0.005 | 0.00E+0 (0.00E+0 - 0.00E+0) | | 2.07E-3 (8.39E-4 - 2.85E-3) | |
| Firmicutes | Clostridia | Clostridiales | Lachnospiraceae | Anaerostipes | ND | 5.79 ± 1.97 | 0.045 | 4.78E-4 (3.05E-5 - 9.55E-3) | | 3.26E-4 (4.12E-5 - 4.88E-4) | |
| Firmicutes | Clostridia | Clostridiales | Clostridiaceae | Butyricicoccus | pullicaecorum | 5.79 ± 1.96 | 0.045 | 2.29E-4 (3.05E-5 - 1.28E-2) | | 2.79E-5 (0.00E+0 - 3.99E-4) | |
| Bacteroidetes | Bacteroidia | Bacteroidales | Bacteroidaceae | Bacteroides | xylanisolvens | 5.76 ± 1.69 | 0.021 | 1.30E-3 (2.90E-4 - 3.09E-3) | | 0.00E+0 (0.00E+0 - 1.10E-4) | |
| Proteobacteria | Gammaproteobacteria | Enterobacterales | Enterobacteriaceae | Escherichia | marmotae | 5.65 ± 1.46 | 0.006 | 3.79E-2 (6.18E-3 - 1.11E-1) | | 5.48E-4 (1.37E-4 - 1.63E-3) | |
| Proteobacteria | Gammaproteobacteria | Enterobacterales | Enterobacteriaceae | Citrobacter | freundii complex sp CFNIH2 | 5.56 ± 1.87 | 0.045 | 1.52E-3 (4.13E-4 - 1.25E-2) | | 9.93E-5 (0.00E+0 - 3.61E-4) | |
| Proteobacteria | Gammaproteobacteria | Enterobacterales | Enterobacteriaceae | Escherichia | fergusonii | 5.49 ± 1.32 | 0.004 | 1.34E-1 (3.39E-2 - 1.48E-1) | | 1.84E-3 (4.37E-4 - 1.42E-2) | |
| **Proteobacteria** | **Gammaproteobacteria** | **ND** | **ND** | **ND** | **ND** | 5.44 ± 1.22 | 0.002 | 1.79E-2 (5.97E-3 - 6.25E-2) | | 6.79E-4 (3.31E-4 - 2.20E-3) | |
| Proteobacteria | Gammaproteobacteria | Enterobacterales | Enterobacteriaceae | Franconibacter | helveticus | 5.40 ± 1.83 | 0.045 | 4.38E-4 (1.83E-4 - 1.84E-3) | | 0.00E+0 (0.00E+0 - 5.66E-5) | |
| **Proteobacteria** | **Gammaproteobacteria** | **Enterobacterales** | **Enterobacteriaceae** | **Escherichia** | **coli** | 5.40 ± 1.30 | 0.004 | 8.55E+0 (2.36E+0 - 1.09E+1) | | 6.98E-2 (1.78E-2 - 5.89E-1) | |
| **Proteobacteria** | **Gammaproteobacteria** | **Enterobacterales** | **Enterobacteriaceae** | **Escherichia** | **albertii** | 5.28 ± 1.31 | 0.005 | 3.12E-1 (1.53E-1 - 4.89E-1) | | 3.71E-3 (7.46E-4 - 3.00E-2) | |
| Proteobacteria | Gammaproteobacteria | Enterobacterales | ND | ND | ND | 5.06 ± 1.30 | 0.005 | 3.10E-1 (1.53E-1 - 3.52E-1) | | 8.60E-3 (6.48E-4 - 1.88E-2) | |
| Proteobacteria | Gammaproteobacteria | Enterobacterales | Enterobacteriaceae | Escherichia | sp 3 2 53FAA | 5.02 ± 1.71 | 0.045 | 6.70E-3 (3.18E-3 - 1.46E-2) | | 1.55E-4 (0.00E+0 - 5.69E-4) | |
| Proteobacteria | Gammaproteobacteria | Enterobacterales | Enterobacteriaceae | Klebsiella | pneumoniae | 4.99 ± 1.73 | 0.049 | 5.29E-3 (3.50E-3 - 8.06E-2) | | 7.53E-4 (1.14E-4 - 3.35E-3) | |
| Proteobacteria | Gammaproteobacteria | Enterobacterales | Enterobacteriaceae | Pluralibacter | gergoviae | 4.97 ± 1.61 | 0.038 | 6.38E-4 (1.86E-4 - 8.64E-4) | | 0.00E+0 (0.00E+0 - 6.05E-5) | |
| Proteobacteria | Gammaproteobacteria | Enterobacterales | Enterobacteriaceae | Enterobacter | hormaechei | 4.79 ± 1.56 | 0.038 | 2.19E-3 (1.63E-3 - 5.98E-3) | | 5.45E-5 (0.00E+0 - 3.57E-4) | |
| Proteobacteria | Gammaproteobacteria | Enterobacterales | Enterobacteriaceae | Escherichia | sp 1 1 43 | 4.70 ± 1.45 | 0.029 | 5.44E-3 (2.21E-3 - 2.02E-2) | | 2.68E-4 (6.87E-5 - 3.71E-3) | |
| Proteobacteria | Gammaproteobacteria | Enterobacterales | Enterobacteriaceae | Escherichia | sp KTE31 | 4.58 ± 1.55 | 0.045 | 8.37E-3 (3.74E-3 - 1.96E-2) | | 2.38E-4 (4.19E-5 - 2.78E-3) | |
| **Proteobacteria** | **Betaproteobacteria** | **Burkholderiales** | **Alcaligenaceae** | **Achromobacter** | **sp ATCC35328** | 4.50 ± 1.44 | 0.037 | 1.79E-2 (4.59E-3 - 2.07E-2) | | 4.69E-4 (1.67E-4 - 2.96E-3) | |
| Proteobacteria | Gammaproteobacteria | Enterobacterales | Enterobacteriaceae | Citrobacter | koseri | 4.33 ± 1.43 | 0.043 | 5.90E-4 (3.16E-4 - 1.49E-3) | | 1.00E-4 (5.33E-5 - 1.73E-4) | |
| **Proteobacteria** | **Gammaproteobacteria** | **Enterobacterales** | **Enterobacteriaceae** | **Shigella** | **dysenteriae** | 4.33 ± 1.33 | 0.029 | 1.06E-1 (4.18E-2 - 1.99E-1) | | 4.02E-3 (1.14E-3 - 3.67E-2) | |
| Proteobacteria | Gammaproteobacteria | Enterobacterales | Enterobacteriaceae | Escherichia | sp KTE114 | 4.14 ± 1.39 | 0.045 | 8.31E-3 (3.25E-3 - 3.36E-2) | | 4.40E-4 (2.22E-4 - 2.53E-3) | |
| **Proteobacteria** | **Gammaproteobacteria** | **Enterobacterales** | **Enterobacteriaceae** | **Shigella** | **flexneri** | 4.07 ± 1.31 | 0.037 | 2.10E-1 (8.21E-2 - 3.47E-1) | | 4.49E-3 (1.14E-3 - 5.61E-2) | |
| **Proteobacteria** | **Gammaproteobacteria** | **Enterobacterales** | **Enterobacteriaceae** | **Shigella** | **sonnei** | 4.01 ± 1.33 | 0.045 | 1.53E-1 (2.80E-2 - 2.45E-1) | | 4.65E-3 (1.69E-3 - 4.33E-2) | |
| Proteobacteria | ND | ND | ND | ND | ND | 3.77 ± 1.27 | 0.045 | 3.02E-1 (1.63E-1 - 5.51E-1) | | 8.29E-3 (3.48E-3 - 9.10E-2) | |
| **Lower in MR** | | | | | | | | | | | |
| Actinobacteria | Actinobacteria | Actinomycetales | Actinomycetaceae | Actinomyces | timonensis | -8.79 ± 2.72 | 0.028 | 0.00E+0 (0.00E+0 - 0.00E+0) | | 0.00E+0 (0.00E+0 - 5.04E-4) | |
| **Firmicutes** | **Bacilli** | **Lactobacillales** | **Lactobacillaceae** | **Lactobacillus** | **sakei** | -6.93 ± 1.74 | 0.005 | 7.61E-3 (3.12E-3 - 3.17E-2) | | 1.57E-2 (2.26E-3 - 3.37E-1) | |
| **Firmicutes** | **Bacilli** | **Lactobacillales** | **Streptococcaceae** | **Lactococcus** | **piscium** | -4.86 ± 1.35 | 0.012 | 1.53E-4 (9.06E-5 - 3.28E-4) | | 6.48E-3 (1.03E-3 - 3.05E-2) | |
| **Firmicutes** | **Bacilli** | **Lactobacillales** | **Streptococcaceae** | **Streptococcus** | **thermophilus** | -4.81 ± 1.65 | 0.046 | 5.93E-4 (3.38E-4 - 5.28E-3) | | 9.92E-3 (3.09E-4 - 6.70E-2) | |
| **Firmicutes** | **Bacilli** | **Lactobacillales** | **Streptococcaceae** | **Streptococcus** | **parauberis** | -4.47 ± 1.54 | 0.047 | 2.25E-4 (9.32E-5 - 3.47E-4) | | 9.65E-4 (1.82E-4 - 1.49E-2) | |
| Firmicutes | Clostridia | Clostridiales | Lachnospiraceae | Anaerostipes | hadrus | -3.43 ± 1.16 | 0.045 | 4.78E-4 (4.02E-4 - 7.92E-4) | | 3.06E-3 (1.65E-3 - 6.49E-3) | |

* Log_2_FC = 1 represents 2 fold-change of MR:LR

** P values were adjusted using the false discovery rate

FC: fold change (HR/LR), ND: no data (unknown)
