# Supplementary material for: The microbiota of healthy dogs demonstrates individualized responses to synbiotic supplementation in a randomized controlled trial
Source: Anim Microbiome. 2021 May 10;3:36. doi: 10.1186/s42523-021-00098-0 (PMC8111948; doi:10.1186/s42523-021-00098-0)
Supplement: Supplementary file 12 — Additional file 12: Table S11A. Gut bacteria from samples collected at week 4 that were significantly different in the differential abundance analysis (|fold change| ≥ 2 and p < 0.05) between high-responders (HR, n = 8) and mid-responders (MR, n = 7). Species in red were identified with the same trend when HR was compared with low-responders (LR). Table S11B. Gut bacteria from samples collected at week 4 that were significantly different in the differential abundance analysis (|fold change| ≥ 2 and p < 0.05) between mid-responders (MR, n = 7) and low-responders (LR, n = 8). Species in red were identified with the same trend when high-responders (HR) was compared with LR. [file 42523_2021_98_MOESM12_ESM.docx]

# **Supplemental Table 11A.** Gut bacteria from samples collected at week 4 that were significantly different in the differential abundance analysis (|fold change| ≥ 2 and p < 0.05) between high-responders (HR, n = 8) and mid-responders (MR, n = 7). Species in red were identified with the same trend when HR was compared with low-responders (LR).

| **Phylum** | **Class** | **Order** | **Family** | **Genus** | **Species** | **HR vs MR** | | **Relative abundances, in %** | |
| --- | --- | --- | --- | --- | --- | --- | --- | --- | --- |
|  |  |  |  |  |  | **Log 2 FC***  **mean ± SE** | **Adjusted p**** | **HR**  **Median (IQR)** | **MR**  **Median (IQR)** |
| **Lower in HR** | | | | | | | | | |
| Proteobacteria | Gammaproteobacteria | Enterobacterales | Morganellaceae | Proteus | mirabilis | -10.84 ± 2.23 | < 0.001 | 4.43E-5 (0.00E+0 - 3.60E-4) | 2.24E-4 (0.00E+0 - 1.66E-1) |
| Actinobacteria | Coriobacteriia | Eggerthellales | Eggerthellaceae | Slackia | piriformis | -8.74 ± 1.98 | 0.001 | 3.77E-5 (0.00E+0 - 1.80E-4) | 6.21E-5 (0.00E+0 - 4.94E-2) |
| Firmicutes | Bacilli | Lactobacillales | Enterococcaceae | Enterococcus | sp kppr-6 | -7.45 ± 2.19 | 0.031 | 0.00E+0 (0.00E+0 - 2.79E-4) | 5.64E-5 (0.00E+0 - 3.35E-2) |
| Firmicutes | Bacilli | Lactobacillales | Enterococcaceae | Enterococcus | hirae | -7.36 ± 1.47 | < 0.001 | 5.38E-3 (3.93E-3 - 1.36E-2) | 6.90E-3 (2.45E-3 - 2.03E+0) |
| **Firmicutes** | **Bacilli** | **Lactobacillales** | **Streptococcaceae** | **Streptococcus** | **ND** | -6.89 ± 1.35 | < 0.001 | 2.22E-2 (9.35E-3 - 2.94E-2) | 3.78E-2 (2.02E-2 - 1.25E+0) |
| Firmicutes | Erysipelotrichia | Erysipelotrichales | Erysipelotrichaceae | Allobaculum | stercoricanis | -6.81 ± 1.49 | < 0.001 | 7.12E-4 (1.16E-4 - 1.37E-3) | 5.59E-4 (4.75E-4 - 2.06E-1) |
| Bacteroidetes | Bacteroidia | Bacteroidales | Bacteroidaceae | Bacteroides | fragilis | -6.75 ± 1.63 | 0.002 | 1.39E-3 (8.71E-4 - 4.20E-3) | 1.14E-2 (4.40E-3 - 3.19E-1) |
| **Firmicutes** | **Bacilli** | **Lactobacillales** | **Streptococcaceae** | **Streptococcus** | **equinus** | -6.39 ± 1.29 | < 0.001 | 8.83E-4 (7.10E-4 - 1.28E-3) | 1.23E-3 (9.83E-4 - 2.52E-2) |
| Firmicutes | Erysipelotrichia | Erysipelotrichales | Erysipelotrichaceae | Catenibacterium | mitsuokai | -6.34 ± 1.58 | 0.003 | 3.34E-3 (1.84E-3 - 7.13E-3) | 1.20E-3 (7.88E-4 - 6.18E-1) |
| Bacteroidetes | Bacteroidia | Bacteroidales | Prevotellaceae | Prevotella | sp Marseille-P4119 | -5.85 ± 1.81 | 0.047 | 0.00E+0 (0.00E+0 - 1.69E-4) | 2.17E-3 (2.79E-5 - 3.69E-3) |
| Firmicutes | Erysipelotrichia | Erysipelotrichales | Erysipelotrichaceae | Erysipelatoclostridium | spiroforme | -5.77 ± 1.51 | 0.007 | 2.69E-5 (0.00E+0 - 2.17E-4) | 3.39E-4 (2.33E-4 - 1.03E-2) |
| **Bacteroidetes** | **Bacteroidia** | **Bacteroidales** | **Bacteroidaceae** | **Bacteroides** | **stercoris** | -5.74 ± 1.36 | 0.002 | 3.07E-3 (1.43E-3 - 1.29E-2) | 2.07E-1 (3.82E-2 - 1.12E+0) |
| Bacteroidetes | Bacteroidia | Bacteroidales | Bacteroidaceae | Bacteroides | cellulosilyticus | -5.02 ± 1.54 | 0.047 | 0.00E+0 (0.00E+0 - 2.51E-5) | 5.64E-4 (2.83E-4 - 1.34E-3) |

* Log_2_FC = 1 represents 2 fold-change of HR:MR

** P values were adjusted using the false discovery rate

FC: fold change (HR/LR), ND: no data (unknown)

#

#

# **Supplemental Table 11B.** Gut bacteria from samples collected at week 4 that were significantly different in the differential abundance analysis (|fold change| ≥ 2 and p < 0.05) between mid-responders (MR, n = 7) and low-responders (LR, n = 8). Species in red were identified with the same trend when high-responders (HR) was compared with LR.

| **Phylum** | **Class** | **Order** | **Family** | **Genus** | **Species** | **Synbiotics Week 4**  **MR vs LR** | | **Relative abundances, in %** | |
| --- | --- | --- | --- | --- | --- | --- | --- | --- | --- |
|  |  |  |  |  |  | **Log 2 FC***  **mean ± SE** | **Adjusted p**** | **MR**  **Median (IQR)** | **LR**  **Median (IQR)** |
| **Higher in MR** | | | | | | | | | |
| Proteobacteria | Gammaproteobacteria | Enterobacterales | Morganellaceae | Proteus | mirabilis | 10.36 ± 2.08 | < 0.001 | 2.24E-4 (0.00E+0 - 1.66E-1) | 2.98E-4 (4.49E-5 - 1.24E-3) |
| Firmicutes | Bacilli | Lactobacillales | Enterococcaceae | Enterococcus | hirae | 9.79 ± 1.57 | < 0.001 | 6.90E-3 (2.45E-3 - 2.03E+0) | 5.62E-4 (3.18E-4 - 1.36E-3) |
| Firmicutes | Bacilli | Lactobacillales | Enterococcaceae | Enterococcus | sp kppr-6 | 8.04 ± 2.14 | 0.016 | 5.64E-5 (0.00E+0 - 3.35E-2) | 2.64E-5 (0.00E+0 - 1.21E-4) |
| **Firmicutes** | **Bacilli** | **Lactobacillales** | **Aerococcaceae** | **Facklamia** | **ND** | 6.59 ± 1.74 | 0.016 | 3.59E-4 (3.37E-4 - 1.24E-3) | 0.00E+0 (0.00E+0 - 0.00E+0) |
| Actinobacteria | Coriobacteriia | Eggerthellales | Eggerthellaceae | Slackia | piriformis | 6.24 ± 1.91 | 0.043 | 6.21E-5 (0.00E+0 - 4.94E-2) | 2.33E-4 (5.10E-5 - 7.49E-4) |
| Firmicutes | Erysipelotrichia | Erysipelotrichales | Erysipelotrichaceae | Massiliomicrobiota | timonensis | 5.19 ± 1.57 | 0.043 | 1.80E-4 (1.12E-4 - 2.51E-3) | 1.52E-4 (0.00E+0 - 3.81E-4) |
| Bacteroidetes | Bacteroidia | Bacteroidales | Bacteroidaceae | Bacteroides | fragilis | 5.13 ± 1.54 | 0.043 | 1.14E-2 (4.40E-3 - 3.19E-1) | 6.88E-3 (4.27E-3 - 7.47E-3) |
| **Firmicutes** | **Bacilli** | **Lactobacillales** | **Enterococcaceae** | **Enterococcus** | **sp HMSC035C10** | 5.02 ± 1.50 | 0.043 | 6.99E-3 (4.27E-3 - 1.75E-2) | 2.87E-4 (4.49E-5 - 7.76E-4) |
| **Firmicutes** | **Bacilli** | **Lactobacillales** | **Enterococcaceae** | **Enterococcus** | **sp HMSC34G12** | 4.96 ± 1.53 | 0.043 | 3.66E-3 (1.83E-3 - 7.34E-3) | 6.73E-5 (0.00E+0 - 1.40E-4) |
| **Lower in MR** | | | | | | | | | |
| Proteobacteria | Gammaproteobacteria | Enterobacterales | Hafniaceae | Hafnia | alvei | -7.50 ± 2.26 | 0.043 | 0.00E+0 (0.00E+0 - 1.86E-4) | 1.18E-4 (0.00E+0 - 4.89E-3) |
| **Firmicutes** | **Bacilli** | **Lactobacillales** | **Streptococcaceae** | **Lactococcus** | **lactis** | -7.48 ± 1.44 | < 0.001 | 7.31E-4 (4.20E-4 - 1.69E-3) | 2.63E-2 (1.76E-3 - 2.53E-1) |
| Firmicutes | Bacilli | Lactobacillales | Leuconostocaceae | Weissella | paramesenteroides | -6.63 ± 1.93 | 0.043 | 0.00E+0 (0.00E+0 - 0.00E+0) | 6.03E-4 (2.76E-4 - 1.17E-3) |
| **Firmicutes** | **Bacilli** | **Lactobacillales** | **Streptococcaceae** | **Streptococcus** | **gallolyticus** | -5.32 ± 1.64 | 0.043 | 3.91E-4 (9.64E-5 - 5.04E-3) | 2.57E-2 (6.67E-4 - 9.61E-2) |

* Log_2_FC = 1 represents 2 fold-change of MR:LR

** P values were adjusted using the false discovery rate

FC: fold change (HR/LR), ND: no data (unknown)
